# Enabling Visible Light Sensitization of Yb^III^, Nd^III^ and Er^III^ in Dimeric Ln^III^/Ga^III^ Metallacrowns through Functionalization with Ru^II^ Complexes for NIR‐II Multiplex Imaging

**DOI:** 10.1002/anie.202416101

**Published:** 2024-11-06

**Authors:** Codruţa C. Bădescu‐Singureanu, Anton S. Nizovtsev, Vincent L. Pecoraro, Stéphane Petoud, Svetlana V. Eliseeva

**Affiliations:** ^1^ Centre de Biophysique Moléculaire CNRS UPR 4301 Université d'Orléans Rue Charles Sadron Orléans 45071 France; ^2^ Nikolaev Institute of Inorganic Chemistry Siberian Branch of Russian Academy of Sciences 3 Academician Lavrentiev Avenue Novosibirsk 630090 Russia; ^3^ Novosibirsk State University 2 Pirogov Street Novosibirsk 630090 Russia; ^4^ Department of Chemistry Willard H. Dow Laboratories University of Michigan Ann Arbor Michigan 48109-1055 United States

**Keywords:** lanthanide, ruthenium, metallacrown, near-infrared luminescence, metal-to-ligand charge transfer

## Abstract

Multiplex imaging in the second near‐infrared window (NIR‐II, 1000–1700 nm) provides exciting opportunities for more precise understanding of biological processes and more accurate diagnosis of diseases by enabling real‐time acquisition of images with improved contrast and spatial resolution in deeper tissues. Today, the number of imaging agents suitable for this modality remains very scarce. In this work, we have synthesized and fully characterized, including theoretical calculations, a series of dimeric Ln^III^/Ga^III^ metallacrowns bearing Ru^II^ polypyridyl complexes, **LnRu‐3** (Ln=Y^III^, Yb^III^, Nd^III^, Er^III^). Relaxed structures of **YRu‐3** in the ground and the excited electronic states have been calculated using dispersion‐corrected density functional theory methods. Detailed photophysical studies of **LnRu‐3** have demonstrated that characteristic emission signals of Yb^III^, Nd^III^ and Er^III^ in the NIR‐II range can be sensitized upon excitation in the visible range through Ru^II^‐centered metal‐to‐ligand charge transfer (MLCT) states. We have also showed that these NIR‐II signals are unambiguously detected in an imaging experiment using capillaries and biological tissue‐mimicking phantoms. This work opens unprecedented perspectives for NIR‐II multiplex imaging using Ln^III^‐based molecular compounds.

## Graphical abstract

**LnRu‐3** dimeric Ln^III^/Ga^III^ metallacrows bearing four Ru^II^ chromophoric complexes have been synthesized and proved to exhibit characteristic emissions of Yb^III^, Nd^III^ and Er^III^ in the NIR‐II region upon excitation with visible light through metal‐to‐ligand charge transfer (MLCT) states. The unambiguous detection of different Ln^III^ transitions has been demonstrated by NIR‐II luminescence imaging of capillaries containing solutions of **LnRu‐3**.

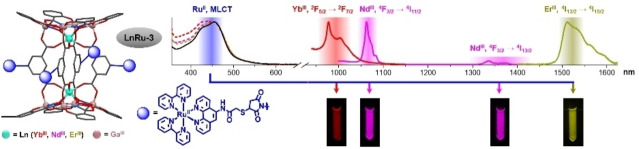

Optical imaging in the second near‐infrared window (NIR‐II, 1000–1700 nm) has become in recent years an essential tool for clinical diagnosis, biomedical applications and fundamental research.[^1^, ^2^, ^3^, ^4^] Due to reduced absorption, scattering and the negligible presence of native biological residual fluorescence (autofluorescence), NIR‐II imaging allows deeper penetration depths and higher detection sensitivity for acquisition of images with improved contrast and spatial resolution. Multiplex NIR‐II imaging is attracting an increasing attention since it allows the simultaneous real‐time visualization of multiple biological species or markers for even more accurate diagnosis of diseases and deeper understanding of complex biological processes.[^5^, ^6^, ^7^] This innovative approach requires specific imaging agents that possess sufficiently narrow, nonoverlapping emission bands to allow spectral discrimination. However, today, the number of NIR‐II imaging agents[^4^, ^8^, ^9^, ^10^, ^11^] and, in particular, the ones that can be used for multiplex NIR‐II imaging is very limited. Most of the currently reported imaging agents are based on nanomaterials,[^12^, ^13^, ^14^, ^15^, ^16^] while only a few examples of molecular probes[^17^, ^18^, ^19^, ^20^, ^21^, ^22^, ^23^] have been described.^[24]^

The unique spectroscopic properties of lanthanide(III) ions (Ln^III^), in particular, the sharp and characteristic emission bands throughout the visible and the near‐infrared (NIR) spectral regions, that have minimal overlap and sensitivity to experimental conditions, make them ideal candidates for the design of NIR‐II multiplex imaging agents.[^25^, ^26^] Indeed, the great potential of Ln^III^ for such applications has been demonstrated in several examples using doped inorganic nanomaterials.[^5^, ^27^, ^28^, ^29^] In contrast, the number of NIR‐II Ln^III^‐based molecular compounds suitable for multiplex imaging remains very scarce. One notable example is an Er^III^ complex formed with a derivative of bacteriochlorin.^[30]^

Two main challenges have to be addressed when designing NIR‐II Ln^III^‐based molecular imaging agents.[^31^, ^32^] The first one originates from the forbidden nature of the majority of Ln^III^ *f‐f* transitions that is reflected in their low molar absorption coefficients. To overcome this limitation and drastically increase the number of emitted photons, the ‘antenna effect’, i.e. Ln^III^ sensitization through energy transfer from highly absorbing chromophoric groups located in close proximity to the Ln^III^, has been successfully tested.[^33^, ^34^] The most widely used chromophores are organic molecules.[^35^, ^36^, ^37^, ^38^, ^39^] The alternative Ln^III^ sensitization strategy is based on the use of the *d*‐transition metal complexes by creating *d‐f* heterometallic assemblies.[^40^, ^41^, ^42^] This approach has been initially suggested by *Van Veggel* and co‐workers in 2000.^[43]^ It is significantly less explored but offers several complementary advantages, namely, (i) the presence of broad and intense metal‐to‐ligand charge transfer (MLCT) absorption bands that can be tuned by varying the nature of the *d*‐metal and/or of the organic ligand; (ii) an efficient intersystem crossing due to the heavy‐atom effect[^44^, ^45^] and (iii) longer luminescence lifetimes inducing an enhanced population of Ln^III^ excited states; (iv) kinetic inertness and (v) high photochemical stability.^[40]^ Among different *d*‐transition metal complexes suitable for the sensitization of Ln^III^ emission in the NIR range, Ru^II^ compounds formed with polypyridyl ligands are particularly attractive.^[46]^ Apart from their large molar absorption coefficients, biocompatibility and suitability for optical imaging applications,[^47^, ^48^, ^49^, ^50^, ^51^, ^52^] Ru^II^ complexes have been successfully used as anticancer[^53^, ^54^, ^55^, ^56^] and antimicrobial[^57^, ^58^, ^59^] drugs, or as photosensitizers in photodynamic therapy.[^46^, ^60^, ^61^, ^62^, ^63^, ^64^] Ru^II^ complexes, in combination with photochromic dithienylethenes and Yb^III^ complexes, have also been used to create tunable NIR photoswitches.[^65^, ^66^, ^67^] Studies devoted to the NIR‐emitting Ru^II^‐Ln^III^ assemblies have demonstrated that Ru^II^‐centered MLCT states are best suited for the sensitization of the Nd^III^ emission followed by Yb^III^, while Ru^II^‐Er^III^ complexes usually exhibit very low NIR emission intensity.[^67^, ^68^, ^69^, ^70^, ^71^, ^72^, ^73^, ^74^, ^75^, ^76^, ^77^, ^78^] These conclusions have been based on the analysis of the Ru^II^‐centered emission spectra and luminescence lifetimes, while quantitative parameters characterizing Ln^III^‐centered emission in Ru^II^‐Ln^III^ assemblies have not been, or have rarely been, reported.

The second challenge to design NIR‐II imaging agents based on Ln^III^ is to overcome the high probability of non‐radiative deactivation of their excited states.[^33^, ^34^] Recently, we have reported several families of monomeric and dimeric Ln^III^/Ga^III^ metallacrowns (MCs) that possess outstanding abilities to protect and sensitize the characteristic luminescence signals of all visible and NIR‐emitting Ln^III^,^[79]^ exhibit a tunability of photophysical parameters, signals in computed tomography,^[80]^ thermal sensitivity[^81^, ^82^] or white‐light emission properties^[83]^ by controlling the nature of the organic ligands that constitute them or the symmetry around Ln^III^. Dimeric Ln^III^/Ga^III^ MCs with the general composition [Ln_2_Ga_8_(shi)_8_(L)_4_] (where shi^3−^, salicylhydroximate, is a core ligand, whereas L^2−^ is a derivative of isophthalate that serves as a bridging ligand)[^33^, ^79^, ^84^] remain intact in solution, sustain biological conditions^[33]^ and are particularly versatile through the functionalization of their bridging ligands.^[85]^ However, Ln^III^ emission in the currently reported Ln^III^/Ga^III^ MCs can be sensitized only with UV light that interferes with biological entities, strongly limiting their biological applications. Therefore, NIR‐II agents with excitation in the visible/NIR range are preferred, in particular for *in vivo* imaging, because of deeper penetration depths and diminished scattering.[^30^, ^38^, ^86^, ^87^]

We have designed and synthesized a series of Ru^II^ polypyridyl‐Ln^III^/Ga^III^ MCs with the general composition [Ln_2_Ga_8_(shi)_8_(bpy_2_RuPhenMip)_4_][PF_6_]_6_ (**LnRu‐3**, Ln=Yb^III^, Er^III^ and Nd^III^; Scheme 1) that combines the advantages of the Ru^II^ polypyridyl complexes with the ones of the dimeric MC scaffold. The diamagnetic Y^III^ analogue has been synthesized to perform ^1^H and ^13^C nuclear magnetic resonance (NMR) studies, including 2D diffusion‐ordered NMR spectroscopy (DOSY) and to serve as a control for the photophysical characterization. Ln^III^‐ and Ru^II^‐centered excitation and emission spectra, quantum yields and luminescence lifetimes have been measured, analyzed and correlated with parameters obtained from the theoretical calculations. Multiplex NIR‐II imaging experiments have been performed on **LnRu‐3** solutions in DMSO, aqueous or cell culture media.

**Scheme 1:**
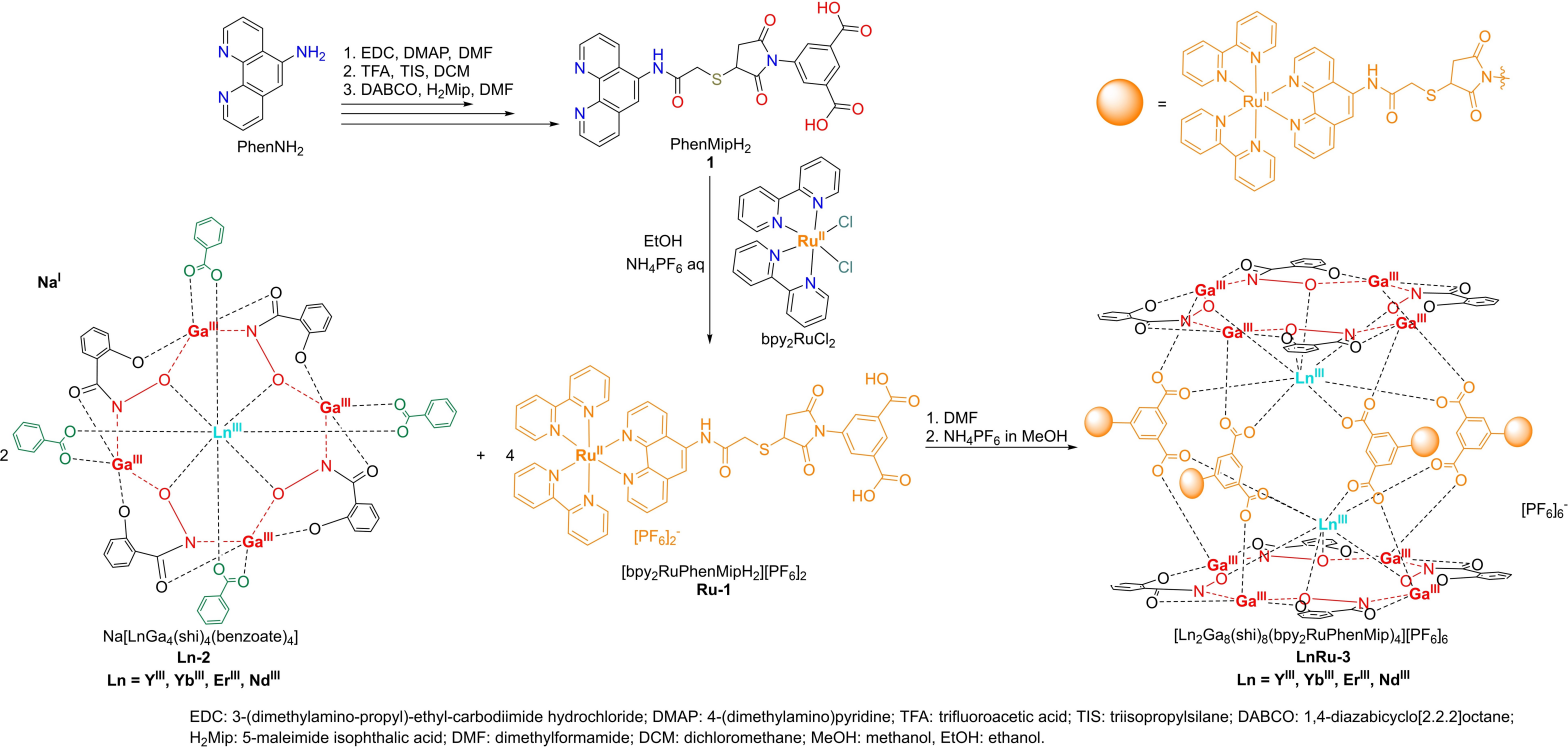
Synthesis of **LnRu‐3** MCs (for more experimental details see Supporting Information).

There are two main synthetic strategies to obtain dimeric [Ln_2_Ga_8_(shi)_8_(L)_4_] MCs: (i) by the self‐assembly reaction between Ln^III^ and Ga^III^ nitrates, H_3_shi and H_2_L ligands in the 2 : 8 : 8 : 4 ratio, or (ii) by substituting benzoate groups by derivatives of isophthalates in the corresponding monomeric [LnGa_4_(shi)_4_(benzoate)_4_] MCs.^[33]^ Therefore, the main challenging step towards the formation of **LnRu‐3** assemblies, was to design and synthesize the isophthalic acid functionalized with the Ru^II^ polypyridyl complex (**Ru‐1**, Scheme 1, S1) that will prevent steric hindrance and allow the formation of the dimeric Ln^III^/Ga^III^ MC scaffold while being stable under synthetic and purification conditions. Our previous studies have demonstrated that the most reactive and selective derivative of isophthalic acid amenable for further functionalization is the 5‐maleimidoisophthalic acid (**H_2_Mip**, Scheme S4).^[85]^ Therefore, we have synthesized the thiol‐bearing 1,10‐phenanthroline from the 5‐amino derivative (**PhenNH_2_**) using amide coupling and protection/deprotection reactions (Schemes S2–S5). The following assembly of the obtained ligand with **H_2_Mip** through a Michael addition reaction gave the desired ligand **1** (Scheme S5). The **Ru‐1** complex was then obtained by the interaction of **1** with bis(2,2’‐bipyridyl)Ru^II^‐dichloride (bpy_2_RuCl_2_)^[88]^ upon reflux in ethanol under inert atmosphere (Scheme S6). Finally, **LnRu‐3** MCs were synthesized using an adapted procedure by the interaction of the corresponding monomeric **Ln‐2** MCs with **Ru‐1** in 2 : 4 ratio in DMF^[85]^ and isolated using a saturated solution of NH_4_PF_6_ (Scheme 1). It should be noted that this reaction should be performed in mild conditions since **Ru‐1** is prone to degradation in the presence of strong bases.

The obtained **LnRu‐3** were characterized by elemental analysis, electrospray ionization‐ion trap‐mass spectrometry (ESI‐IT‐MS, Figures S23–S26) and Fourier‐transform infrared (FTIR, Figures S27–S30) spectroscopy. ESI‐IT‐MS spectra of **LnRu‐3** revealed, in addition to the main molecular peak, a fragmentation pattern that is explained by the presence of labile bonds, i.e. −C−NH−CO− and −C−S−C−, on the bridging isophthalate ligands. ^1^H (Figure S15) and ^13^C (Figure S16) NMR spectra of the diamagnetic Y^III^ analogues were acquired and analyzed. Moreover, to prove the long‐term stability and the integrity of the **LnRu‐3** in DMSO solution and in the presence of D_2_O, 2D DOSY ^1^H NMR analyses were performed on **YRu‐3** (Figures S17, S19, S21). It was confirmed that **YRu‐3** is stable and remains intact in solutions by revealing species with single and similar diffusion coefficients *D*, i.e. 0.9 ⋅ 10^−10^ m^2^ s^−1^ in [D_6_]DMSO (Figures S18, S20) *vs*. 1.1 ⋅ 10^−10^ m^2^ s^−1^ in D_2_O : [D_6_]DMSO (Figure S22).

Photophysical properties of **LnRu‐3** (Ln=Y^III^, Yb^III^, Er^III^ and Nd^III^) were studied in air‐saturated DMSO, H_2_O‐DMSO (99 : 1) and D_2_O‐DMSO (99 : 1) solutions at room temperature. Absorption spectra of **LnRu‐3** (Figures S32, S33, Table S1) are dominated by broad ligand‐centered bands in the UV range (<360 nm) which are attributed to the combination of π→π* transitions within the MC scaffold, 1,10‐phenanthroline and 2,2’‐bipyridine ligands. In addition, an intense broad band centered at 455 nm (*ϵ*_455nm_=(3.9–5.2) ⋅ 10^4^ M^−1^ cm^−1^) due to the Ru^II^‐based MLCT d→π* transitions is observed in the visible range (Figure 1, top). The presence of the MLCT band is typical for Ru^II^ complexes formed with polypyridyl ligands.^[89]^ The general envelope of the absorption spectra of **LnRu‐3** is independent of the nature of the Ln^III^ and of the solvent used, and remains unchanged after extended (7 days) storage in solution (Figure S34). Upon excitation of DMSO and aqueous solutions of **LnRu‐3** into the MLCT band at 455 nm, characteristic emissions of Yb^III^, Er^III^ and Nd^III^ in the NIR‐II range are generated (Figures 1, top, S35, S36). **YbRu‐3** exhibits emission in the range 950–1075 nm centered at 978 nm and assigned to the ^2^F_5/2_→^2^F_7/2_ transition. In the case of **ErRu‐3**, a luminescence band with a maximum at 1512 nm due to the ^4^I_13/2_→^4^I_15/2_ transition is observed in the range 1465–1620 nm. Two emission bands with maxima at 1063 and 1336 nm are detected for **NdRu‐3** in the range 1040–1110 nm (^4^F_3/2_→^4^I_11/2_) and 1310–1400 nm (^4^F_3/2_→^4^I_13/2_), respectively. Excitation spectra of **LnRu‐3** (Ln=Yb^III^, Er^III^ and Nd^III^) collected upon monitoring the main transitions of the corresponding Ln^III^ coincide with the absorption ones (Figure 1, top). Such behavior further confirms the ability of the Ru^II^‐based MLCT states to sensitize all three NIR‐emitting Ln^III^ in **LnRu‐3**. In addition to the Ln^III^‐centered transitions in the NIR range, the excitation at 455 nm of all the studied **LnRu‐3** leads to a broad emission in the range 530–850 nm that arises from the MLCT states (Figures S35, S36). Moreover, we demonstrated that Ln^III^‐centered and Ru^II^‐based excitation and emission spectra measured on solutions of **YbRu‐3** and **NdRu‐3** in Opti‐MEM^TM^‐DMSO (99 : 1) cell culture medium (Figure S37) are similar to the ones observed in DMSO or in aqueous solutions. Absolute quantum yields and luminescence lifetimes of both Ln^III^‐centered ($Q_{Ln}^{MLCT}$ , $\tau_{obs}^{Ln}$ ) and Ru^II^‐based ($Q_{MLCT}^{MLCT}$ , $\tau_{obs}^{MLCT}$ ) transitions were acquired and analyzed (Tables 1, S2). Luminescence decay curves measured upon monitoring the main transitions of Ln^III^ in the NIR range are best fitted with mono‐exponential functions confirming the presence of only one type of Ln^III^‐emitting species in solution. Using the values of $\tau_{obs}^{Ln}$ measured on solutions of **YbRu‐3** and **NdRu‐3** in D_2_O and H_2_O, and phenomenological equations,[^90^, ^91^] it was confirmed that no solvent molecule is directly coordinated to Ln^III^ in solutions of **LnRu‐3** complexes. Nevertheless, the high probability of non‐radiative deactivation through overtones of high energy O−H vibrations precluded the observation of Er^III^‐centered emission band from the H_2_O solution of **ErRu‐3**, while Er^III^ signals were clearly detected in D_2_O and DMSO solutions. In general, for a specific Ln^III^, the values of $\tau_{obs}^{Ln}$ and $Q_{Ln}^{MLCT}$ are the highest for the solutions of **LnRu‐3** in DMSO followed by those in D_2_O and H_2_O.^[79]^ Similar values of $Q_{Ln}^{MLCT}$ were found for solutions of **YbRu‐3** and **NdRu‐3** in H_2_O while they increase in the order **ErRu‐3**<**YbRu‐3**<**NdRu‐3** or **ErRu‐3**<**NdRu‐3**<**YbRu‐3** in DMSO or D_2_O, respectively. The values of $Q_{MLCT}^{MLCT}$ decrease by a factor of 9–13 times when going from solutions of **LnRu‐3** in DMSO to those in H_2_O or D_2_O. In general, quantitative characteristics of Ln^III^‐ and MLCT‐centered emissions are comparable to the ones reported for Ln^III^/Ga^III^ MCs[^33^, ^79^, ^80^] or for Ru^II^ complexes formed with polypyridyl ligands, respectively.^[89]^ No values of $Q_{Ln}^{MLCT}$ have been reported so far for NIR‐emitting Ln^III^‐Ru^II^ complexes, while the number of studies describing $\tau_{obs}^{Ln}$ in aqueous or non‐deuterated solvents is very scarce.[^72^, ^92^] For the solution in D_2_O, the value of $\tau_{obs}^{Ln}$ of **YbRu‐3** is comparable to the one of the Yb^III^‐Ru^II^ host–guest assembly (21.9 μs),^[72]^ while it is 3.3‐times longer than the value found for the Yb^III^‐cyclen‐Ru^II^ conjugate.^[92]^ For the Nd^III^ analogue of the latter complex, $\tau_{obs}^{Ln}$ is 11‐times shorter (0.21 μs) than the one found for **NdRu‐3**. In the case of Er^III^‐Ru^II^ complexes, the longest value of $\tau_{obs}^{Ln}$ was observed for the solution of the heterotrinuclear Er^III^‐Ru^II^_2_ assembly in CD_3_OD (0.852 μs).^[70]^ The latter is 8‐times shorter than the one recorded for the **ErRu‐3** in D_2_O.

**Figure 1:**
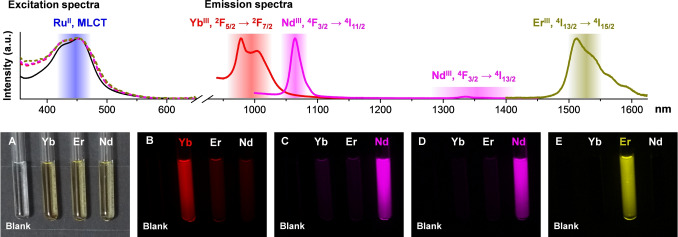
Photophysical and NIR‐II imaging results obtained on solutions of **LnRu‐3** in H_2_O‐DMSO (99 : 1, 14 μM; Ln=Yb^III^ (red), Nd^III^ (magenta)) or D_2_O‐DMSO (99 : 1, 14 μM; Er^III^ (green‐yellow)). (Top) Excitation (dashed colored traces; *λ*_em_=980 nm (Yb^III^), 1064 nm (Nd^III^) or 1525 nm (Er^III^)) and emission (solid colored traces; *λ*_ex_=455 nm) spectra. The absorption spectrum of **YbRu‐3** (solid black trace) is overlapped for comparison with the excitation spectra. Shaded rectangles represent the range of wavelengths covered by different bandpass filters used in the NIR‐II imaging experiments (see below). (Bottom) Color images of quartz capillaries (2 mm internal diameter) filled with solutions of **LnRu‐3** (A) and NIR‐II luminescence images obtained upon excitation with a light selected using a 447 nm bandpass 60 nm filter and monitoring emission signals of Yb^III^ (B: 996 nm bandpass 70 nm filter, *τ*_exp_=0.5 s), Nd^III^ (C: 1065 nm bandpass 30 nm filter, *τ*_exp_=0.5 s; D: 1365 nm bandpass 130 nm filter, *τ*_exp_=2 s) or Er^III^ (E: 1530 nm bandpass 50 nm filter, *τ*_exp_=2 s). A capillary filled with H_2_O‐DMSO (99 : 1) was used as a blank.

**Table 1:** Photophysical parameters for **LnRu‐3** (Ln=Yb^III^, Er^III^, Nd^III^).^[a]^

MC	Solvent	$Q_{Ln}^{MLCT}\;$ (10^−2 ^%)^[b]^	$\tau_{obs}^{Ln}$ (μs)^[c]^	$Q_{MLCT}^{MLCT}$ (%)^[b]^	$\tau_{obs}^{MLCT}$ (μs)^[d]^
**YbRu‐3**	DMSO^[e]^	5.23(1)	54.2(1)	9.74(2)	0.74(1)
	H_2_O^[f]^	0.552(3)	9.20(3)	0.728(2)	0.59(1)
	D_2_O^[g]^	4.66(1)	20.34(2)	0.952(1)	0.72(6)
**ErRu‐3**	DMSO^[e]^	0.307(4)	9.23(7)	9.96(3)	0.78(3)
	D_2_O^[g]^	0.137(2)	6.80(5)	0.927(6)	0.68(5)
**NdRu‐3**	DMSO^[e]^	8.50(6)	3.11(1)	8.67(1)	0.67(5)
	H_2_O^[f]^	0.579(6)	0.74(1)	0.696(8)	0.51(3)
	D_2_O^[g]^	1.21(1)	2.35(7)	0.919(4)	0.70(3)

[a] Data collected for solutions in air‐saturated solvents at room temperature. 2σ values between parentheses; relative errors: *τ*_obs_, ±2 %; Q , ±10 %. [b] *λ*_ex_=455 nm. [c] *λ*_ex_=355 nm; *λ*_em_=*λ*_em_ (Ln^III^). [d] *λ*_ex_=355 nm; *λ*_em_=610 nm. [e] 14 μM. [f] H_2_O‐DMSO (99 : 1), 10 μM. [g] D_2_O‐DMSO (99 : 1), 10 μM.

To identify molecular geometries of the **LnRu‐3** MCs, we calculated relaxed structures of **YRu‐3** in its ground (S_0_) and excited electronic states (Figures 2A, S43) by using a computational approach based on the semiempirical^[93]^ and dispersion‐corrected density functional theory (DFT)[^94^, ^95^, ^96^, ^97^, ^98^, ^99^, ^100^] methods (see Supporting Information). It was shown that four Ru^II^ polypyridyl ligands occupy positions between the [LnGa_4_(shi)_4_] cores, with two of them predicted to be located higher in order to maximize the number of stabilizing interatomic contacts with the MC scaffold and the neighboring ligands. The relaxation of the excited state retains the molecular shape of **YRu‐3** with minor structural changes of the ligands. To estimate the positions of the ^1^MLCT and ^3^MLCT electronic states, the energies of the lowest singlet‐singlet and singlet‐triplet vertical electronic transitions were calculated for **YRu‐3** within the spin‐orbit coupling time‐dependent DFT.[^101^, ^102^] The obtained energies, *E*(^1^MLCT)=20 300 cm^−1^ and *E*(^3^MLCT)=17 331 cm^−1^, are in good agreement with the ones determined experimentally from the absorption and emission spectra of **YRu‐3**, respectively, 21 780 cm^−1^ and 16 360 cm^−1^ (Figure S38). To visualize the transition characteristics of the excited states in **YRu‐3**, we delineated distributions of the hole‐electron pairs^[103]^ upon S_0_→^1^MLCT and S_0_→^3^MLCT electronic transitions (Figure 2B). It was found that, in both cases, the electron density is mainly transferred from the *d* orbitals of Ru^II^ to π* molecular orbitals of the adjacent 2,2’‐bipyridine or 1,10‐phenanthroline ligands. The hole‐electron analysis allowed the estimation of the donor–acceptor distance (*R*_L_=11.42 Å for ^1^MLCT and 9.04 Å for ^3^MLCT) between the Ru^II^ chromophore and the Ln^III^ (Table S3).

**Figure 2:**
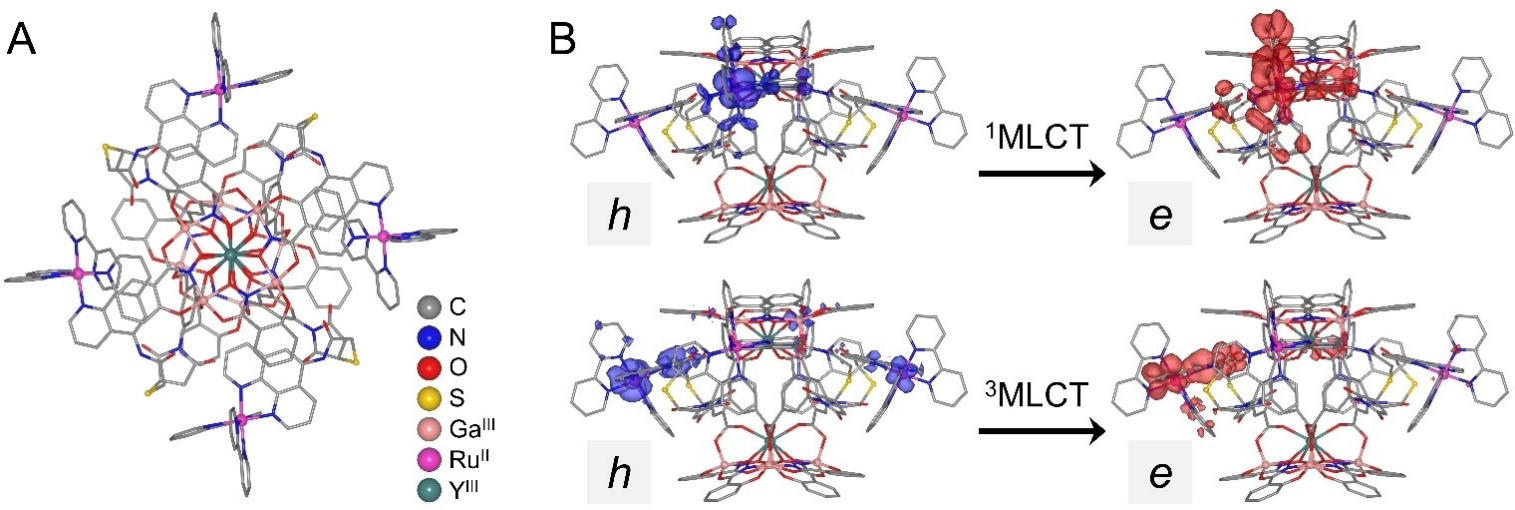
Results of DFT calculations for **YRu‐3**. (A) Optimized structure (top view); (B) hole (*h*) and electron (*e*) distributions upon S_0_→^1^MLCT and S_0_→^3^MLCT electronic transitions (side views). Hydrogens are omitted for clarity.

To get more insights into the energy transfer processes, we performed the analysis of the intramolecular energy transfer (IET) rates using the JOYSpectra web platform assuming the direct population of the MLCT states upon excitation of **LnRu‐3** (Tables S3–S15).^[104]^ It was shown that the ^1^MLCT state is the main feeding source to populate the excited states of Nd^III^ and Er^III^ in **LnRu‐3** while the ^3^MLCT state contributes to the sensitization of Yb^III^ emission arising from the ^2^F_5/2_ level.

To demonstrate the potential of the studied MCs to be used as imaging agents, NIR‐II luminescence images were acquired using capillaries filled with solutions of **LnRu‐3** in aqueous media (Figure 1, bottom) or in DMSO (Figure S40). Upon excitation into MLCT electronic states with light selected with a 447 nm bandpass 60 nm filter, we detected unambiguously the emissions arising from Yb^III^ and Er^III^, as well as two transitions of Nd^III^ using specific bandpass filters centered at 996, 1530, 1065 and 1365 nm, respectively. Moreover, we were able to monitor emission signals of Yb^III^ and Nd^III^ from capillaries containing solutions of **YbRu‐3** or **NdRu‐3** in Opti‐MEM^TM^ cell culture medium (Figure 3B–C, Figure S41B–D) and confirmed the absence of significant changes of NIR‐II intensities after 48 h of storage (Figure S41E–G). Despite the relatively high attenuation of the excitation light selected by the 447 nm bandpass 60 nm filter through the 1 mm biological tissue‐mimicking phantom (92 %, Figure S39B), NIR‐II signals of **YbRu‐3** and **NdRu‐3** were sufficiently intense to acquire images with such an experimental setup (Figure 3D–F). In addition, we demonstrated that NIR‐II images collected on solutions of **LnRu‐3** could be obtained not only upon excitation with light corresponding to the maximum of the MLCT absorption band (455 nm) but also using bandpass filters of longer wavelengths (a 482 nm bandpass 35 nm or a 536 nm bandpass 40 nm, Figure S42).

**Figure 3:**
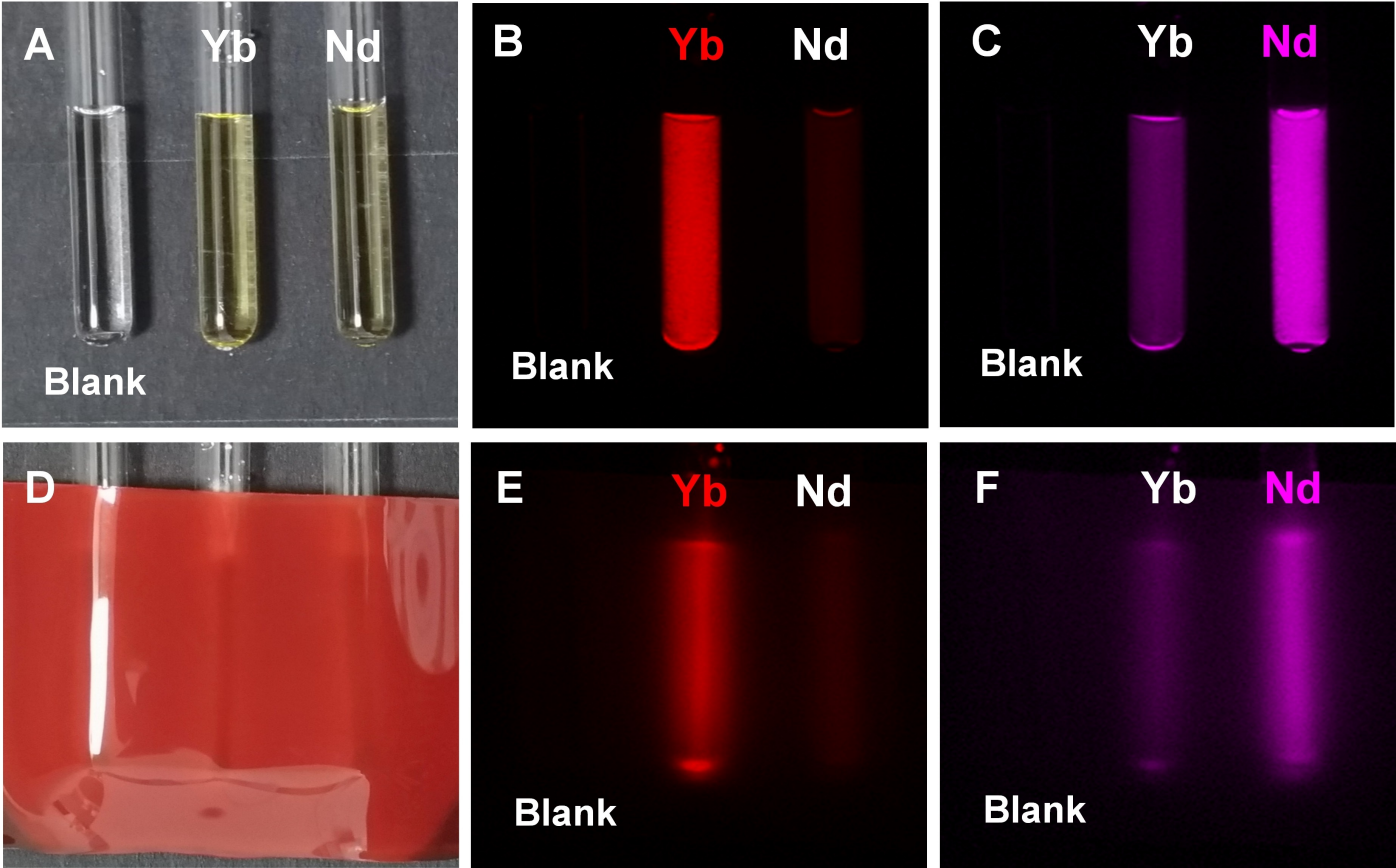
Color images of quartz capillaries (2 mm internal diameter) filled with solutions of **LnRu‐3** (Ln=Yb^III^, Nd^III^) in Opti‐MEM^TM^‐DMSO (99 : 1, 14 μM) (A) and covered with a 1 mm tissue‐mimicking phantom (D). NIR‐II luminescence images obtained upon excitation with a light selected using a 447 nm bandpass 60 nm filter and monitoring emission signals of Yb^III^ (996 nm bandpass 70 nm filter, *τ*_exp_=0.5 s (B) or *τ*_exp_=5 s (E)) or Nd^III^ (1065 nm bandpass 30 nm filter, *τ*_exp_=2 s (C) or *τ*_exp_=10 s (F)). A capillary filled with Opti‐MEM^TM^‐DMSO (99 : 1) was used as a blank.

In summary, we have reported in this work the first examples of dimeric Ln^III^/Ga^III^ MCs functionalized with appended transition metal complexes, **LnRu‐3**, which uniquely enable the sensitization of the characteristic emission signals of three Ln^III^, namely Yb^III^, Nd^III^ and Er^III^, in the NIR‐II region upon excitation into the MLCT electronic states in the visible range. This work is the unique example that reports all the quantitative photophysical parameters, Ln^III^‐ and Ru^II^‐centered quantum yields and luminescence lifetimes, energy transfer efficiencies and rates, for a series of NIR‐emitting *d‐f* hybrid molecular complexes. Experimental results have been correlated with theoretical calculations. NIR‐II imaging experiments using capillaries containing solutions of **LnRu‐3** have demonstrated that four bands arising from three Ln^III^ can be unambiguously distinguished due to their minimal overlap. We have also shown that the NIR‐II luminescence intensities of **YbRu‐3** and **NdRu‐3** in Opti‐MEM^TM^ cell culture medium allow their detection through a 1 mm of biological tissue‐mimicking phantom upon excitation up to 550 nm. We believe that this proof‐of‐concept work opens new perspectives for the creation of multiplex NIR‐II imaging agents based on Ln^III^ molecular compounds.

## Supporting Information

Detailed synthetic procedures, ^1^H and ^13^C NMR, ESI‐IT‐MS and FTIR spectra; additional details about photophysical characterization: absorption and emission spectra, quantum yields and lifetimes; experimental details about NIR‐II imaging and preparation of tissue‐mimicking phantoms; computational details.

The authors have cited additional references within the Supporting Information.[^25^, ^33^, ^42^, ^80^, ^81^, ^84^, ^85^, ^88^, ^95^, ^96^, ^97^, ^98^, ^100^, ^101^, ^102^, ^103^, ^105^]

## Conflict of Interests

The authors declare no conflict of interest.

## Supporting information

As a service to our authors and readers, this journal provides supporting information supplied by the authors. Such materials are peer reviewed and may be re‐organized for online delivery, but are not copy‐edited or typeset. Technical support issues arising from supporting information (other than missing files) should be addressed to the authors.

Supporting Information

## Data Availability

The data that support the findings of this study are available in the supplementary material of this article.
