# Supplementary material for: Enabling Visible Light Sensitization of YbIII, NdIII and ErIII in Dimeric LnIII/GaIII Metallacrowns through Functionalization with RuII Complexes for NIR‐II Multiplex Imaging
Source: Angew Chem Int Ed Engl. 2024 Nov 6;64(3):e202416101. doi: 10.1002/anie.202416101 (PMC11735903; doi:10.1002/anie.202416101)
Supplement: Supplementary file 1 — Supporting Information [file ANIE-64-e202416101-s001.pdf]

Angewandte Chemie
Eine Zeitschrift der Gesellschaft Deutscher Chemiker

Supporting Information

Enabling Visible Light Sensitization of Yb^{III}, Nd^{III} and Er^{III} in Dimeric Ln^{III}/Ga^{III} Metallacrowns through Functionalization with Ru^{II} Complexes for NIR-II Multiplex Imaging

C. C. Bădescu-Singureanu, A. S. Nizovtsev, V. L. Pecoraro, S. Petoud*, S. V. Eliseeva**

Table of content

List of Figures 2

List of Tables 4

Reagents and methods 6

Synthesis of Na[$LnGa_4$(shi)$_4$(benzoate)$_4$] MC monomers (Ln-2, Ln = Y^{III}, Yb^{III}, Er^{III}, Nd^{III}) 7

Synthesis of **Y-2** 7

Synthesis of **Ln-2** (Ln = Yb^{III}, Er^{III}, Nd^{III}) 9

Yb-2 9

Er-2 10

Nd-2 11

Synthesis of [bpy_2RuPhenMipH_2][PF_6]$_2$ (Ru-1) 14

Synthesis of 2-(tritylthio)acetic acid (TrtSAcOH) 14

Synthesis of *N*-(1,10-phenantrolin-5-yl)-2-(trytilthio)acetamide (PhenNHOAcSTrt) 15

Synthesis of 5-maleimide isophthalic acid (H_2Mip) 16

Synthesis of PhenMipH_2 (**1**) 17

Synthesis of **Ru-1** 18

Synthesis of [Ln_2Ga_8(shi)$_8$(bpy_2RuPhenMip)$_4$][PF_6]$_6$ (LnRu-3, Ln = Y^{III}, Yb^{III}, Er^{III}, Nd^{III}) 21

YRu-3 21

YbRu-3 26

ErRu-3 27

NdRu-3 28

Photophysical properties 31

Absorption spectra 31

Emission and excitation spectra, luminescence quantum yields and lifetimes 33

Determination of ^{1}MLCT and ^{3}MLCT 36

Imaging in the NIR-II range 37

Computational details 40

References 47

List of Figures

Figure S1. ^{1}H-NMR spectrum of **Y-2** (DMSO-d_6, 600 MHz). 8
Figure S2. ESI-MS spectrum of **Y-2** in MeOH (negative mode). 9
Figure S3. ESI-MS spectrum of **Yb-2** (negative mode). 10
Figure S4. ESI-MS spectrum of **Er-2** (negative mode). 11
Figure S5. ESI-MS spectrum of **Nd-2** (negative mode). 12
Figure S6. FTIR spectra of **Ln-2** (Ln = Y^{III}, Yb^{III}, Er^{III}, Nd^{III}). 13
Figure S7. ^{1}H-NMR spectrum of TrtSAcOH ($CDCl_3$, 600 MHz). 15
Figure S8. ^{1}H-NMR spectrum of PhenNHOAcSTrt ($CDCl_3$, 600 MHz). 16
Figure S9. ^{1}H-NMR spectrum of H_2Mip (DMSO-d_6, 600 MHz). 17
Figure S10. ^{1}H-NMR spectrum of **1** (DMSO-d_6, 600 MHz). 18
Figure S11. ^{13}C-NMR spectrum of **1** (DMSO-d_6, 600 MHz). 18
Figure S12. ^{1}H-NMR spectrum of **Ru-1** (DMSO-d_6, 600 MHz). 19
Figure S13. ^{13}C-NMR spectrum of **Ru-1** (DMSO-d_6, 600 MHz). 20
Figure S14. ESI-MS spectrum of **Ru-1**. 20
Figure S15. ^{1}H-NMR spectrum of **YRu-3** (DMSO-d_6, 700 MHz). 22
Figure S16. ^{13}C-NMR spectrum of **YRu-3** (DMSO-d_6, 700 MHz). 22
Figure S17. Arrayed DOSY-NMR spectra of **YRu-3** (DMSO-d_6, 700 MHz). 23
Figure S18. Normalized integral intensity as a function of the gradient field strength (G·cm^{-1}) for **YRu-3** (DMSO-d_6, 700 MHz). The diffusion coefficient (D) was estimated using monoexponential decay fitting (R^2 > 0.997) and found to be 0.9·10^{-10} m^{2}s^{-1}. 23
Figure S19. Arrayed DOSY-NMR spectra of **YRu-3** (DMSO-d_6 , 600 MHz) after 3 months of storage in solution. 24
Figure S20. Normalized integral intensity as a function of the gradient field strength (G·cm^{-1}) for **YRu-3** after 3 months of storage in solution (DMSO-d_6, 600 MHz). The diffusion coefficient (D) was estimated using monoexponential decay fitting (R^2 > 0.993) and found to be 0.9·10^{-10} m^{2}s^{-1}. 24
Figure S21. Arrayed DOSY-NMR spectra of **YRu-3** in D_2O:DMSO-d_6 mixture (75% of D_2O, 25% of DMSO-d_6, 600 MHz). 25
Figure S22. Normalized integral intensity as a function of the gradient field strength (G·cm^{-1}) for **YRu-3** in D_2O:DMSO-d_6 mixture (75% of D_2O, 25% of DMSO-d_6, 600 MHz). The diffusion coefficient (D) was estimated using monoexponential decay fitting (R^2 > 0.996) and found to be 1.1·10^{-10} m^{2}s^{-1}. 25
Figure S23. ESI-MS spectrum of **YRu-3**. 26
Figure S24. ESI-MS spectrum of **YbRu-3**. 27
Figure S25. ESI-MS spectrum of **ErRu-3**. 27
Figure S26. ESI-MS spectrum of **NdRu-3**. 28
Figure S27. Comparison of FTIR spectra for **Y-2**, **Ru-1** and **YRu-3**. 29
Figure S28. Comparison of FTIR spectra for **Yb-2**, **Ru-1** and **YbRu-3**. 29
Figure S29. Comparison of FTIR spectra for **Er-2**, **Ru-1** and **ErRu-3**. 30
Figure S30. Comparison of FTIR spectra for **Nd-2**, **Ru-1** and **NdRu-3**. 30
Figure S31. Absorption spectrum of bpy_2RuPhenMipH_2 (**Ru-1**) in DMSO (10 µM). 31
Figure S32. Absorption spectra of **LnRu-3** (Ln = Y^{III}, Yb^{III}, Er^{III}, Nd^{III}) in DMSO (10 µM). 31
Figure S33. Absorption spectra of **LnRu-3** (Ln = Y^{III}, Yb^{III}, Er^{III}, Nd^{III}) in H_2O-DMSO (99:1, 14 µM). 32
Figure S34. Absorption spectra of **LnRu-3** (Ln = Y^{III}, Yb^{III}, Er^{III}, Nd^{III}) in H_2O-DMSO (99:1, 14 µM) after 7 days of storage in solution. 32
Figure S35. Corrected and normalized excitation and emission spectra for solutions of **LnRu-3** (Ln = Y^{III}, Yb^{III}, Er^{III}, Nd^{III}) in DMSO (10 µM). 33
Figure S36. Corrected and normalized excitation and emission spectra for solutions of **LnRu-3** in H_2O-DMSO (99:1, Ln = Y^{III}, Yb^{III}, Nd^{III}, 14 µM) or D_2O-DMSO (99:1, Ln = Er^{III}, 14 µM). 34

Figure S37. Corrected and normalized excitation and emission spectra for solutions of **LnRu-3** in Opti-MEM^{TM}-DMSO (99:1, Ln = Yb^{III}, Nd^{III}, 14 µM). 34
Figure S38. Normalized (top) absorption and (bottom) emission spectra for solution of **YRu-3** in DMSO (10 µM, black traces) and their Gaussian deconvolution (colored traces). Red traces correspond to 0-0 transitions. 36
Figure S39. (A) General scheme of the experimental setup used for NIR-II imaging experiments. (B) Attenuation of light selected using different bandpass (BP) filters upon propagation through a 1 mm tissue-mimicking phantom; percentages correspond to the amount of transmitted light. 37
Figure S40. Photophysical and NIR-II imaging results obtained on solutions of **LnRu-3** (Ln = Yb^{III} (red), Er^{III} (green-yellow), Nd^{III} (magenta)) in DMSO (14 µM). (Top) Excitation (dashed colored traces; λ_{em} = 980 nm (Yb^{III}), 1064 nm (Nd^{III}) or 1525 nm (Er^{III})) and emission (solid colored traces; λ_{ex} = 455 nm) spectra. The absorption spectrum of **YbRu-3** (solid black trace) is overlapped for comparison with the excitation spectra. Shaded rectangular represent the range of wavelengths covered by different bandpass filters used in the NIR-II imaging experiments (vide infra). (Bottom) Color images of quartz capillaries (2 mm internal diameter) filled with solutions of **LnRu-3** (A) and NIR-II luminescence images obtained upon excitation with a light selected using a 447 nm bandpass 60 nm filter and monitoring emission signals of Yb^{III} (B: 996 nm bandpass 70 nm filter, τ_{exp} = 0.5 s), Nd^{III} (C, G: 1065 nm bandpass 30 nm filter, τ_{exp} = 0.5 s; D: 1365 nm bandpass 130 nm filter, τ_{exp} = 2 s) or Er^{III} (E: 1530 nm bandpass 50 nm filter, τ_{exp} = 10 s). 38
Figure S41. Color images of quartz capillaries (2 mm internal diameter) filled with solutions of **LnRu-3** (Ln = Yb^{III}, Nd^{III}) in Opti-MEM^{TM}-DMSO (99:1, 14 µM) (A) and NIR-II luminescence images obtained upon excitation with a light selected using a 447 nm bandpass 60 nm filter and monitoring emission signals of Yb^{III} (B, E: 996 nm bandpass 70 nm filter, τ_{exp} = 1 s), Nd^{III} (C, F: 1065 nm bandpass 30 nm filter, τ_{exp} = 2 s; D, G: 1365 nm bandpass 130 nm filter, τ_{exp} = 5 s). E, F and G images were acquired for the same solutions after 48 h of storage. A capillary filled with Opti-MEM^{TM}-DMSO (99:1) medium was used as a blank. 38
Figure S42. Color images of quartz capillaries (2 mm internal diameter) filled with solutions of **LnRu-3** (Ln = Yb^{III}, Nd^{III}) in Opti-MEM^{TM}-DMSO (99:1, 14 µM) (A) and covered with a 1 mm tissue-mimicking phantom (B). NIR-II luminescence images upon excitation with light selected using a 447 nm bandpass 60 nm filter (C, D), a 482 nm bandpass 35 nm filter (E, F) or a 536 nm bandpass 40 nm filter (G, H). Yb^{III} signal was collected with a 996 nm bandpass 70 nm filter (C, E, G; τ_{exp} = 5 s) while that of Nd^{III} with a 1065 nm bandpass 30 nm filter (D, F, H; τ_{exp} = 10 s). A capillary filled with Opti-MEM^{TM}-DMSO (99:1) medium was used as a blank. 39
Figure S43. Structures of the **YRu-3** complex in its ground (S_0) and excited (T_1) electronic states optimized at SMD-ZORA-PBE-D3(BJ)/ZORA-def2-SVP* level of theory. Top and side views are shown. Hydrogens are omitted for clarity. 41
Figure S44. Schematic energy level diagrams for the (A) **NdRu-3**, (B) **ErRu-3**, and (C) **YbRu-3** complexes. The forward and backward intramolecular energy transfer (IET) pathways with the highest relative contributions are shown by blue and red arrows, respectively (numbers correspond to specific IET pathways, which are listed in Tables S4–S15). S_1 and T_1 labels stand for ^{1}MLCT and ^{3}MLCT states. 42

List of Tables

Table S1. Molar absorption coefficients (ε) for solutions of **Ru-1** and **LnRu-3** (Ln = Y^{III}, Yb^{III}, Er^{III}, Nd^{III}) in DMSO (10 μM) or H_2O-DMSO (99:1, 14 μM). 32
Table S2. Photophysical parameters for solutions of **LnRu-3** (Ln = Y^{III}, Yb^{III}, Er^{III}, Nd^{III}) in DMSO (10 μM), H_2O-DMSO (99:1, 14 μM) and D_2O-DMSO (99:1; 14 μM).[a] 35
Table S3. Experimental and theoretical, singlet (^{1}MLCT, S1MC, cm^{-1}) and triplet (^{3}MLCT, T1MC, cm^{-1}) excited states energies for **LnRu-3** MCs as well as forward (WS and WT) and backward (WbS and WbT) intramolecular energy transfer rates. 41
Table S4. Forward (^{1}MLCT to Nd^{III}) intramolecular energy transfer rates (W, s^{-1}) calculated for the **NdRu-3** complex. Δ (cm^{-1}) is the donor-acceptor energy difference, W(%) is the percentage of the pathway, W_{total} is the sum of the dipole-dipole (W_{d-d}), dipole-multipole (W_{d-m}), and exchange (W_{ex}) rates of the mechanisms. 43
Table S5. Backward (Nd^{III} to ^{1}MLCT) intramolecular energy transfer rates (W, s^{-1}) calculated for the **NdRu-3** complex. Δ (cm^{-1}) is the donor-acceptor energy difference, W(%) is the percentage of the pathway, W_{total} is the sum of the dipole-dipole (W_{d-d}), dipole-multipole (W_{d-m}), and exchange (W_{ex}) rates of the mechanisms. 43
Table S6. Forward (^{3}MLCT to Nd^{III}) intramolecular energy transfer rates (W, s^{-1}) calculated for the **NdRu-3** complex. Δ (cm^{-1}) is the donor-acceptor energy difference, W(%) is the percentage of the pathway, W_{total} is the sum of the dipole-dipole (W_{d-d}), dipole-multipole (W_{d-m}), and exchange (W_{ex}) rates of the mechanisms. 44
Table S7. Backward (Nd^{III} to ^{3}MLCT) intramolecular energy transfer rates (W, s^{-1}) calculated for the **NdRu-3** complex. Δ (cm^{-1}) is the donor-acceptor energy difference, W(%) is the percentage of the pathway, W_{total} is the sum of the dipole-dipole (W_{d-d}), dipole-multipole (W_{d-m}), and exchange (W_{ex}) rates of the mechanisms. 44
Table S8. Forward (^{1}MLCT to Er^{III}) intramolecular energy transfer rates (W, s^{-1}) calculated for the **ErRu-3** complex. Δ (cm^{-1}) is the donor-acceptor energy difference, W(%) is the percentage of the pathway, W_{total} is the sum of the dipole-dipole (W_{d-d}), dipole-multipole (W_{d-m}), and exchange (W_{ex}) rates of the mechanisms. 45
Table S9. Backward (Er^{III} to ^{1}MLCT) intramolecular energy transfer rates (W, s^{-1}) calculated for the **ErRu-3** complex. Δ (cm^{-1}) is the donor-acceptor energy difference, W(%) is the percentage of the pathway, W_{total} is the sum of the dipole-dipole (W_{d-d}), dipole-multipole (W_{d-m}), and exchange (W_{ex}) rates of the mechanisms. 45
Table S10. Forward (^{3}MLCT to Er^{III}) intramolecular energy transfer rates (W, s^{-1}) calculated for the **ErRu-3** complex. Δ (cm^{-1}) is the donor-acceptor energy difference, W(%) is the percentage of the pathway, W_{total} is the sum of the dipole-dipole (W_{d-d}), dipole-multipole (W_{d-m}), and exchange (W_{ex}) rates of the mechanisms. 46
Table S11. Backward (Er^{III} to ^{3}MLCT) intramolecular energy transfer rates (W, s^{-1}) calculated for the **ErRu-3** complex. Δ (cm^{-1}) is the donor-acceptor energy difference, W(%) is the percentage of the pathway, W_{total} is the sum of the dipole-dipole (W_{d-d}), dipole-multipole (W_{d-m}), and exchange (W_{ex}) rates of the mechanisms. 46
Table S12. Forward (^{1}MLCT to Yb^{III}) intramolecular energy transfer rates (W, s^{-1}) calculated for the **YbRu-3** complex. Δ (cm^{-1}) is the donor-acceptor energy difference, W(%) is the percentage of the pathway, W_{total} is the sum of the dipole-dipole (W_{d-d}), dipole-multipole (W_{d-m}), and exchange (W_{ex}) rates of the mechanisms. 47
Table S13. Backward (Yb^{III} to ^{1}MLCT) intramolecular energy transfer rates (W, s^{-1}) calculated for the **YbRu-3** complex. Δ (cm^{-1}) is the donor-acceptor energy difference, W(%) is the percentage of the pathway, W_{total} is the sum of the dipole-dipole (W_{d-d}), dipole-multipole (W_{d-m}), and exchange (W_{ex}) rates of the mechanisms. 47

Table S14. Forward (^{3}MLCT to Yb^{III}) intramolecular energy transfer rates (W, s^{-1}) calculated for the **YbRu-3** complex. Δ (cm^{-1}) is the donor-acceptor energy difference, W(%) is the percentage of the pathway, W_{total} is the sum of the dipole-dipole (W_{d-d}), dipole-multipole (W_{d-m}), and exchange (W_{ex}) rates of the mechanisms. 47
Table S15. Backward (Yb^{III} to ^{3}MLCT) intramolecular energy transfer rates (W, s^{-1}) calculated for the **YbRu-3** complex. Δ (cm^{-1}) is the donor-acceptor energy difference, W(%) is the percentage of the pathway, W_{total} is the sum of the dipole-dipole (W_{d-d}), dipole-multipole (W_{d-m}), and exchange (W_{ex}) rates of the mechanisms. 47

Reagents and methods

Trityl chloride (TrtCl), thioacetic acid (SHAcOH), 5-amino-1,10-phenantroline ($PhenNH_2$), 3-(dimethylamino-propyl)-ethyl-carbodiimide hydrochloride (EDC·HCl), 4-(dimethylamino)pyridine (DMAP), 5-amino-isophtalic acid, maleic anhydride, sodium acetate ($AcONa \cdot 3H_2O$), acetic anhydride (Ac_2O), trifluoroacetic acid (TFA), 1,4-diazabicyclo[2.2.2]octane (DABCO), triisopropylsilane (TIS), cis-dichlorobis(bipyridine)ruthenium(II) (bpy_2RuCl_2), ammonium hexafluorophosphate (NH_4PF_6), salicylhydroxamic acid (H_3shi), sodium benzoate, gallium(III) nitrate hydrate, yttrium(III) nitrate hydrate, erbium(III) nitrate hydrate, neodymium(III) nitrate hydrate, ytterbium(III) nitrate hydrate, pyridine (Pyr), anhydrous dimethylformamide (99.8% extra dry), dimethylformamide (reagent grade) (DMF), dichloromethane (DCM), diethyl ether (Et_2O), ethyl acetate (EtOAc), n-hexane, methanol (MeOH), $CDCl_3$, dimethylsulfoxide (spectroscopic grade) (DMSO), DMSO-d_6 were purchased from commercially available sources and used without further purification.

Silica gel chromatography was performed using Silica gel, technical grade, pore size 60 Å, 70-230 mesh, 63-200 µm, purchased from Merck.

Elemental analysis (C, H, N) was performed on FLASH 1112 (ISCR) Thermo Fisher elemental analyzer by Le Centre Régional de Mesures Physiques de l'Ouest (CRMPO) at l'Université de Rennes. All samples were dried in vacuum before the analysis.

Nuclear Magnetic Resonance (NMR) measurements for organic compounds and yttrium(III) MCs were performed at 25°C on a Bruker Avance-III HD 600 spectrometer, equipped with a 5-mm BBFO Smartprobe or on a Bruker 700 MHz NMR spectrometer equipped with a 5 mm TCI cryoprobe at 25 °C

ElectroSpray Ionization – Ion Trap – Mass Spectrometry (ESI-IT-MS). DMF or MeOH solutions of MC samples were diluted at the micromolar range in a mixture of 50% acetonitrile, 49.8% water and 0.2% formic acid for the MS acquisitions in a positive ion mode or in 50% acetonitrile and 50% water for the MS acquisitions in a negative ion mode. Samples were analyzed by direct infusion in a HCT Ultra PTM Discovery mass spectrometer from Bruker Daltonics (Germany) equipped with an electrospray ion source. The nebulizer gas pressure was 11 psi and the spray voltage was 3.5kV. The drying gas flow was of 5 L/min and the temperature was 300°C. MS spectra were acquired over 50–3000 m/z range or 1000–4000 m/z range until the ion charge control target had reached 90000. The target mass was adjusted for each analyzed compound to promote its detection. The MS acquisitions were carried out in standard-enhanced scan-mode (8100 m/z units per second) for the 50–3000 m/z range and in extended scan mode (27000 m/z units per second) for the 1000–4000 m/z range. External calibration was performed with the ESI-L Low Concentration Tuning Mix (Agilent Technologies). The instrument was controlled using EsquireControl 6.2 software (Bruker Daltonics) and mass spectra were processed using DataAnalysis 4.0 SP2 software (Bruker Daltonics). For monomeric MCs, the positions of experimental peaks were compared with the theoretical monoisotopic masses. For dimeric MCs,

experimental peaks were compared to the theoretical masses calculated as an average of the isotopic distribution.

Fourier-transform infrared spectroscopy (FTIR) measurements were recorded for samples in the solid state on a Thermo Scientific Nicolet iS10 spectrometer in FT ATR mode. Data were processed using Omnic software.

Synthesis of $Na[LnGa_4(shi)_4(benzoate)_4]$ MC monomers (Ln-2, Ln = Y^{III}, Yb^{III}, Er^{III}, Nd^{III})

Synthesis of Y-2

The complex was synthesized according to a previously published procedure:[1] $Y(NO_3)_3{\cdot}6H_2O$ (0.5 g, 1.3 mmol, 1 equiv.) was dissolved in 200 mL of MeOH. After complete dissolution of the salt, $Ga(NO_3)_3{\cdot}8H_2O$ (2.087 g, 5.2 mmol, 4 equiv.) and salicylhydroxamic acid (0.8 g, 5.2 mmol, 4 equiv.) were added consequently and stirred for 1 h at room temperature. Sodium benzoate (2.257 g, 15.6 mmol, 12 equiv.) was added slowly, allowing total dissolution, and the mixture was left to stir overnight. Next day, the mixture was filtered, and pyridine (10 mL) was added to the filtrate, followed by stirring for 15 min at room temperature. The mixture was filtered again, and the filtrate was allowed to slowly evaporate, resulting in the precipitation of **Y-2** as white crystals, which were collected by filtration, washed with MeOH, air-dried and isolated as $Na[YGa_4(shi)_4(benzoate)_4]{\cdot}2Pyr$ (0.660 g, 0.45 mmol, 34% yield).

^{1}H-NMR (600 MHz, DMSO-d_6, Figure S1): δ 8.60 (d, J = 4.02 Hz, 6H, Pyr (H2, H4)), 7.94 (m, 8H, benzoate (H4, H6)), 7.87 (m, 4H, shi^{3-} (H3)), 7.79 (m, 3H, Pyr (H6)), 7.40 (t, J = 7.6 Hz, 4H, benzoate (H1)), 7.45 (t, J = 7.4 Hz, 4H, benzoate (H3)), 7.39 (m, 6H, Pyr (H1, H5)), 7.35 (t, J = 7.7 Hz, 4H, benzoate (H2)), 7.23 (t, J = 7.4 Hz, 4H, shi^{3-} (H2)), 6.87 (d, J = 8.2 Hz, 4H, shi^{3-} (H6)), 6.71 (t, J = 7.5 Hz, 4H, shi^{3-} (H1)) ppm.

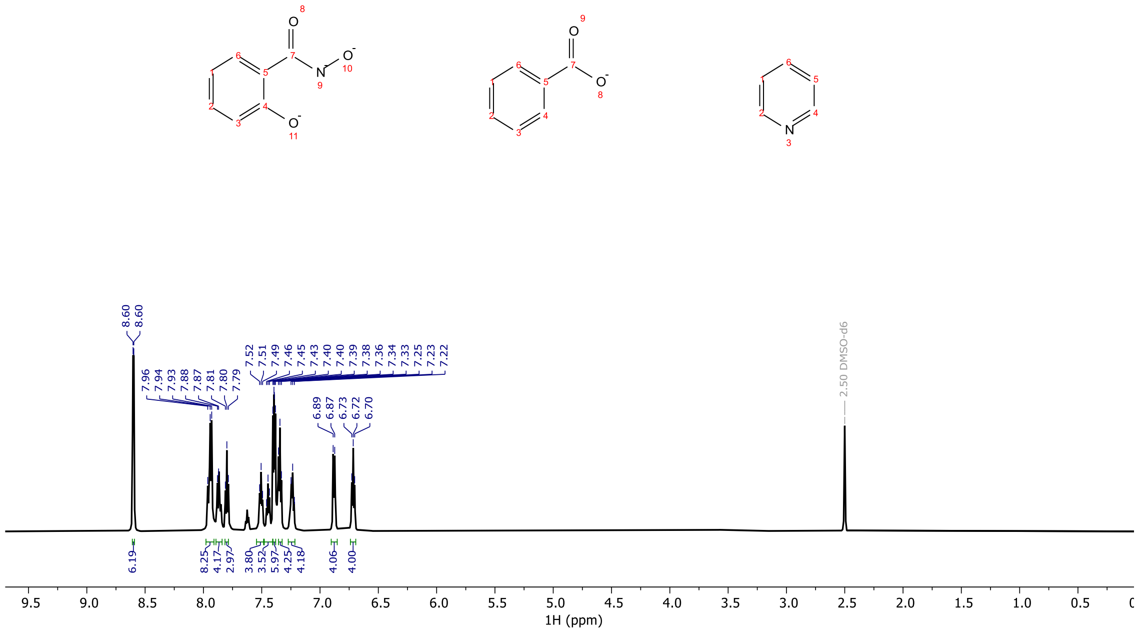

*Figure S1. ¹H-NMR spectrum of **Y-2** (DMSO-d$_6$, 600 MHz).*

ESI-MS (Figure S2), m/z: $[YGa_4(shi)_4(C_7H_5O_2)_4]^-$ calcd. for $C_{56}H_{36}Ga_4N_4O_{20}Y$ 1448.8; found 1448.9.

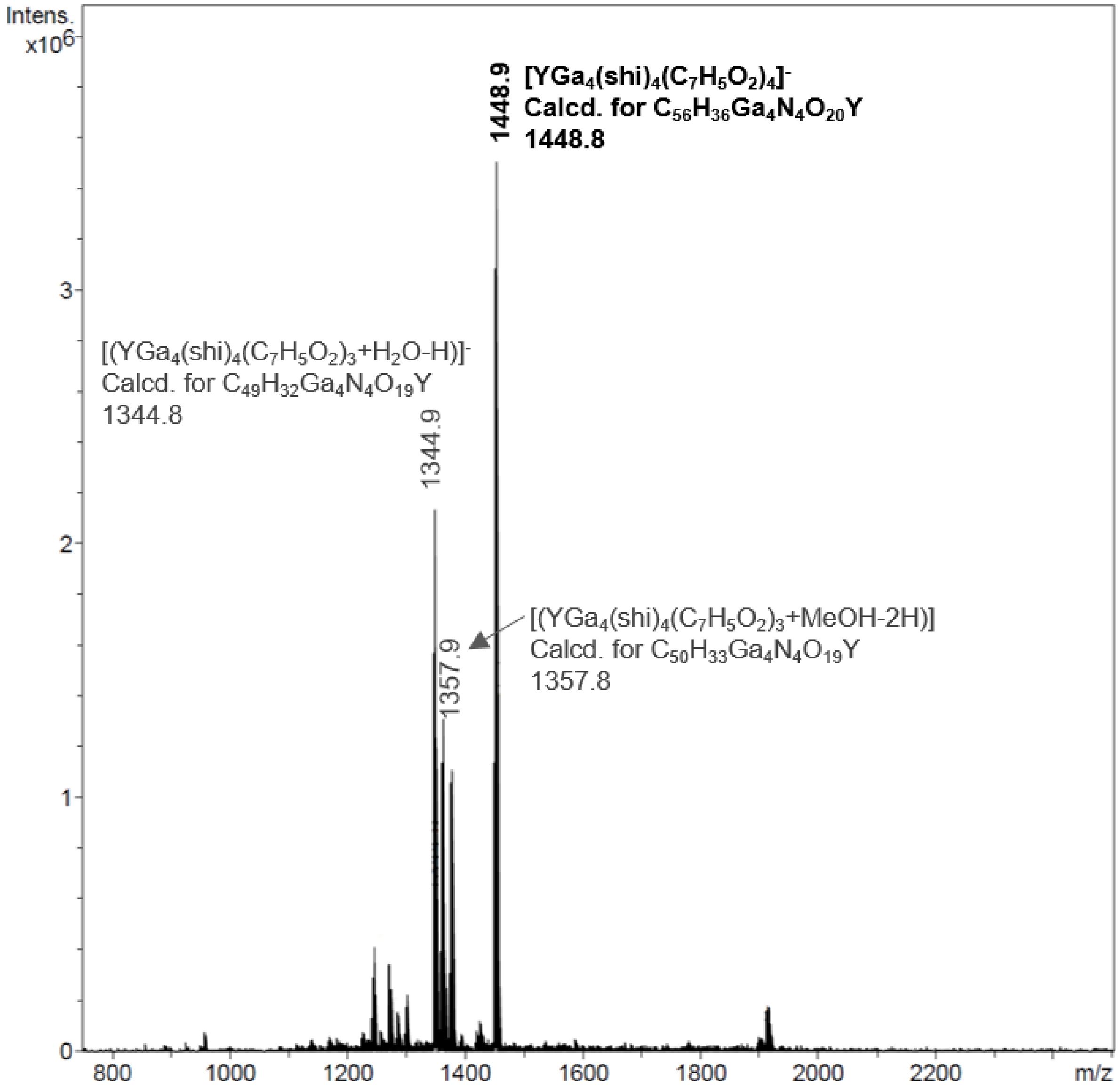

*Figure S2. ESI-MS spectrum of **Y-2** in MeOH (negative mode).*

Synthesis of **Ln-2** (Ln = Yb^{III}, Er^{III}, Nd^{III})

Yb^{III}, Er^{III} and Nd^{III} analogues of **Ln-2** were synthesized following the same synthetic pathway as for **Y-2** by replacing $Y(NO_3)_3{\cdot}6H_2O$ with the corresponding Ln^{III} nitrates: $Yb(NO_3)_3{\cdot}5H_2O$, $Er(NO_3)_3{\cdot}5H_2O$, $Nd(NO_3)_3{\cdot}6H_2O$.

Yb-2

White amorphous solid; isolated yield: 0.805 g, 0.525 mmol, 42%.

ESI-MS (Figure S3), m/z: $[YbGa_4(shi)_4(C_7H_5O_2)_4]^-$ calcd. for $C_{56}H_{36}Ga_4N_4O_{20}Yb$ 1533.8; found 1533.9.

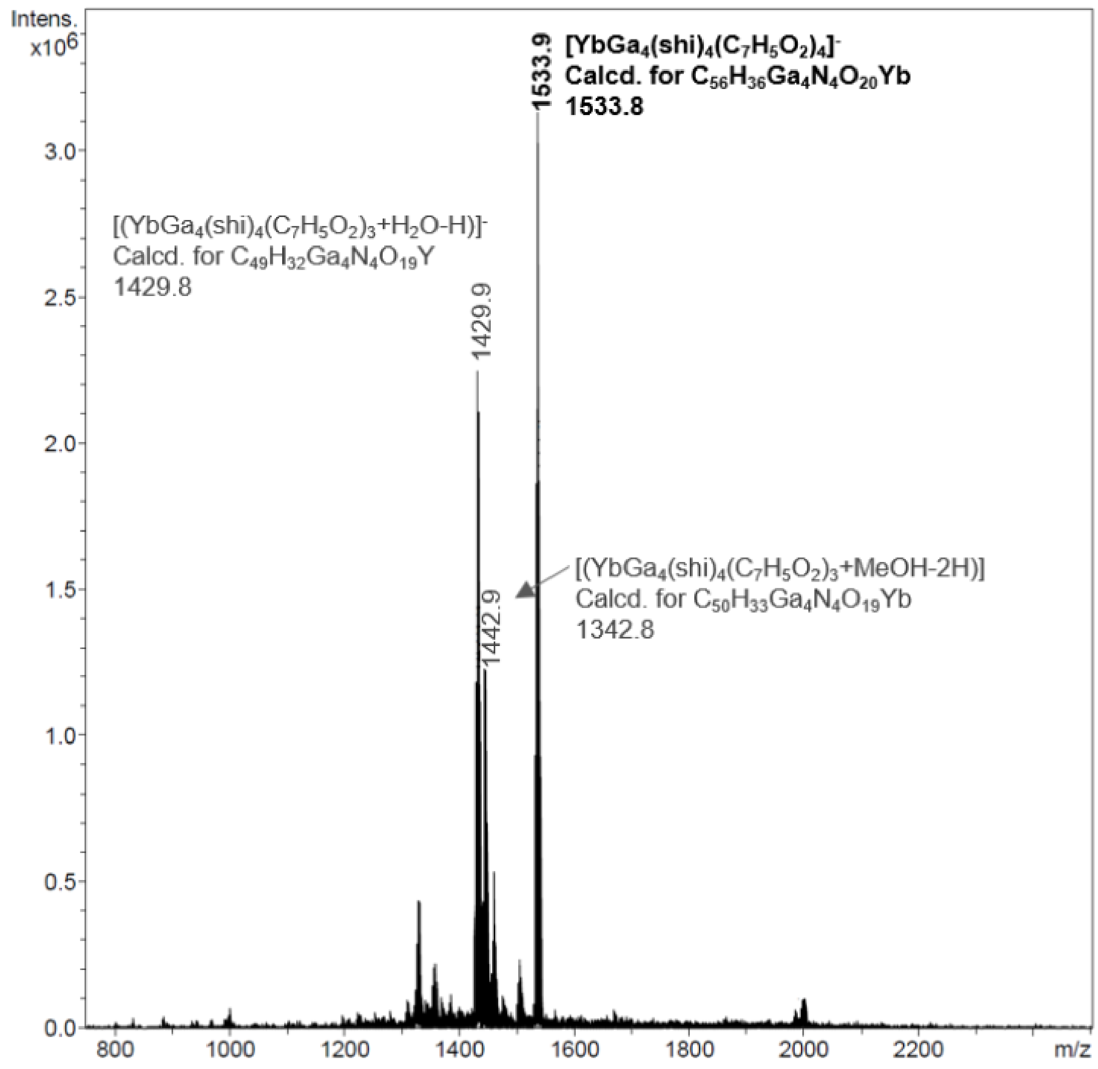

*Figure S3. ESI-MS spectrum of **Yb-2** (negative mode).*

Er-2

Pink solid, isolated yield: 0.288 g, 0.188 mmol, 15%.

ESI-MS (Figure S4), m/z: [ErGa$_4$(shi)$_4$(C$_7$H$_5$O$_2$)$_4$]$^-$ calcd. for $C_{56}H_{36}Ga_4N_4O_{20}Er$ 1531.8; found 1531.9.

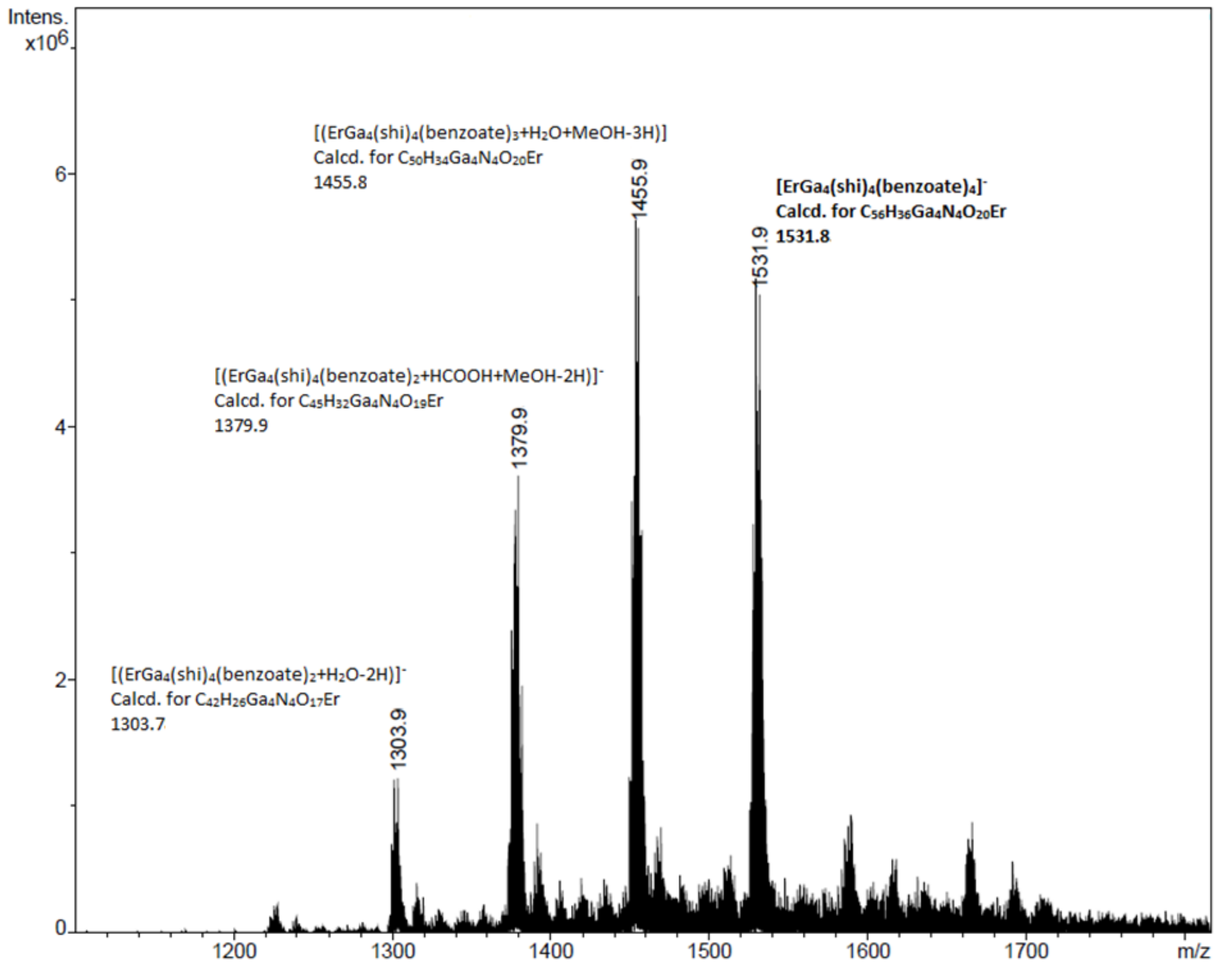

*Figure S4. ESI-MS spectrum of **Er-2** (negative mode).*

Nd-2

Off-white solid; isolated yield: 0.334 g, 0.225 mmol, 18%.

ESI-MS (Figure S5), m/z: $[NdGa_4(shi)_4(C_7H_5O_2)_4]^-$ calcd. for $C_{56}H_{36}Ga_4N_4O_{20}Nd$ 1507.8; found 1507.9.

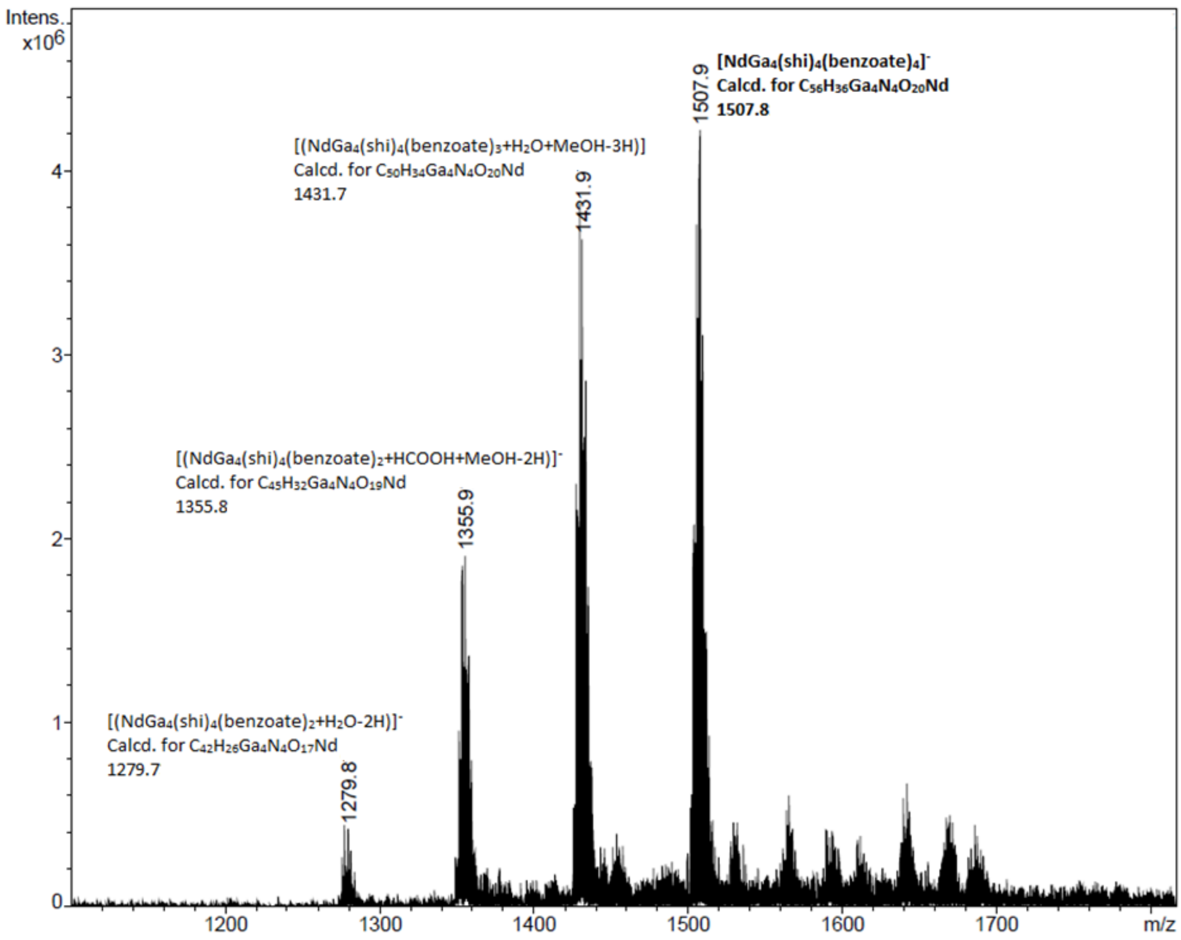

*Figure S5. ESI-MS spectrum of **Nd-2** (negative mode).*

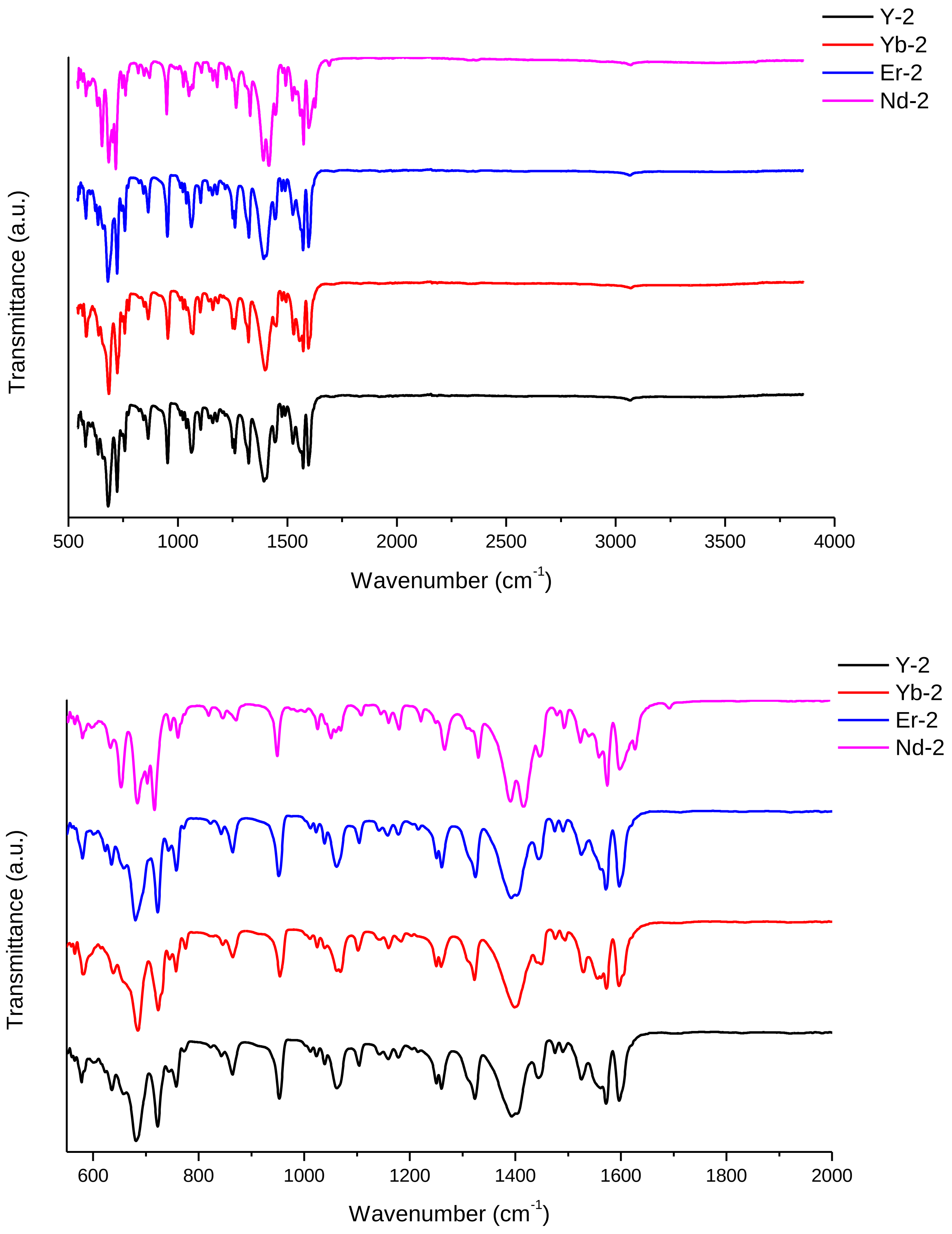

*Figure S6. FTIR spectra of **Ln-2** (Ln = Y^{III}, Yb^{III}, Er^{III}, Nd^{III}).*

Synthesis of [bpy$_2$RuPhenMipH$_2$][PF$_6$]$_2$ (Ru-1)

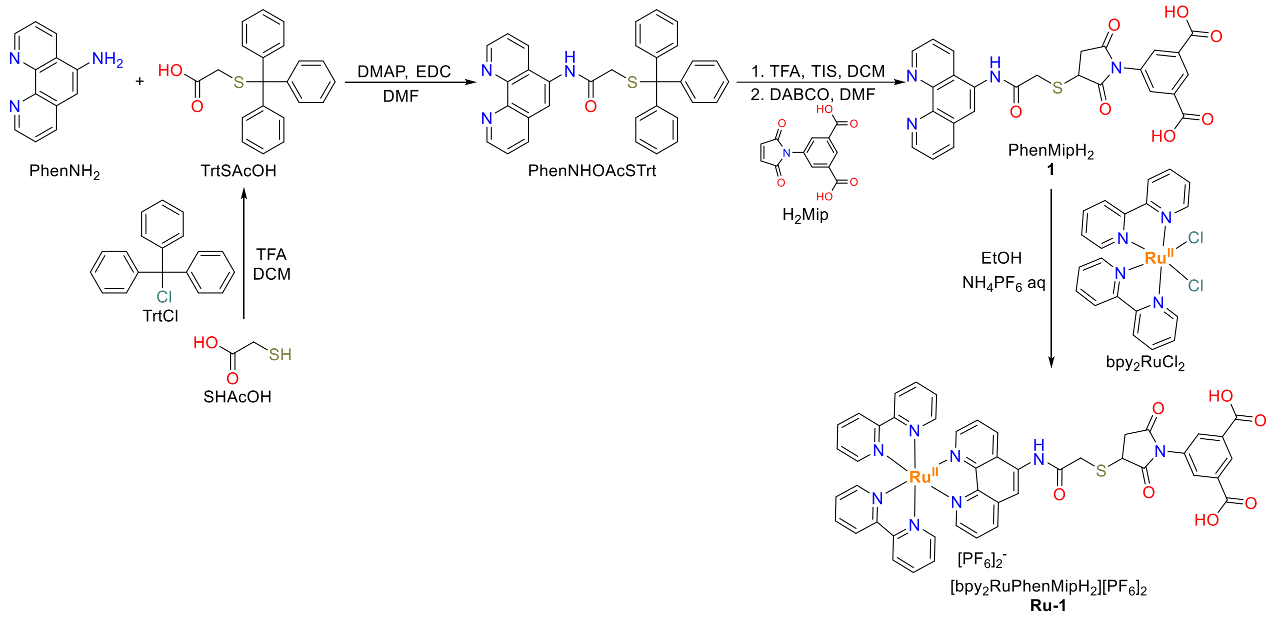

*Scheme S1. Synthetic pathway for the preparation of **Ru-1**.*

Synthesis of 2-(tritylthio)acetic acid (TrtSAcOH)

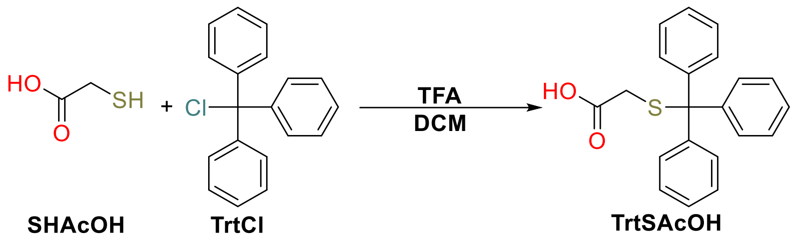

Scheme S2. Synthesis of the TrtSAcOH intermediate.

The compound was synthesized according to a modified procedure previously published:[2] trityl chloride (TrtCl, 15.83 g, 56.8 mmol, 1 equiv.) and thioacetic acid (SHAcOH, 5.5 g, 59.6 mmol, 1.05 equiv.) were stirred overnight at RT in 150 mL of DCM. Next day, DCM was evaporated under vacuum, resulting in a yellow oil. 100 mL of n-hexane were added over the oily mixture and stirred for 1 h at RT, leading to the formation of a white precipitate. The precipitate (TrtSAcOH) was filtered and washed with n-hexane (2×50 mL), then dried under reduced pressure. Yield: 17.8 g (94%).

^{1}H-NMR (600 MHz, $CDCl_3$, Figure S7): δ 7.42 (d, J = 7.4 Hz, 6H, H10, H14, H16, H19, H20, H24), 7.30 (t, J = 7.7 Hz, 6H, H11, H13, H16, H18, H21, H23), 7.23 (t, J = 6.5 Hz, 3H, H12, H17, H22), 3.04 (s, 2H, H2) ppm.

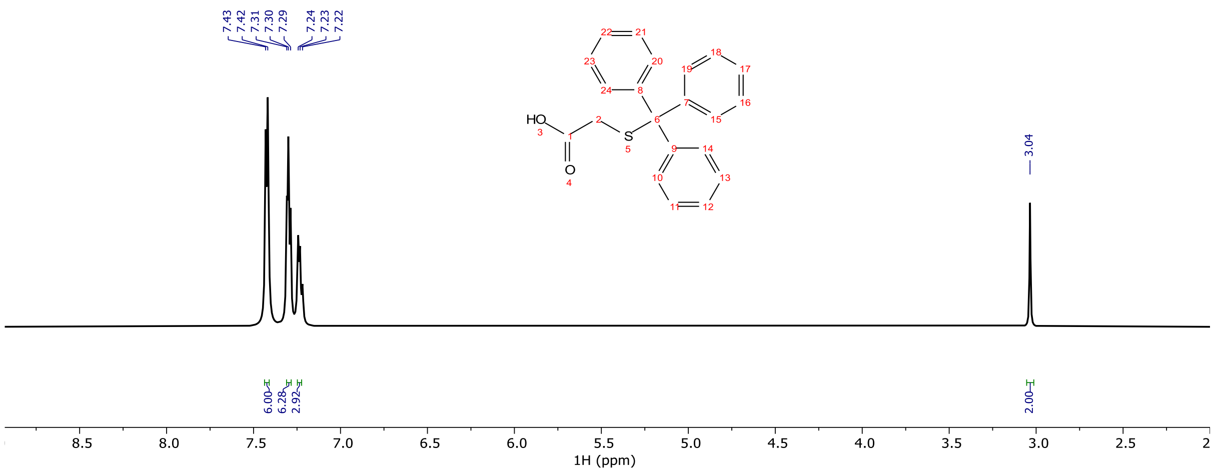

Figure S7. ^{1}H-NMR spectrum of TrtSAcOH ($CDCl_3$, 600 MHz).

Synthesis of *N*-(1,10-phenantrolin-5-yl)-2-(trytilthio)acetamide (PhenNHOAcSTrt)

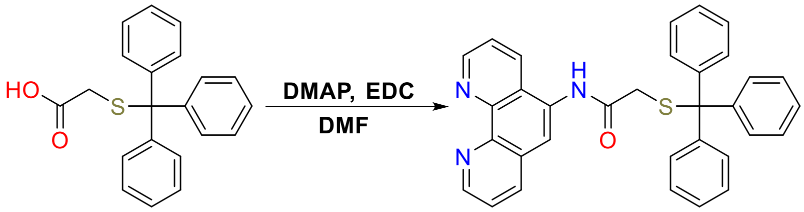

Scheme S3. Synthesis of the PhenNHOAcSTrt intermediate.

The compound was synthesized according to a modified reported procedure:[3] the 5-amino-1,10-phenantroline ($PhenNH_2$, 0.5g, 2.56 mmol, 1 equiv.) was dissolved in 20 mL of anhydrous DMF. On this solution, TrtSAcOH (0.856g, 2.56 mmol, 1 equiv.), EDC·HCl (1.473g, 7.68 mmol, 3.5 equiv.) and DMAP (0.469g, 3.84 mmol, 1.5 equiv.) were added and the resulting mixture was stirred overnight at RT. Next day, DMF was evaporated under reduced pressure and the compound was dissolved in DCM (50mL), then washed with a saturated aqueous $NaHCO_3$ solution (3×50 mL) and dried. The solid obtained was purified by column chromatography (silica, DCM:MeOH = 95:5), affording 0.605g (yield = 46%) of a beige solid.

^{1}H-NMR (600 MHz, $CDCl_3$, Figure S8): δ 9.20 (dd, 1H, J_1 = 1.6Hz, J_2 = 2.8 Hz, 1H, H36), 9.10 (dd, 1H, J_1 = 1.7Hz, J_2 = 2.5 Hz, 1H, H33), 8.57 (s, 1H, H3), 8.20 (s, 1H, H26), 8.17 (dd, J_1 = 1.7 Hz, J_2 = 6.4 Hz, 1H, H31), 7.95 (dd, J_1 = 1.6 Hz, J_2 = 6.8 Hz, 1H, H37), 7.64-7.62 (m, 1H, H38), 7.61-7.59 (m, 1H, H32), 7.50 (d, J = 7.3 Hz, 6H, H10, H14, H15, H19, H20, H24), 7.26 (t, J = 7.8 Hz, 6H, H11, H13, H16, H18, H21, H23), 7.15 (t, J = 7.3 Hz, 3H, H12, H17, H22), 3.56 (s, 2H, H2) ppm.

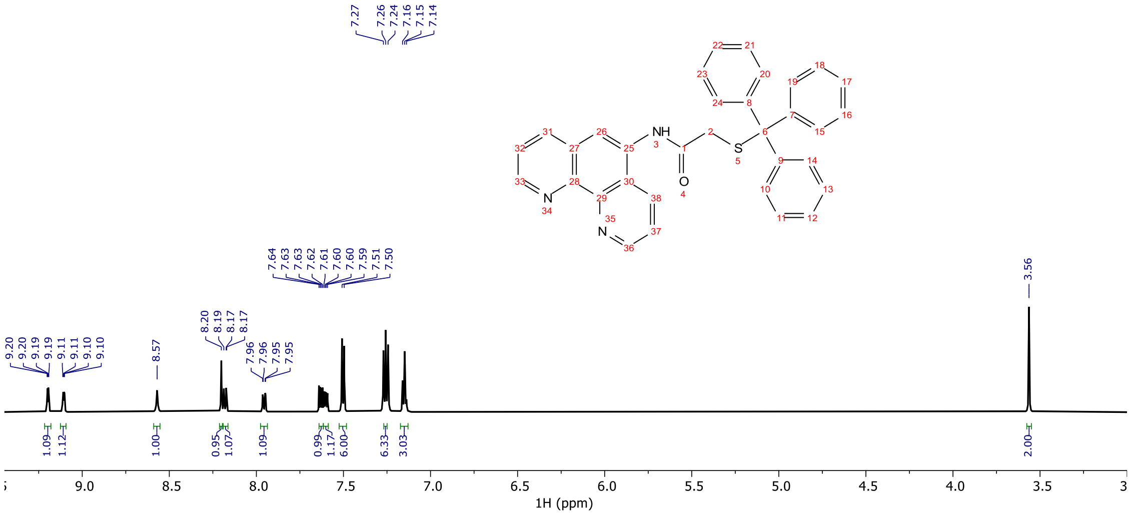

Figure S8. ^{1}H-NMR spectrum of PhenNHOAcSTrt ($CDCl_3$, 600 MHz).

Synthesis of 5-maleimide isophthalic acid (H_2Mip)

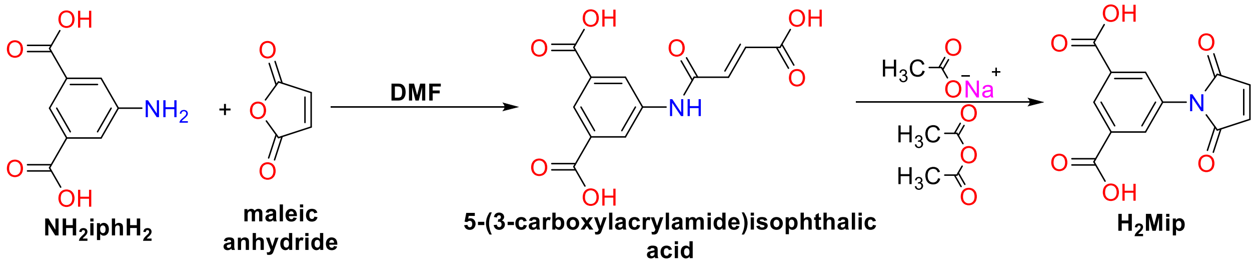

Scheme S4. Synthesis of H_2Mip.

The compound was synthesized according to a previously published procedure:[4] 5-aminoisophthalic acid (NH_2iphH_2, 18.115 g, 100 mmol, 1 equiv.) and maleic anhydride (10.787 g, 110 mmol, 1.1 equiv.) were dissolved in 200 mL of DMF and stirred overnight at RT. Next day, DMF was evaporated under reduced pressure and the solid obtained was washed with acetone. 18.92 g (yield = 68%) of 5-(3-carboxylacrylamide)isophthalic acid were isolated. In a second step, the obtained 5-(3-carboxylacrylamide)isophthalic acid (5.584 g, 20 mmol, 1 equiv.), $AcONa{\cdot}3H_2O$ (1.361 g, 10 mmol, 0.5 equiv.) and 30 mL of acetic anhydride were stirred at reflux for 2.5 h. The acetic anhydride was removed under reduced pressure and 50 mL of H_2O were added, allowing the slurry to stir for 2 h at 70 °C. The mixture was cooled to RT, filtered and washed with H_2O (3×20mL), affording 3.8 g (yield = 73%) of H_2Mip.

^{1}H-NMR (600 MHz, DMSO-*d*$_6$, Figure S9): δ 8.45 (s, 1H, H6), 8.18 (d, *J* = 1.6 Hz, 2H, H1, H3), 7.22 (s, 2H, H15, H16) ppm.

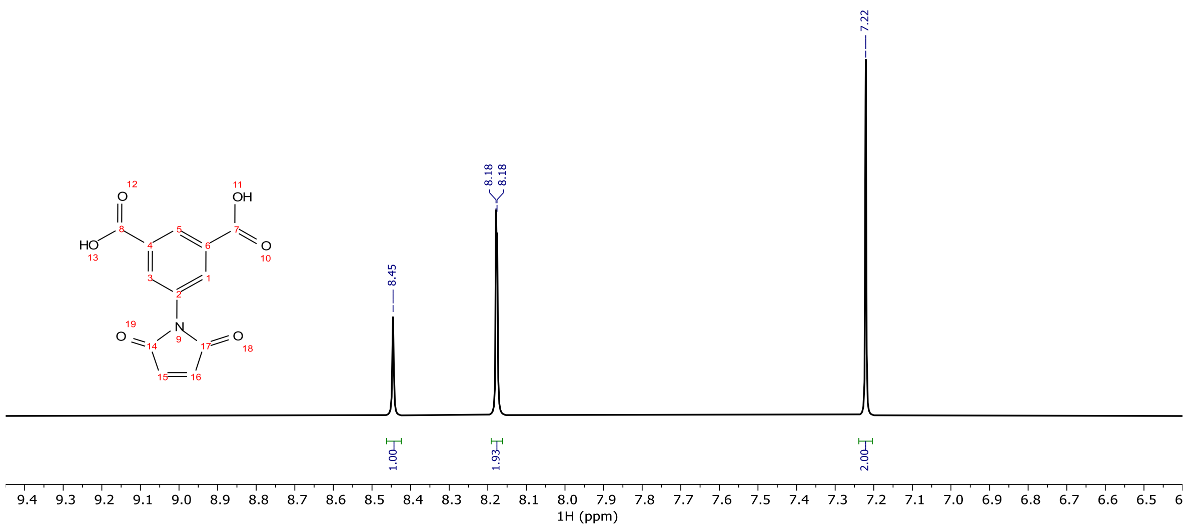

Figure S9. ^{1}H-NMR spectrum of H_2Mip (DMSO-d_6, 600 MHz).

Synthesis of PhenMipH_2 (**1**)

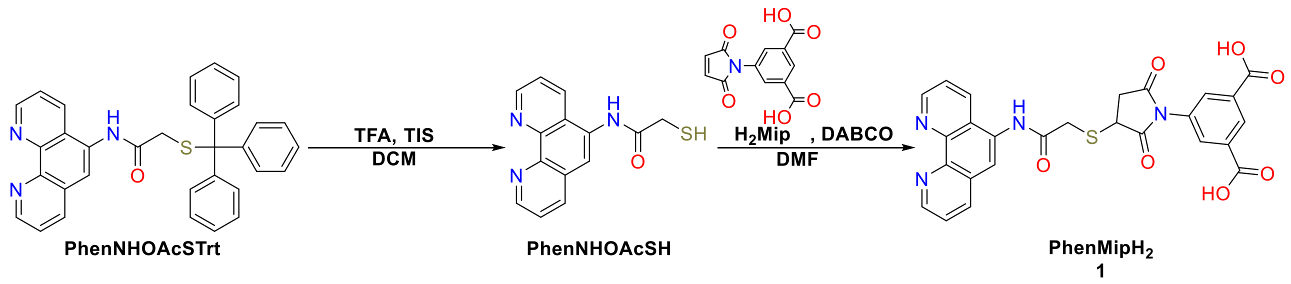

*Scheme S5. Synthesis of **1**.*

The synthetic procedure comprises two steps. In the first step, the trityl group was cleaved following an adapted synthetic method:[5] 320 mg of PhenNHOAcSTrt (0.625 mmol) were stirred in a mixture of 15 mL of DCM + 1% (v/v) TIS + 20% (v/v) TFA for 2 h, at RT until TLC (silica, DCM:MeOH = 95:5) indicated the full conversion of the starting material. The solvent was removed completely under reduced pressure and the compound obtained (PhenNHOAcSH) was used immediately in the following step, without further purification, considering the yield as being quantitative. The second step of the reaction was performed according to an adapted Michael addition procedure:[6] PhenNHOAcSH (168.63 mg, 0.625 mmol, 1 equiv.), H_2Mip (163.25 mg, 0.625 mmol, 1 equiv.) and 1,4-diazabicyclo[2.2.2]octane (DABCO, 7.01 mg, 0.063 mmol, 0.1 equiv.) were dissolved in a minimal amount of DMF (5 mL) and stirred overnight at RT. The next day, DMF was removed completely under reduced pressure, followed by the addition of 10 mL of H_2O. The resulting yellow precipitate was filtered and washed with H_2O (20 mL), DCM (20 mL) and then Et_2O (20 mL), in order to remove traces of cleaved trityl and free thiol. 220 mg (yield = 66%) of beige compound were isolated.

^{1}H-NMR (600 MHz, DMSO-d_6, Figure S10): δ 10.60 (s, 1H, H3), 9.23 (dd, J_1 = 1.5 Hz, J_2 = 2.9 Hz, 1H, H17), 9.13 (dd, J_1 = 1.6 Hz, J_2 = 3.1 Hz, 1H, H14), 8.87 (s, 1H, H30), 8.73 (d, J = 8.2 Hz, 1H, H19), 8.44 (s, 1H, H12), 8.36 (s, 1H, H7), 8.13 (d, J = 1.6 Hz, 2H, H28, H32), 8.02-7.98 (m, 2H, H13, H18), 4.43-4.40 (m, 2H, H2), 3.40-3.36 (m, 3H, H20, H21) ppm.

^{13}C-NMR (600 MHz, DMSO-*d*$_6$, Figure S11): δ 175.9 (C1), 174.5 (C22, C24), 171.9 (C33, C36), 170.1 (qC), 169.4 (C29, C31), 166.2 (C27), 158.3 (qC), 150.0 (C14, C17), 140.2 (C19), 133.6 (C30), 132.5 (C28, C32), 129.9 (C12), 129.3 (qC), 125.4 (C18), 125.2 (C13), 119.7 (C7), 44.2 (C2), 36.1 (C21), 34.8 (C20) ppm.

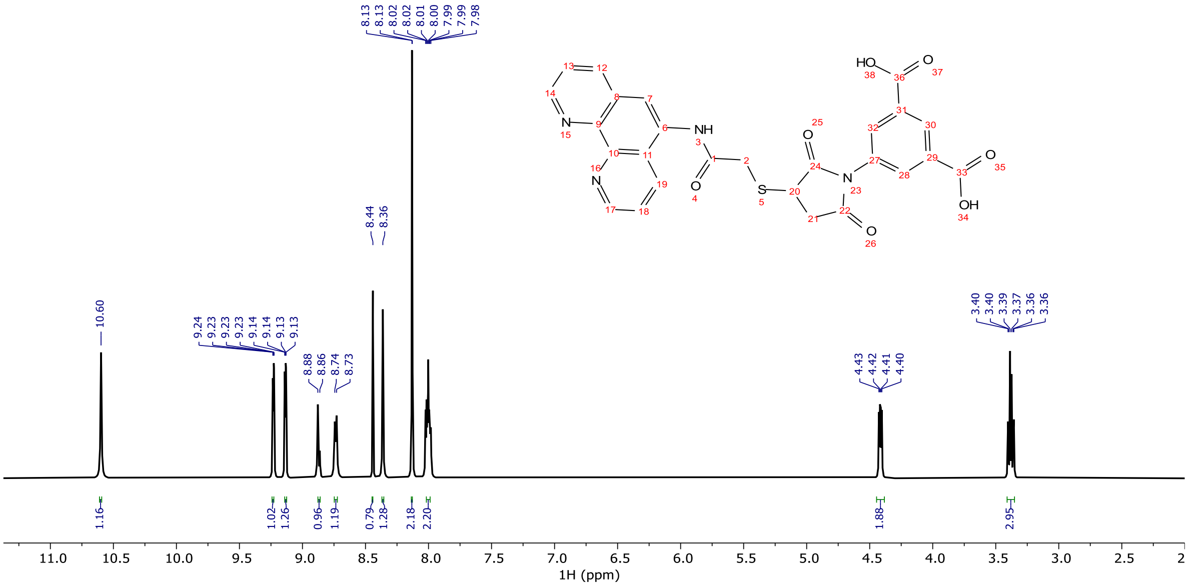

*Figure S10. ^{1}H-NMR spectrum of **1** (DMSO-d$_6$, 600 MHz).*

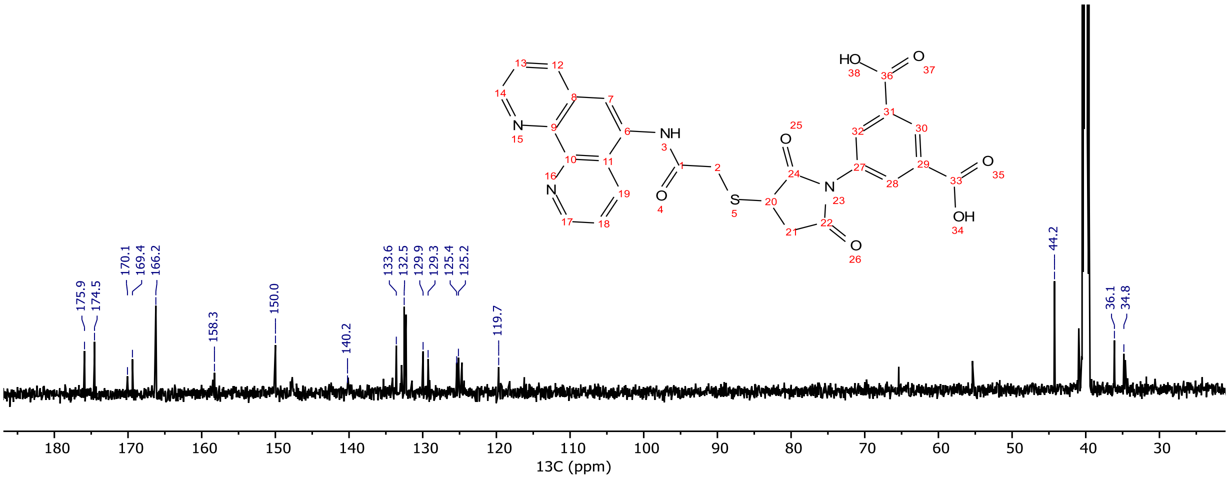

*Figure S11. ^{13}C-NMR spectrum of **1** (DMSO-d$_6$, 600 MHz).*

Synthesis of **Ru-1**

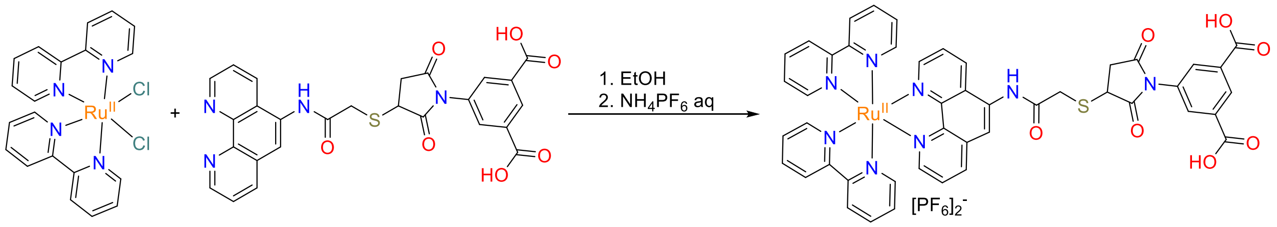

bpy$_2$RuCl$_2$ **PhenMipH$_2$ 1** **[bpy$_2$RuPhenMipH$_2$][PF$_6$]$_2$ Ru-1**

*Scheme S6. Synthesis of **Ru-1**.*

The complex **Ru-1** was synthesized according to a standard Ru^{II} complexation procedure:[7] **1** (200mg, 0.37 mmol, 1 equiv.) and *cis*-dichlorobis(bipyridine)ruthenium(II) (bpy$_2$RuCl$_2$, 170 mg, 0.37 mmol, 1 equiv.)

were dissolved in 20 mL of EtOH and refluxed for 4h under N_2. The solution was filtered while hot and a saturated solution of aqueous NH_4PF_6 was added dropwise allowing an orange precipitate to be formed. The precipitate was filtered and washed with 30 mL of H_2O and 30 mL of Et_2O, yielding 227 mg yield = 50%) of **Ru-1**.

^{1}H-NMR (600 MHz, DMSO-*d*$_6$, Figure S12): δ 10.76 (s, 1H, **1** (H15)), 8.95 (t, *J* = 9.6Hz, 1H, **1** (H1)), 8.87(d, *J* = 8.4Hz, 2H, bpy (H4)), 8.83 (d, *J* = 8.4Hz, 2H, bpy (H11)), 8.69 (d, *J* = 7.8Hz, 1H, **1** (H12)), 8.63 (splitted signal, 1H, **1** (H9)), 8.46 (ds, 1H, **1** (H30)), 8.21 (t, *J* = 7.8Hz, bpy (H5)), 8.17-8.15 (m, 1H, **1** (H5)), 8.13-8.07 (m, 4H, **1** (H28, H32), bpy (H10)), 8.04-8.02 (m, 1H, **1** (H14)), 7.93-7.89 (m, 1H, **1** (H6)), 7.84-7.82 (m, 1H, **1** (H13)), 7.82-7.80 (m, 1H, bpy (H8)), 7.61-7.56 (m, 4H, bpy (H6, H9)), 7.41-7.35 (m, 3H, bpy (H1, H8)), 4.41-4.39 (m, 1H, **1** (H20)), 4.00 (splitted signal, 1H, **1** (H21)), 3.89 (splitted signal, 1H, **1** (H21)), 2.99-2.95 (m, 2H, **1** (H17)) ppm.

^{13}C-NMR (600 MHz, DMSO-*d*$_6$, Figure S13): δ 175.9 (**1** (C16)), 174.6 (**1** (C22, C24)), 171.9 (**1** (C33, C36)), 169.5 (**1** (C29, C31)), 166.5 (**1** (C27)), 157.3 (bpy (C2, C7)), 152.9 (**1** (C5)), 151.9 (**1** (C13), bpy (C6, C9)), 151.4 (**1** (C14)), 147.9 (**1** (qC)), 145.8 (**1** (qC)), 145.1 (**1** (qC)), 138.4 (bpy (C5)), 138.3 (**1** (C28, C32)), 136.8 (**1** (C12)), 133.3 (**1** (C1)), 130.6 (**1** (qC)), 130.1 (**1** (C30)), 128.3 (bpy (C1, C8)), 127.0 (bpy (C10)), 126.5 **1** (qC)), 126.1 (**1** (C6)), 124.9 (bpy (C4, C11)), 119.9 (**1** (C9)), 36.1 (**1** (C17)), 34.4 (**1** (C20)), 34.3 (**1** (C21)) ppm.

ESI-MS (Figure S14), m/z: [bpy_2RuPhenMipH$_2$]$^{2+}$ calcd. for $C_{46}H_{34}N_8O_7SRu$ 472.0, found 472.0; [bpy_2RuPhenMipH$_2$-H]$^+$ calcd. for $C_{46}H_{33}N_8O_7SRu$ 943.1, found 943.2; {[bpy_2RuPhenMipH$_2$][PF_6]}$^+$ calcd. for $C_{46}H_{34}N_8O_7SPF_6Ru$ 1089.1, found 1089.1; [bpy_2RuPhenMipH$_2$-3H]$^-$ calcd. for $C_{46}H_{31}N_8O_7SRu$ 941.1, found 941.1.

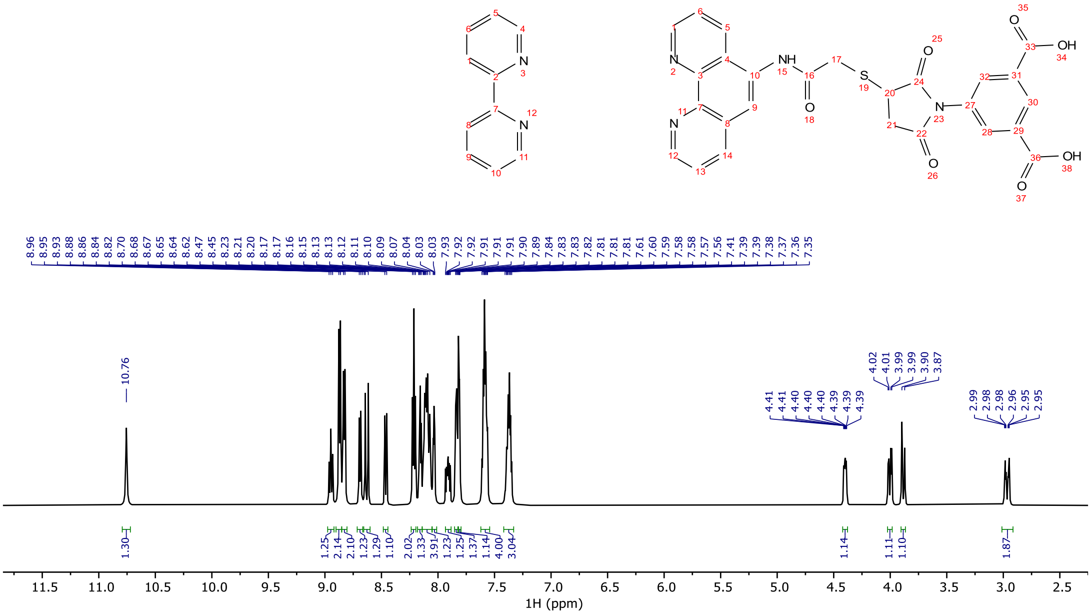

*Figure S12. ^{1}H-NMR spectrum of **Ru-1** (DMSO-d$_6$, 600 MHz).*

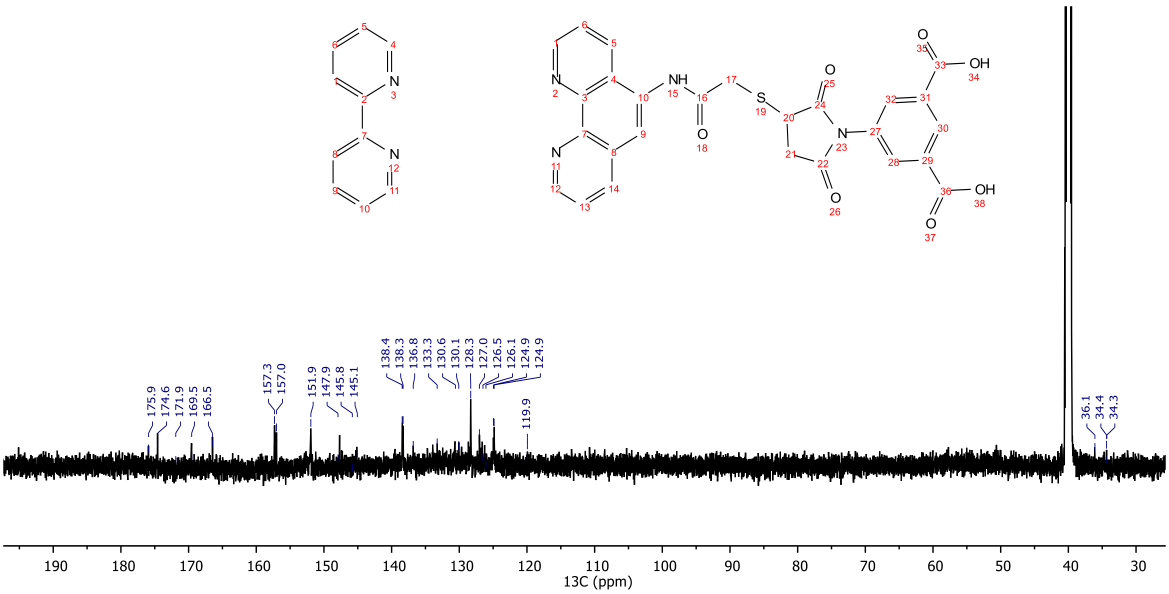

Figure S13. ^{13}C-NMR spectrum of **Ru-1** (DMSO-d_6, 600 MHz).

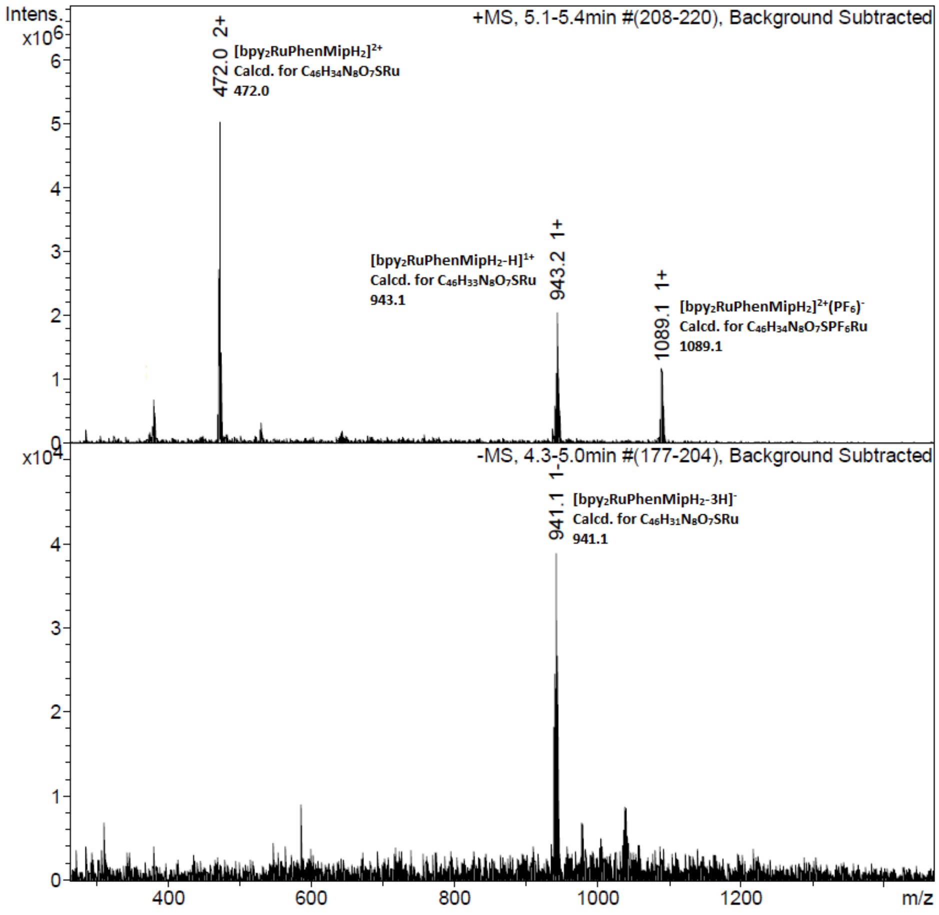

Figure S14. ESI-MS spectrum of **Ru-1**.

Synthesis of $[Ln_2Ga_8(shi)_8(bpy_2RuPhenMip)_4][PF_6]_6$ (LnRu-3, Ln = Y^{III}, Yb^{III}, Er^{III}, Nd^{III})

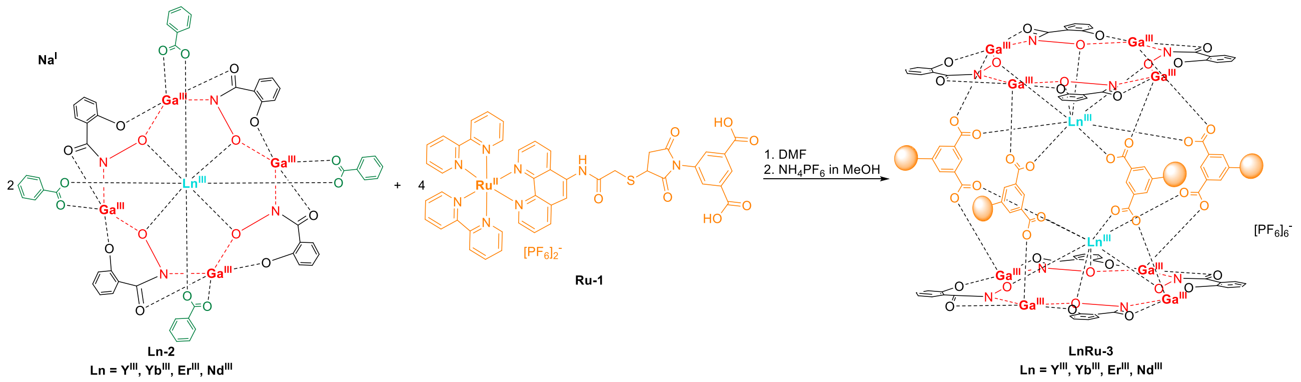

*Scheme S7. Synthetic pathway for the synthesis of **LnRu-3**.*

Complexes **LnRu-3** were synthesized according to a modified procedure:[8] The corresponding **Ln-2** (Ln = Y^{III}, Yb^{III}, Er^{III}, Nd^{III}) (2 equiv.) and **Ru-1** (4 equiv.) were dissolved in a minimum quantity of DMF (approx. 1 mL/100mg of MC monomer) and stirred overnight at RT. Next day, a saturated methanolic solution of ammonium hexafluorophosphate (NH_4PF_6) was added dropwise to the mixture, allowing the formation of an orange precipitate. This precipitate was filtered and washed with a combination of organic solvents (50 mL of MeOH and 50 mL of DCM) in order to remove traces of unreacted starting materials, then dried in air, yielding the desired **LnRu-3** compounds.

YRu-3

Isolated as $[Y_2Ga_8(shi)_8(bpy_2RuPhenMip)_4][PF_6]_6\cdot 17.9H_2O\cdot 2DCM$ (7066.6 g/mol) (43 mg, 0.006 mmol, 50% yield).

Elemental analysis calcd. for $C_{240}H_{160}N_{40}O_{52}S_4P_6F_{36}Ga_8Ru_4Y_2\cdot 2CH_2Cl_2\cdot 17.9H_2O$, %: C, 41.13; H, 2.85; N, 7.93; S, 1.81. Found C, 41.23; H, 2.47; N, 7.55; S, 1.68.

^{1}H-NMR (700 MHz, DMSO-d_6, Figure S15): δ 8.91-8.80 (m, 6H, Ru-1 (H1, H12), bpy (H4, H11)), 8.59-8.54 (bs, Ru-1 (H9)), 8.21 (bs, 2H, bpy (H5)), 8.15-8.02 (m, 7H, Ru-1 (H5, H14, H28, H30, H32), bpy (H10)), 7.89 (bs, 1H, Ru-1 (H6)), 7.81-7.74 (m, 5H, Ru-1 (H13), shi (H3), bpy (H10)), 7.57-7.49 (m, 6H, bpy (H1, H6, H9), 7.33-7.24 (m, 2H, shi (H2)), 7.14-7.08 (m, 2H, shi (H6)), 6.77-6.72 (m, 2H, shi (H1)), 4.25 (s, 1H, Ru-1 (H20)), 3.91-3.83 (bs, 2H, Ru-1 (H21)) ppm.

^{13}C-NMR (700 MHz, DMSO-d_6, Figure S16): δ 175.7 (YRu-3 (C16), 174.2 (YRu-3 (C22, C24)), 171.7 (YRu-3 (C33, C36)), 169.3 (YRu-3 (C29, C31)), 165.4 (shi (C7)), 163.4 (YRu-3 (C27)), 157.3 (bpy (C2, C7)), 157.0 (shi (C4)), 152.8 (YRu-3 (C5)), 151.9 (YRu-3 (C13), bpy (C6, C9)), 150.1 (YRu-3 (C14)), 147.6 (YRu-3 (qC)), 145.1 (YRu-3 (qC)), 144.9 (YRu-3 (qC)), 138.4 (bpy (C5)), 138.3 (YRu-3 (C28, C32)), 136.6 (YRu-3 (C12)), 133.9 (YRu-3 (C1)), 132.9 (YRu-3 (qC)), 132.2 (YRu-3 (C30)), 130.6 (shi (C2)), 128.3 (shi (C1, C6), bpy (C1, C8)), 126.9 (bpy (C10)),

126.3 (YRu-3 (qC)), 126.2 (YRu-3 (C6)), 124.9 (bpy (C4, C11)), 121.8 (YRu-3 (C9)), 116.0 (shi (C3)), 115.7 (shi (C5)), 36.3 (YRu-3 (C17)), 33.0 (YRu-3 (C20)), 31.8 (YRu-3 (C21)) ppm.

ESI-MS, m/z (Figure S23): $[Y_2Ga_8(shi)_8(bpy_2RuPhenMip)_4]^{6+}$ calcd. for $C_{240}H_{160}N_{40}O_{52}S_4Ga_8Ru_4Y_2$ 950.6, found 950.2; $\{[Y_2Ga_8(shi)_8(bpy_2RuPhenMip)_4][PF_6]_3\}^{3+}$ calcd. for $C_{240}H_{160}N_{40}O_{52}S_4P_3F_{18}Ga_8Ru_4Y_2$ 2046.2, found 2045.7. Species corresponding to fragmentation: $[bpy_2RuPhenNHCOCH_2S]^+$ calcd. for $C_{34}H_{26}N_7OSRu$ 682.1, found 682.2; $[Y_2Ga_8(shi)_8(bpy_2RuPhenMip)_3(Mip)\text{-}H]^{4+}$ calcd. for $C_{206}H_{133}N_{33}O_{51}S_3Ga_8Ru_3Y_2$ 1254.7, found 1254.6; $\{[Y_2Ga_8(shi)_8(bpy_2RuPhenMip)_3(Mip)\text{-}H][PF_6]\}^{3+}$ calcd. for $C_{206}H_{133}N_{33}O_{51}S_3PF_6Ga_8Ru_3Y_2$ 1721.6, found 1721.7; $[Y_2Ga_8(shi)_8(bpy_2RuPhenMip)_2(Mip)_2\text{-}H]^{2+}$ calcd. for $C_{172}H_{107}N_{26}O_{50}S_2Ga_8Ru_2Y_2$ 2169.3, found 2169.5.

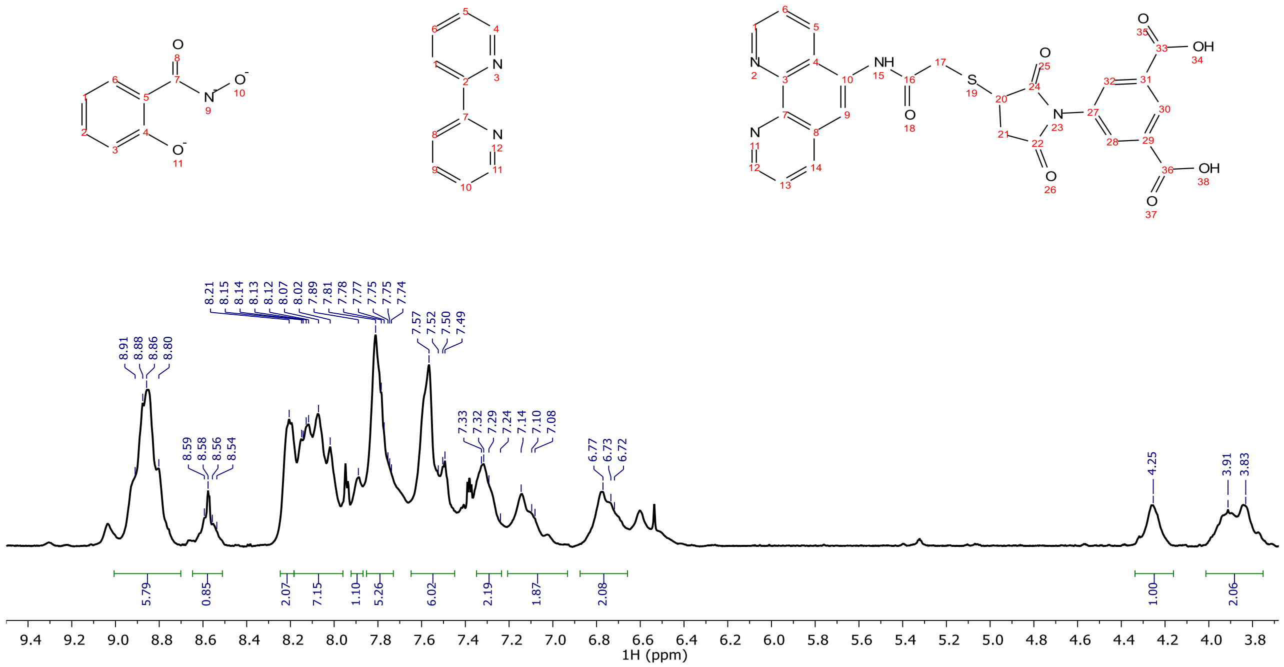

Figure S15. ^{1}*H-NMR spectrum of* ***YRu-3*** *(DMSO-d*$_6$*, 700 MHz).*

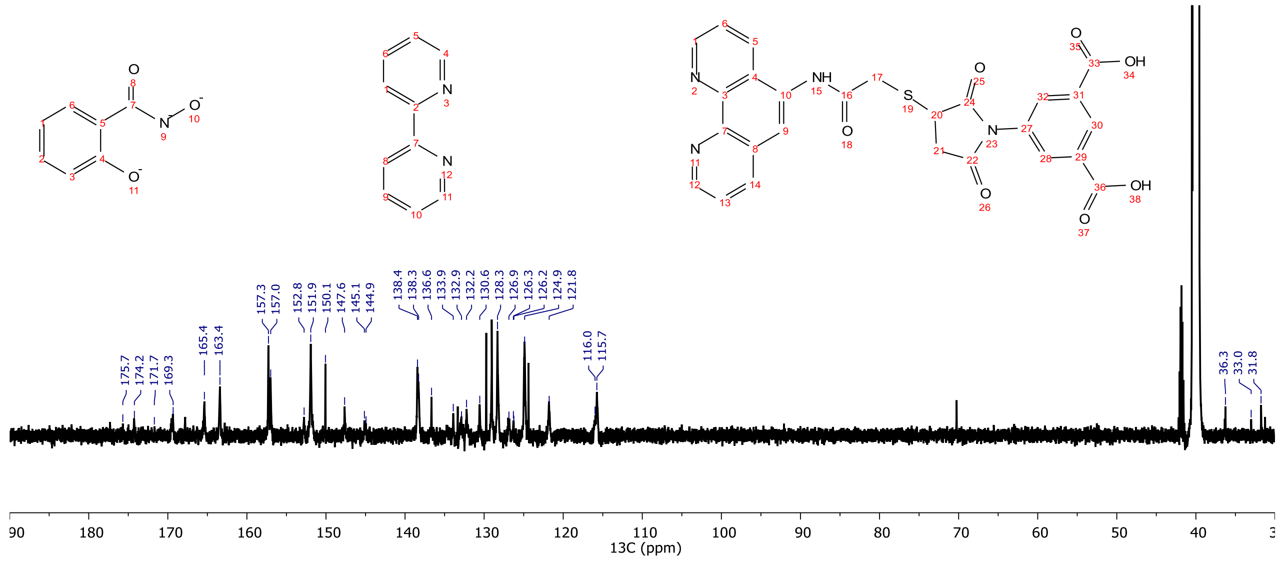

Figure S16. ^{13}*C-NMR spectrum of* ***YRu-3*** *(DMSO-d*$_6$*, 700 MHz).*

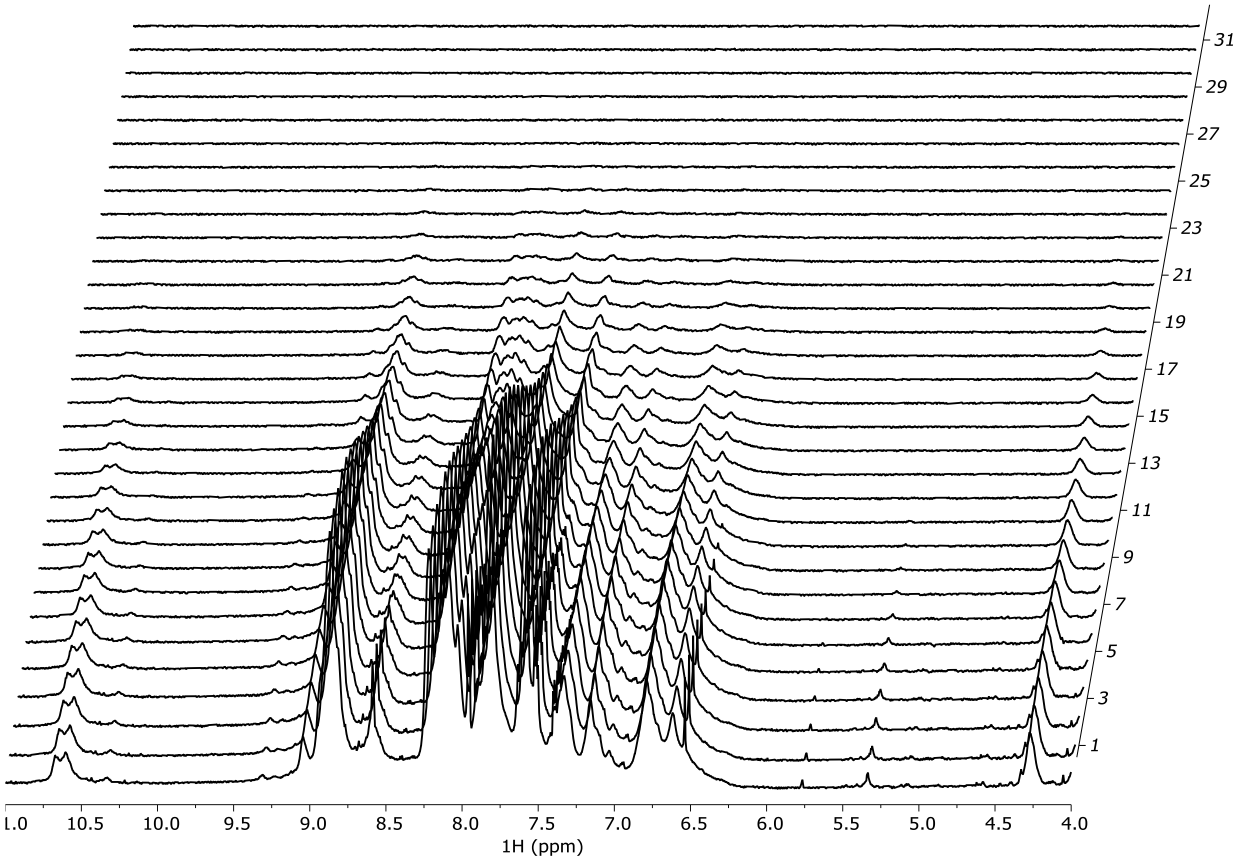

*Figure S17. Arrayed DOSY-NMR spectra of **YRu-3** (DMSO-d$_6$, 700 MHz).*

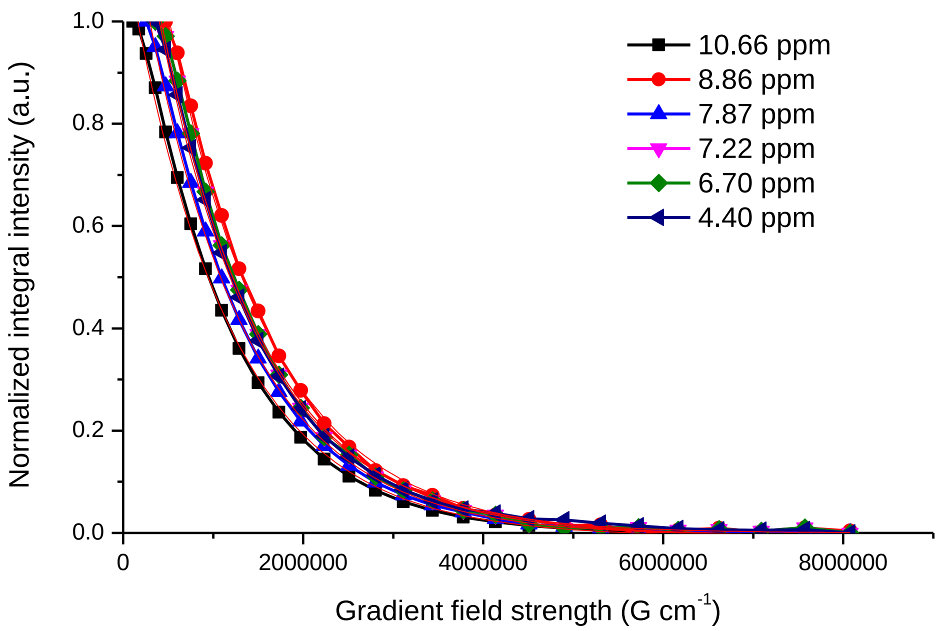

*Figure S18. Normalized integral intensity as a function of the gradient field strength (G·cm^{-1}) for **YRu-3** (DMSO-d$_6$, 700 MHz). The diffusion coefficient (D) was estimated using monoexponential decay fitting ($R^2 > 0.997$) and found to be 0.9·10^{-10} m^{2}s^{-1}.*

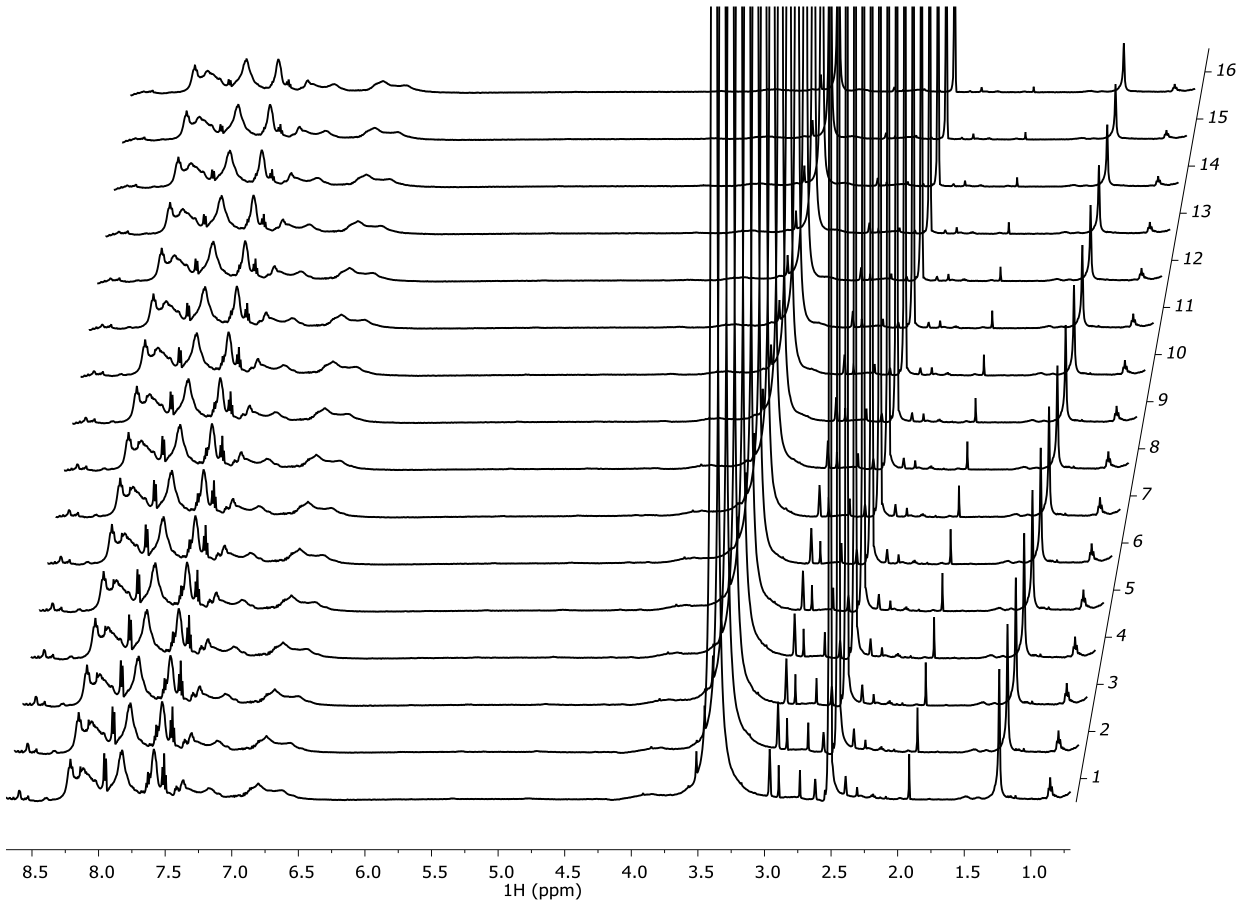

*Figure S19. Arrayed DOSY-NMR spectra of **YRu-3** (DMSO-d_6, 600 MHz) after 3 months of storage in solution.*

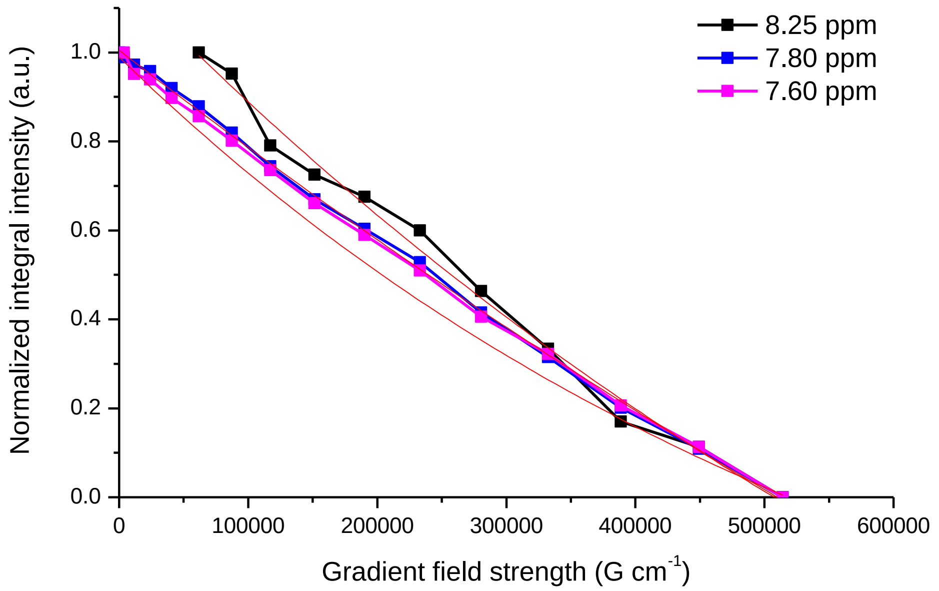

*Figure S20. Normalized integral intensity as a function of the gradient field strength (G·cm^{-1}) for **YRu-3** after 3 months of storage in solution (DMSO-d_6, 600 MHz). The diffusion coefficient (D) was estimated using monoexponential decay fitting (R^2 > 0.993) and found to be $0.9 \cdot 10^{-10}$ m^2s^{-1}.*

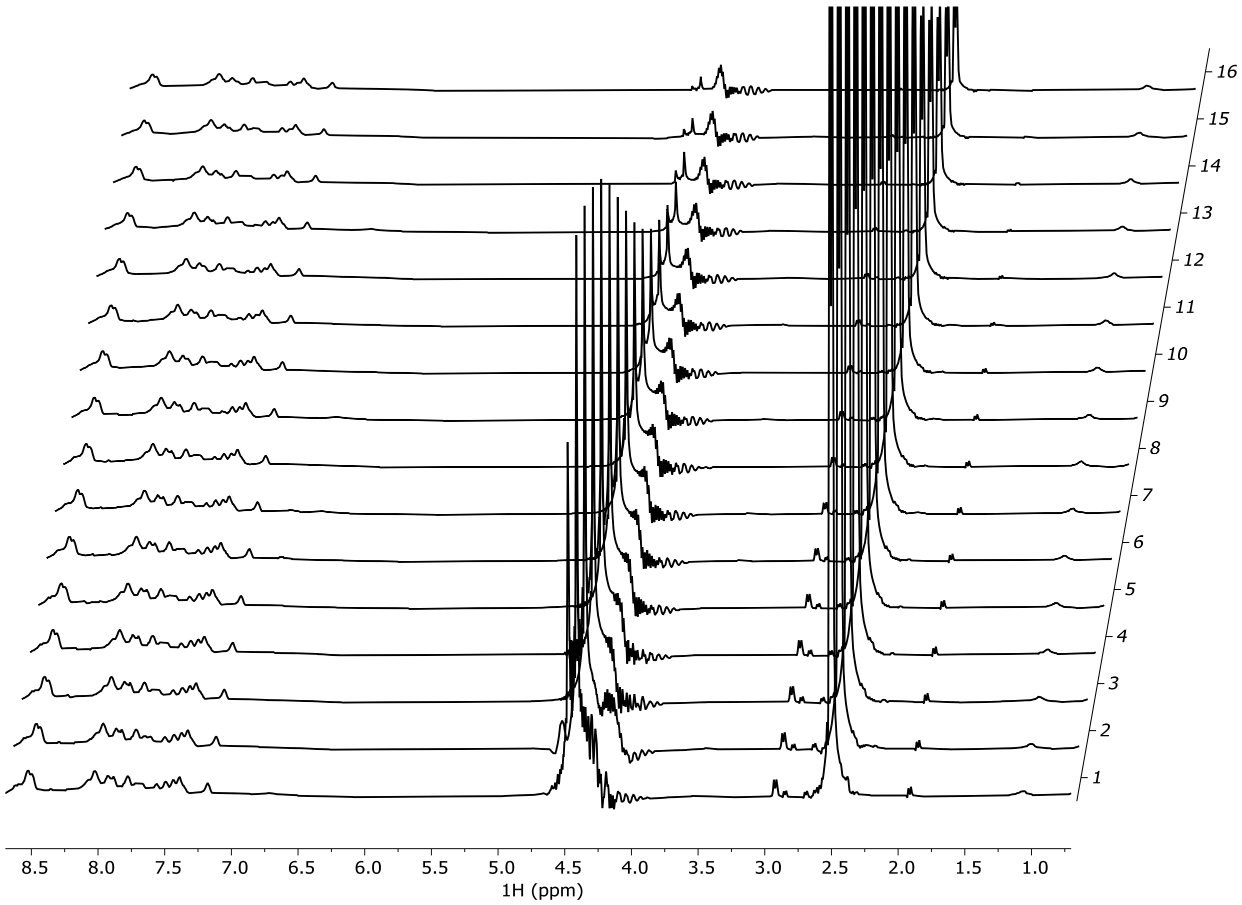

*Figure S21. Arrayed DOSY-NMR spectra of **YRu-3** in D_2O:DMSO-d_6 mixture (75% of D_2O, 25% of DMSO-d_6, 600 MHz).*

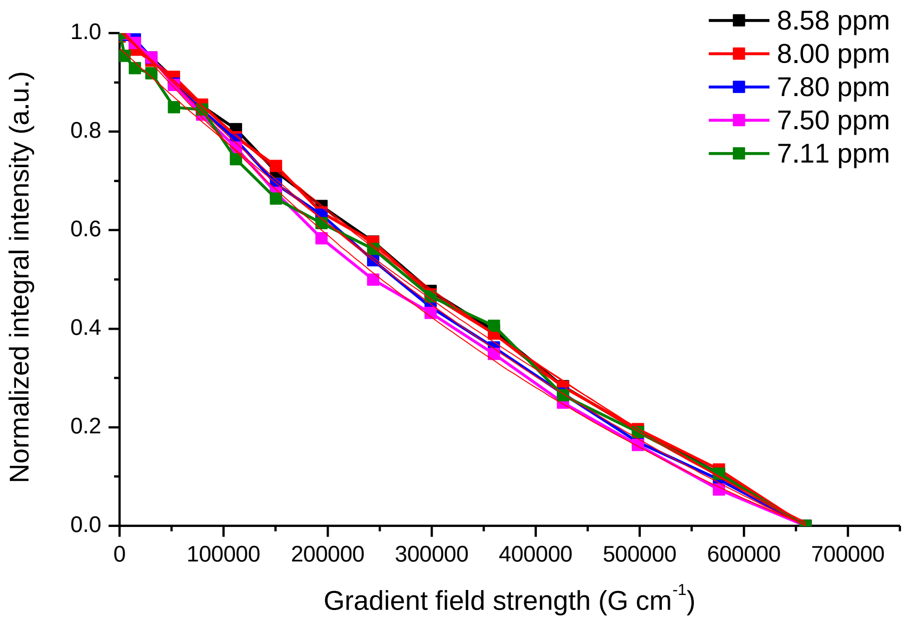

*Figure S22. Normalized integral intensity as a function of the gradient field strength (G·cm^{-1}) for **YRu-3** in D_2O:DMSO-d_6 mixture (75% of D_2O, 25% of DMSO-d_6, 600 MHz). The diffusion coefficient (D) was estimated using monoexponential decay fitting (R^2 > 0.996) and found to be $1.1 \cdot 10^{-10}$ m^2s^{-1}.*

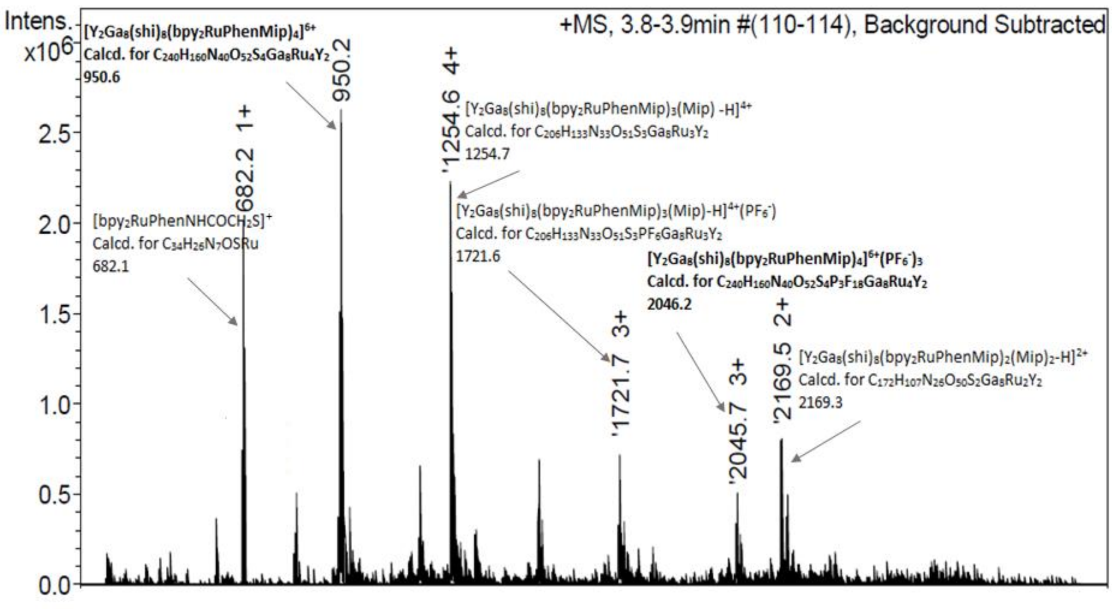

*Figure S23. ESI-MS spectrum of **YRu-3**.*

YbRu-3

Isolated as $[Yb_2Ga_8(shi)_8(bpy_2RuPhenMip)_4][PF_6]_6 \cdot 20.7H_2O \cdot 1.6DMF$ (7231.86 g/mol) (68 mg, 0.009 mmol, 77% yield).

Elemental analysis calcd. for $C_{240}H_{160}N_{40}O_{52}S_4P_6F_{36}Ga_8Ru_4Yb_2 \cdot 20.7H_2O \cdot 1.6DMF$, %: C, 40.66; H, 2.96; N, 8.06; S, 1.77. Found C, 40.50; H, 2.79; N, 8.12; S, 1.60.

ESI-MS, m/z (Figure S24): $[Yb_2Ga_8(shi)_8(bpy_2RuPhenMip)_4]^{6+}$ calcd. for $C_{240}H_{160}N_{40}O_{52}S_4Ga_8Ru_4Yb_2$ 979.1, found 978.7; $\{[Yb_2Ga_8(shi)_8(bpy_2RuPhenMip)_4][PF_6]_2\}^{4+}$ calcd. for $C_{240}H_{160}N_{40}O_{52}S_4P_2F_{12}Ga_8Ru_4Yb_2$ 1540.2, found 1539.5; $\{[Yb_2Ga_8(shi)_8(bpy_2RuPhenMip)_4][PF_6]_3\}^{3+}$ calcd. for $C_{240}H_{160}N_{40}O_{52}S_4P_3F_{18}Ga_8Ru_4Yb_2$ 2100.3, found 2100.9. Species corresponding to fragmentation: $[bpy_2RuPhenNH]^+$ calcd. for $C_{32}H_{24}N_7Ru$ 608.1, found 608.2; $[bpy_2RuPhenNHCOCH_2S]^+$ calcd. for $C_{34}H_{26}N_7OSRu$ 682.1, found 682.2; $[Yb_2Ga_8(shi)_8(bpy_2RuPhenMip)_3(Mip)]^{4+}$ calcd. for $C_{206}H_{134}N_{33}O_{51}S_3Ga_8Ru_3Yb_2$ 1297.7, found 1296.1; $\{[Yb_2Ga_8(shi)_8(bpy_2RuPhenMip)_3(Mip)\text{-}H][PF_6]\}^{3+}$ calcd. for $C_{206}H_{133}N_{33}O_{51}S_3PF_6Ga_8Ru_3Yb_2$ 1777.6, found 1777.6; $[Yb_2Ga_8(shi)_8(bpy_2RuPhenMip)_2(Mip)_2\text{-}2H]^{2+}$ calcd. for $C_{172}H_{106}N_{26}O_{50}S_2Ga_8Ru_2Yb_2$ 2252.3, found 2252.8.

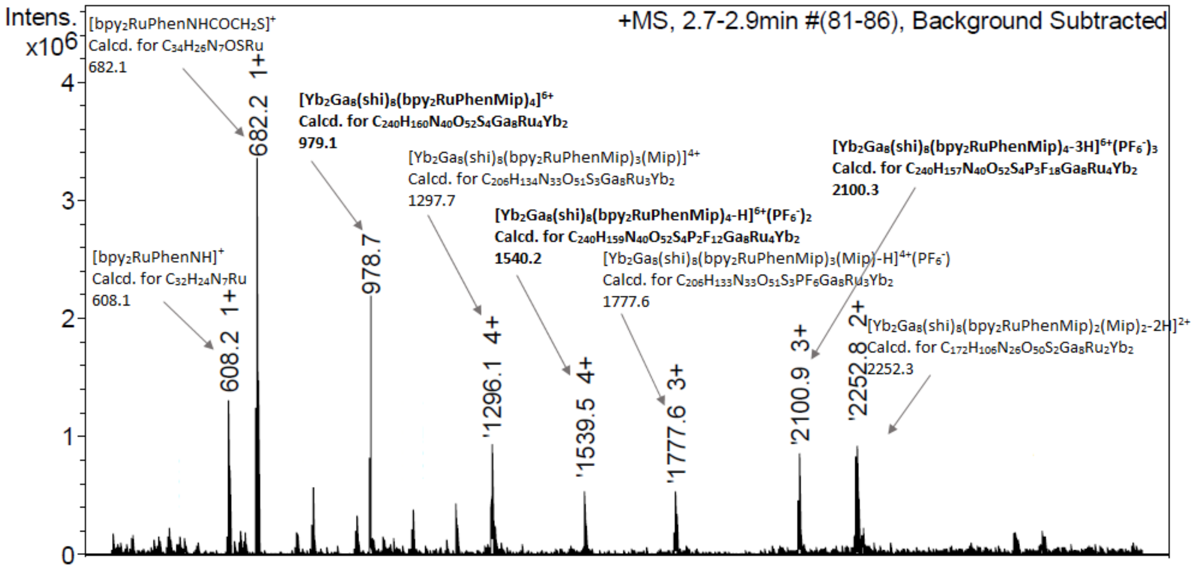

Figure S24. ESI-MS spectrum of ***YbRu-3****.*

ErRu-3

Isolated as [Er_2Ga_8(shi)$_8$(bpy$_2$RuPhenMip)$_4$][PF_6]$_6$·16H_2O (7018.8 g/mol) (15 mg, 0.002 mmol, 9% yield).

Elemental analysis calcd. for $C_{240}H_{192}N_{40}O_{68}S_4P_6F_{36}Ga_8Ru_4Er_2$: C, 41.07; H, 2.76; N, 7.98; S, 1.85. Found C, 41.03; H, 2.55; N, 7.71; S 1.90.

ESI-MS, m/z (Figure S25): [Er_2Ga_8(shi)$_8$(bpy$_2$RuPhenMip)$_4$]$^{6+}$ calcd. for $C_{240}H_{160}N_{40}O_{52}S_4Ga_8Ru_4Er_2$ 976.8, found 977.1. Species corresponding to fragmentation: [bpy$_2$RuPhenNHCOCH$_2$S]$^{1+}$ calcd. for $C_{34}H_{26}N_7OSRu$ 682.1, found 682.1; [Er_2Ga_8(shi)$_8$(bpy$_2$RuPhenMip)$_3$(Mip)-H]$^{4+}$ calcd. for $C_{206}H_{133}N_{33}O_{51}S_3Ga_8Ru_3Er_2$ 1293.7, found 1293.9; [Er_2Ga_8(shi)$_8$(bpy$_2$RuPhenMip)$_2$(Mip)$_2$-2H]$^{2+}$ calcd. for $C_{172}H_{106}N_{26}O_{50}S_2Ga_8Ru_2Er_2$ 2246.8, found 2246.5.

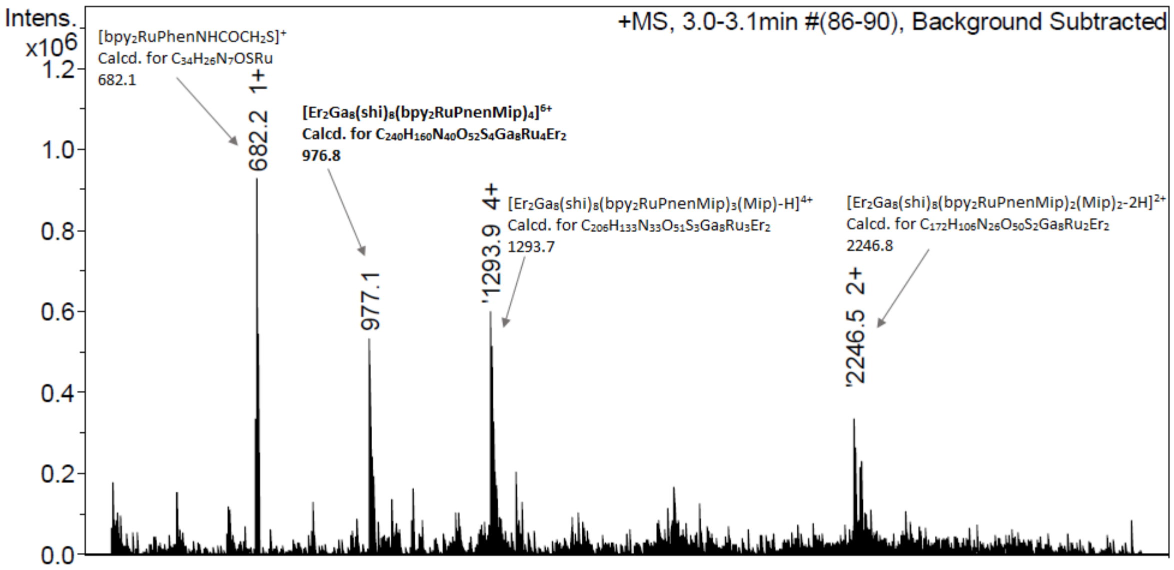

Figure S25. ESI-MS spectrum of ***ErRu-3****.*

NdRu-3

Isolated as $[Nd_2Ga_8(shi)_8(bpy_2RuPhenMip)_4][PF_6]_6{\cdot}3DMF{\cdot}32H_2O$ (7479.7 g/mol) (50mg, 0.007 mmol, 38% yield).

Elemental analysis calcd. for $C_{249}H_{245}N_{43}O_{87}S_4P_6F_{36}Ga_8Ru_4Nd_2$: C, 39.98; H, 3.30; N, 8.05; S, 1.71. Found C, 39.70; H, 2.97; N, 8.43; S, 1.33.

ESI-MS, m/z (Figure S26): $[Nd_2Ga_8(shi)_8(bpy_2RuPhenMip)_4]^{6+}$ calcd. for $C_{240}H_{160}N_{40}O_{52}S_4Ga_8Ru_4Nd_2$ 969.1, found 969.2; $\{[Nd_2Ga_8(shi)_8(bpy_2RuPhenMip)_4][PF_6]_2\}^{4+}$ calcd. For $C_{240}H_{160}N_{40}O_{52}S_4P_2F_{12}Ga_8Ru_4Nd_2$ 1526.2, found 1526.1; $\{[Nd_2Ga_8(shi)_8(bpy_2RuPhenMip)_4\text{-}H][PF_6]_3\}^{3+}$ calcd. For $C_{240}H_{159}N_{40}O_{52}S_4P_3F_{18}Ga_8Ru_4Nd_2$ 2083.2, found 2082.6. Species corresponding to the fragmentation: $[bpy_2RuPhenMipH_2]^{2+}$ calcd. for $C_{46}H_{34}N_8O_7SRu$ 472.0, found 472.0; $[bpy_2RuPhenNHCOCH_2S]^{+}$ calcd. for $C_{34}H_{26}N_7OSRu$ 682.1, found 682.1; $\{[bpy_2RuPhenMipH_2][PF_6]\}^{+}$ calcd. for $C_{46}H_{34}N_8O_7SPF_6Ru$ 1089.1, found 1089.3; $[Nd_2Ga_8(shi)_8(bpy_2RuPhenMip)_3(Mip)]^{4+}$ calcd. for $C_{206}H_{134}N_{33}O_{51}S_3Ga_8Ru_3Nd_2$ 1283.4, found 1282.8; $\{[Nd_2Ga_8(shi)_8(bpy_2RuPhenMip)_3(Mip)\text{-}2H][PF_6]\}^{3+}$ calcd. for $C_{206}H_{131}N_{33}O_{51}S_3PF_6Ga_8Ru_3Nd_2$ 1757.2, found 1757.6; $[Nd_2Ga_8(shi)_8(bpy_2RuPhenMip)_2(Mip)_2\text{-}2H]^{2+}$ calcd. for $C_{172}H_{106}N_{26}O_{50}S_2Ga_8Ru_2Nd_2$ 2223.8, found 2223.3.

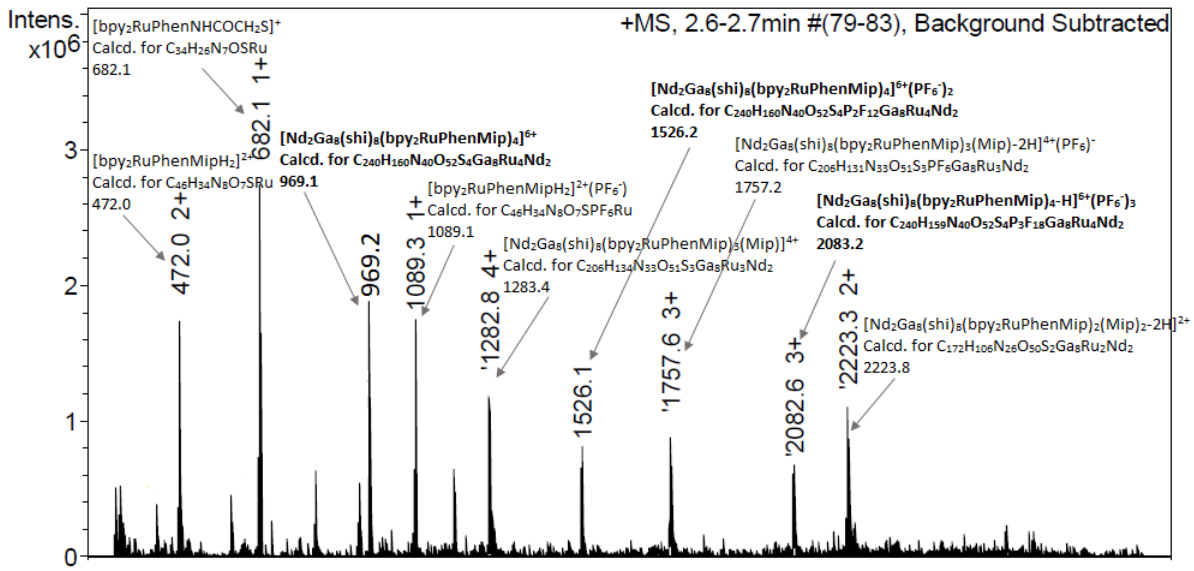

*Figure S26. ESI-MS spectrum of **NdRu-3**.*

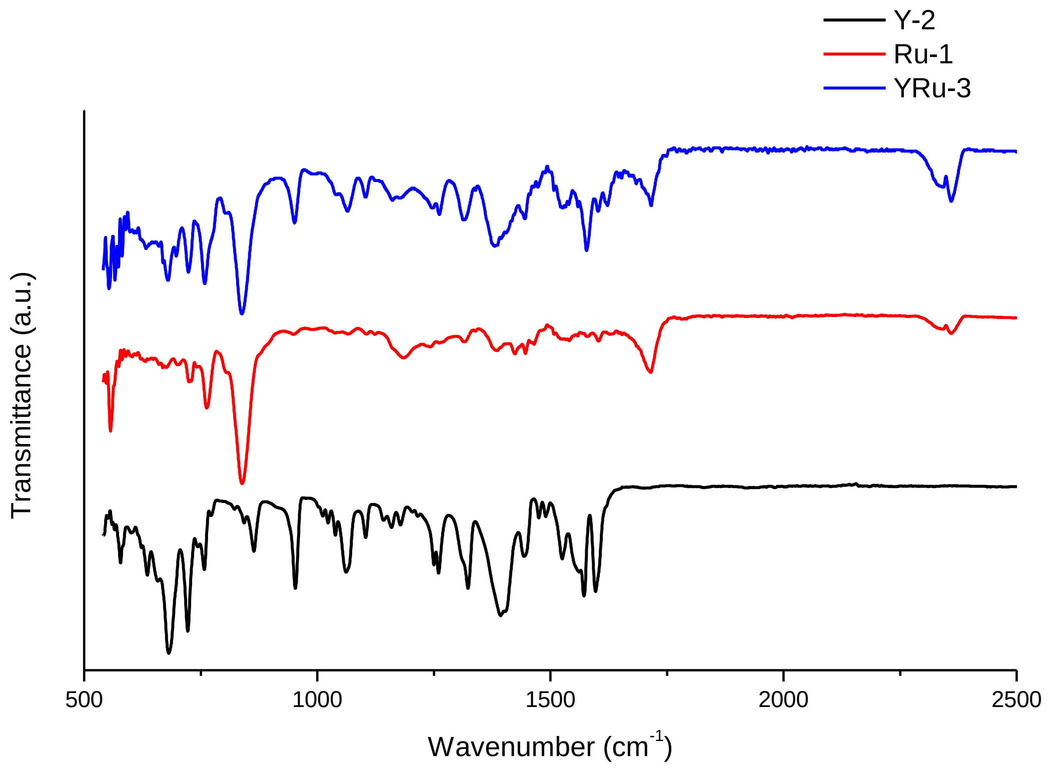

*Figure S27. Comparison of FTIR spectra for **Y-2, Ru-1** and **YRu-3**.*

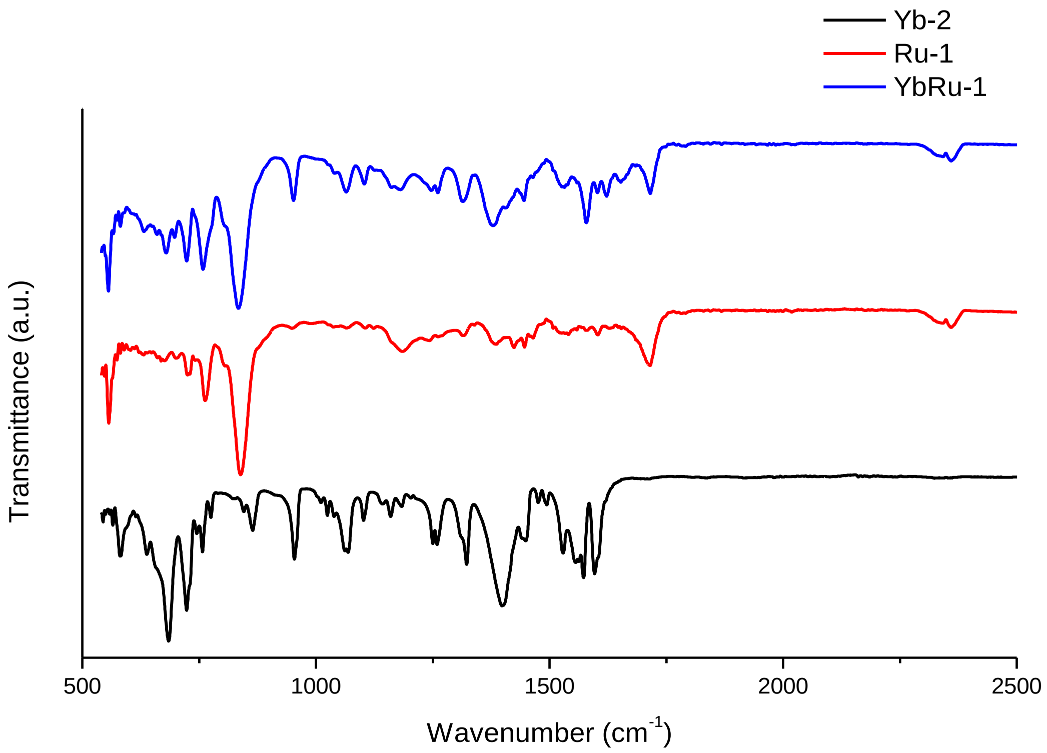

*Figure S28. Comparison of FTIR spectra for **Yb-2, Ru-1** and **YbRu-3**.*

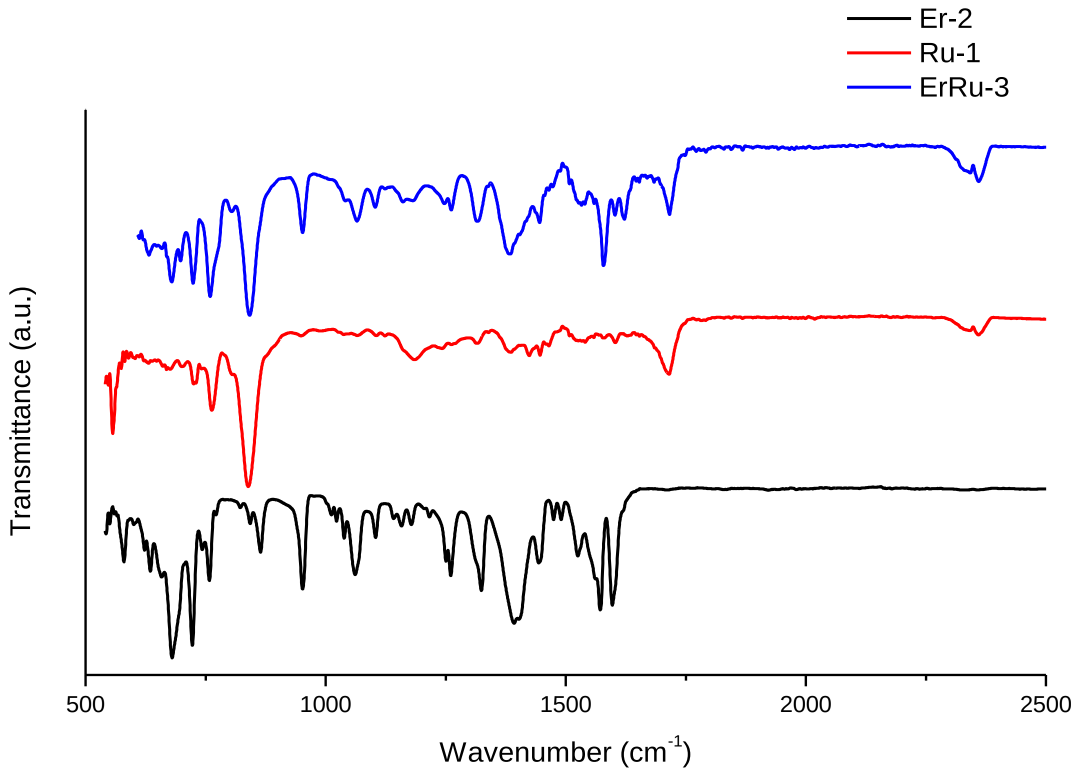

Figure S29. Comparison of FTIR spectra for ***Er-2**, **Ru-1*** *and* ***ErRu-3***.

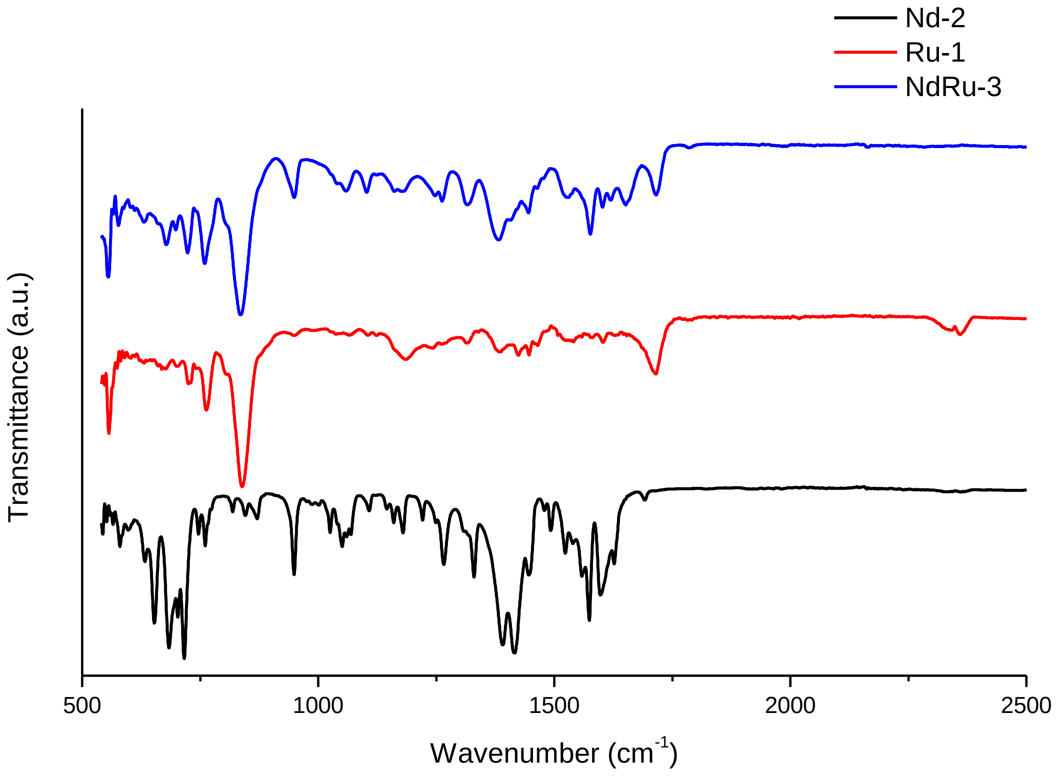

Figure S30. Comparison of FTIR spectra for ***Nd-2**, **Ru-1*** *and* ***NdRu-3***.

Photophysical properties

All photophysical data were acquired on freshly prepared, unless otherwise stated, solutions of Ru^{II} complexes or MCs in air-saturated solutions in DMSO (10 μM), H_2O-DMSO (99:1; 14 μM) and D_2O-DMSO (99:1, 14 μM) as well as in cell culture medium Opti-MEM™-DMSO (99:1, 14 μM) placed in quartz Suprasil® cuvettes or quartz capillaries (2 mm internal diameter) at room temperature.

Absorption spectra

Absorption spectra were measured on a Jasco V-670 UV/Visible/NIR spectrophotometer.

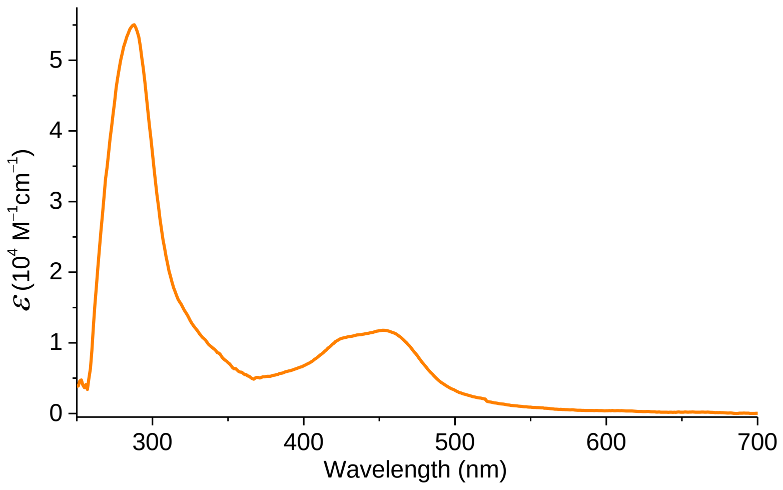

*Figure S31. Absorption spectrum of $bpy_2RuPhenMipH_2$ (**Ru-1**) in DMSO (10 μM).*

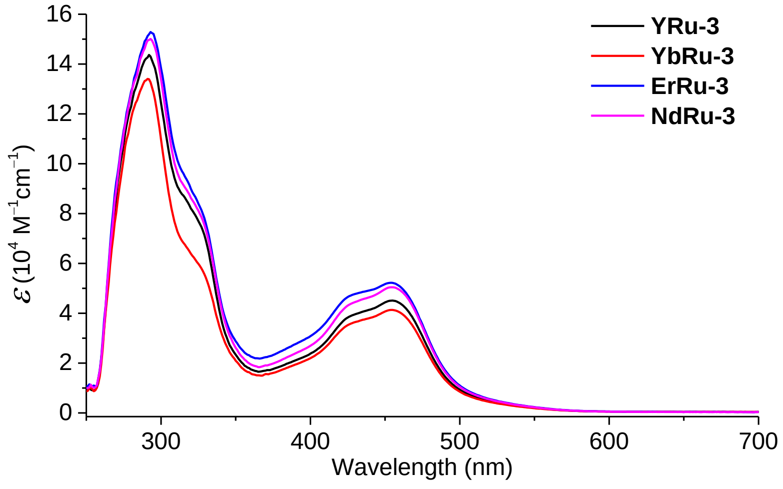

*Figure S32. Absorption spectra of **LnRu-3** (Ln = Y^{III}, Yb^{III}, Er^{III}, Nd^{III}) in DMSO (10 μM).*

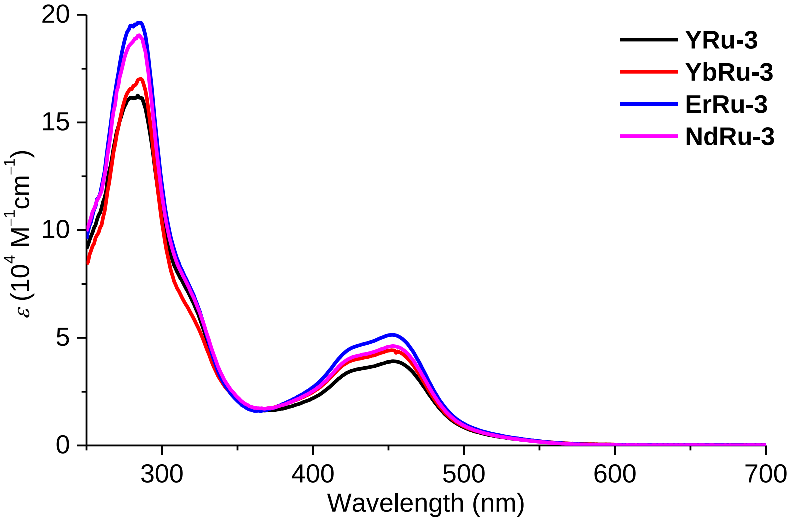

*Figure S33. Absorption spectra of **LnRu-3** (Ln = Y^{III}, Yb^{III}, Er^{III}, Nd^{III}) in H_2O-DMSO (99:1, 14 µM).*

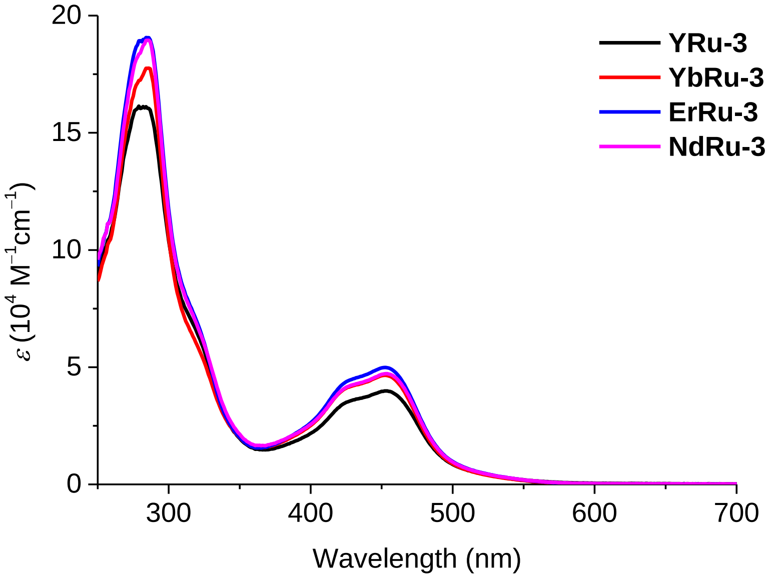

*Figure S34. Absorption spectra of **LnRu-3** (Ln = Y^{III}, Yb^{III}, Er^{III}, Nd^{III}) in H_2O-DMSO (99:1, 14 µM) after 7 days of storage in solution.*

*Table S1. Molar absorption coefficients (ε) for solutions of **Ru-1** and **LnRu-3** (Ln = Y^{III}, Yb^{III}, Er^{III}, Nd^{III}) in DMSO (10 µM) or H_2O-DMSO (99:1, 14 µM).*

**Compound**	***Solvent***	**ε (M^{-1}·cm^{-1})**		
		**290 nm**	**325 nm**	**455 nm**
**Ru-1**	DMSO	55036.3		11727.4
**YRu-3**	DMSO	142687.5	77001.2	45081.4
	H_2O	152950.1	58878.7	39004.4
**YbRu-3**	DMSO	134043.9	59836.3	41299.7
	H_2O	160895.5	53043.8	39004.4
**ErRu-3**	DMSO	151232.2	83992.2	52178.0
	H_2O	185588.4	62185.8	51095.1
**NdRu-3**	DMSO	149543.2	81170.4	50436.1
	H_2O	177616.6	61578.1	45932.2

Emission and excitation spectra, luminescence quantum yields and lifetimes

Steady-state emission and excitation spectra were measured on a custom-designed Horiba Scientific Fluorolog 3-22 spectrofluorimeter equipped with a visible photomultiplier tube (PMT) (220–950 nm, R13456; Hamamatsu) and a NIR PMT (950–1650 nm, H10330-75; Hamamatsu) upon excitation with a continuous Xenon lamp. All excitation and emission spectra were corrected for the instrumental functions. Absolute quantum yields of Ln^{III}-centered transitions in the NIR range under excitation into the MC scaffold (Q_{Ln}^{MC}) at 320 nm or into the Ru^{II}-centered MLCT band at 455 nm (Q_{Ln}^{MLCT}), as well as those of MLCT-centered emission in the visible/NIR range (Q_{MLCT}^{MLCT}), were determined with the help of a Fluorolog 3 spectrofluorimeter based on an absolute method[9] with the use of an integration sphere (Model G8, GMP SA, Renens, Switzerland). Each sample was measured several times under comparable experimental conditions, varying the position of samples. Estimated experimental error for quantum yield determination is ~10 %. Luminescence lifetimes of Ln^{III} emission (τ_{obs}) were determined under excitation at 355 nm provided by a Nd:YAG laser (YG 980; Quantel). The signals of the Ln^{III} were selected using an iHR320 monochromator (Horiba Scientific) and detected with a Hamamatsu H10330-75 PMT. Output signals from the detector were fed into a 500 MHz band-pass digital oscilloscope (TDS 754C; Tektronix), transferred to a PC for data processing with the program Origin® 9. Luminescence lifetimes are averages of at least three independent measurements.

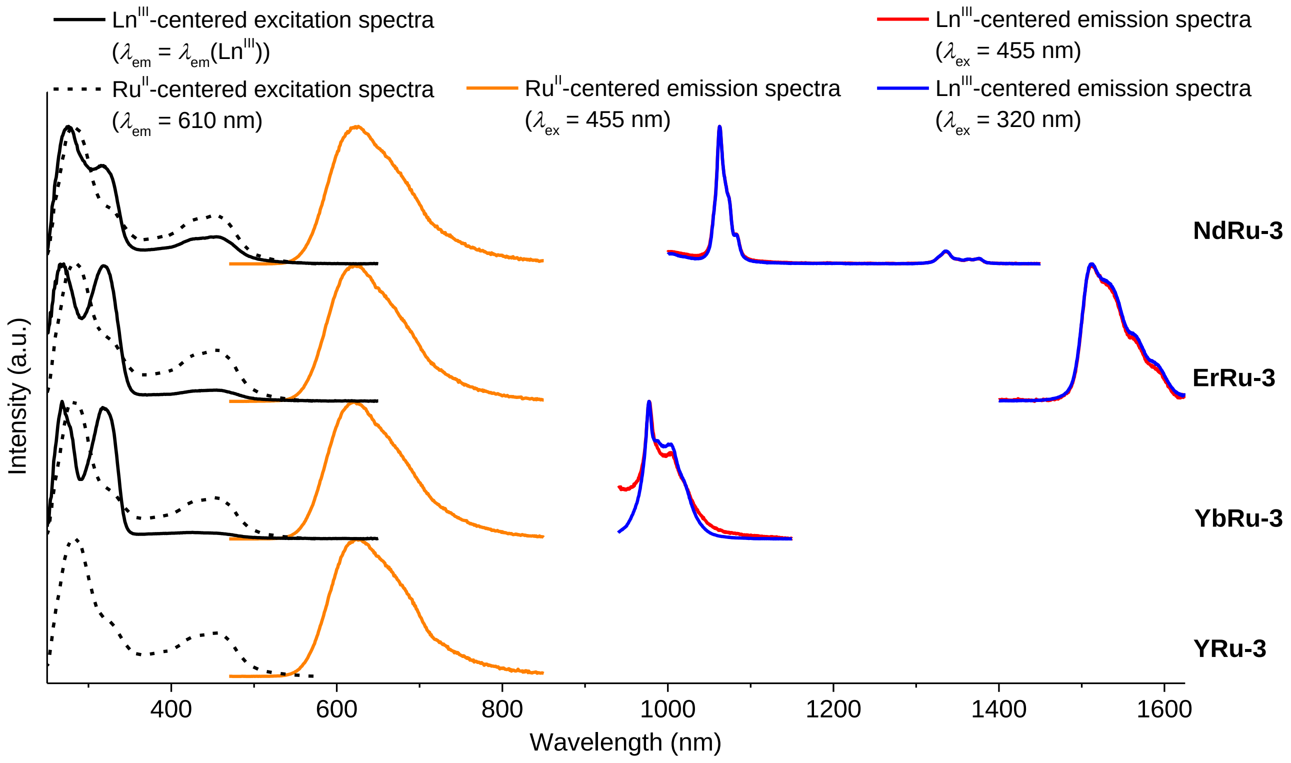

*Figure S35. Corrected and normalized excitation and emission spectra for solutions of **LnRu-3** (Ln = Y^{III}, Yb^{III}, Er^{III}, Nd^{III}) in DMSO (10 μM).*

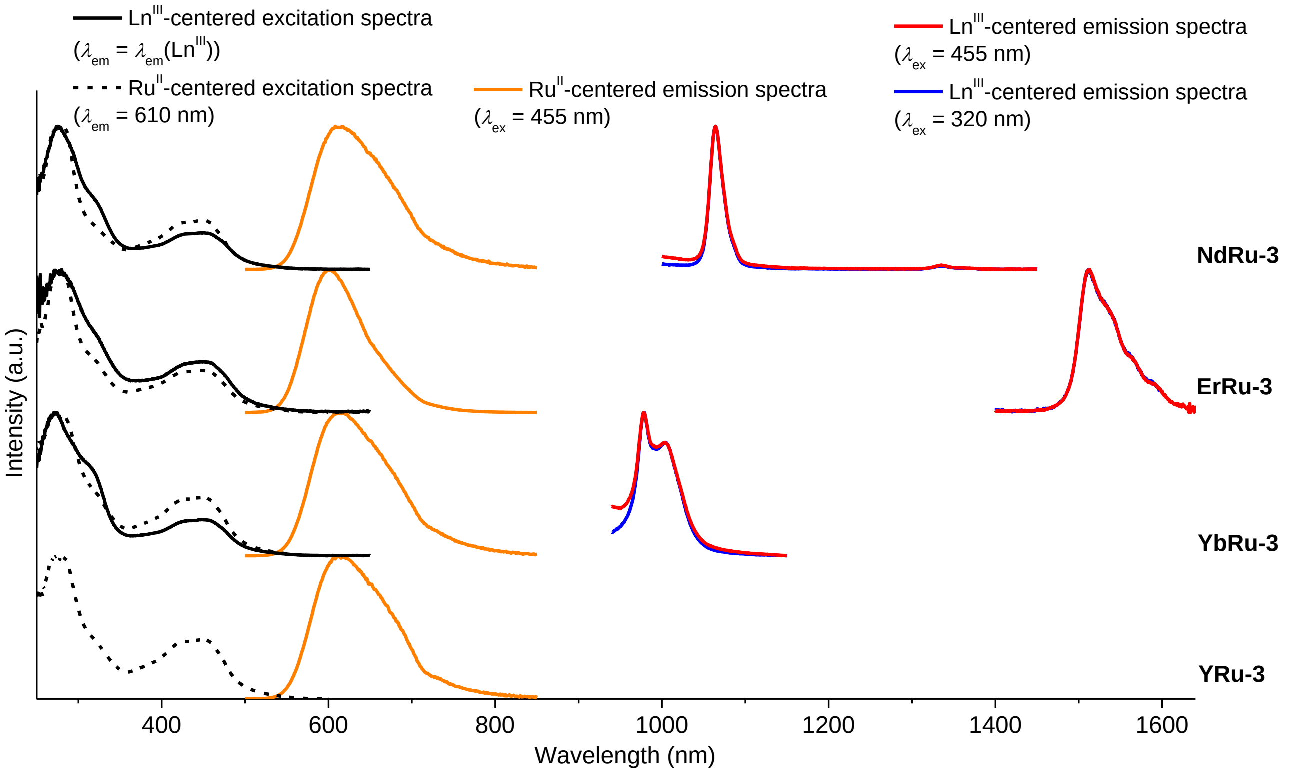

*Figure S36. Corrected and normalized excitation and emission spectra for solutions of **LnRu-3** in H_2O-DMSO (99:1, Ln = Y^{III}, Yb^{III}, Nd^{III}, 14 µM) or D_2O-DMSO (99:1, Ln = Er^{III}, 14 µM).*

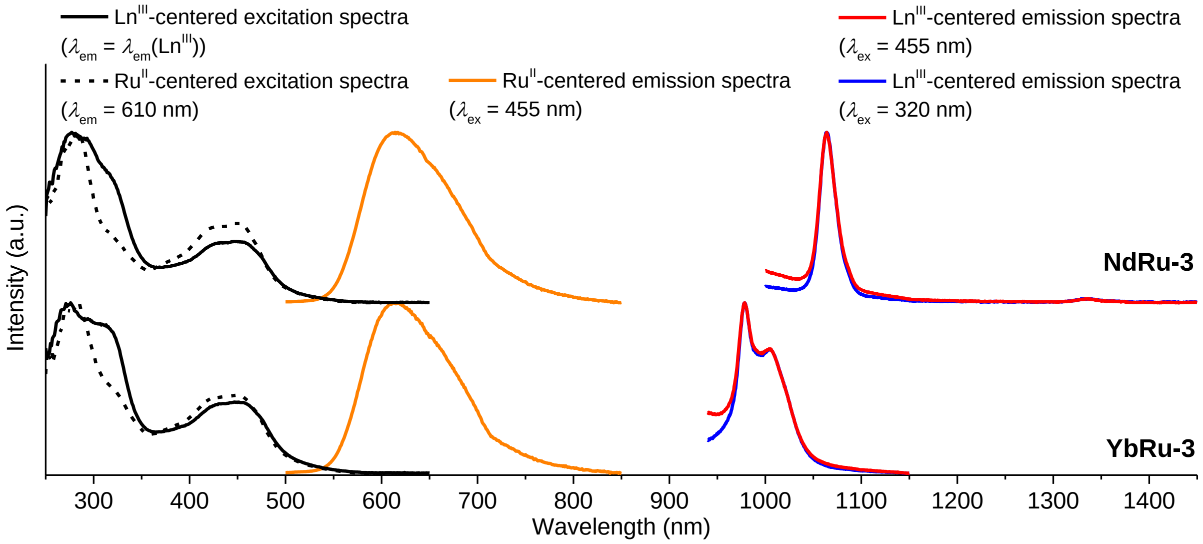

*Figure S37. Corrected and normalized excitation and emission spectra for solutions of **LnRu-3** in Opti-MEM^{TM}-DMSO (99:1, Ln = Yb^{III}, Nd^{III}, 14 µM).*

*Table S2. Photophysical parameters for solutions of **LnRu-3** (Ln = Y^{III}, Yb^{III}, Er^{III}, Nd^{III}) in DMSO (10 µM), H_2O-DMSO (99:1, 14 µM) and D_2O-DMSO (99:1; 14 µM).[a]*

MC	Solvent	Q_{Ln}^{MLCT} (10^{-2} %)[b]	Q_{Ln}^{MC} (%)[c]	τ_{obs}^{Ln} (µs)[d]	Q_{MLCT}^{MLCT} (%)[b]	τ_{obs}^{MLCT} (µs)[e]	η_{ET} (%)[f]	k_{ET} (s^{-1}) [g]
**YRu-3**	DMSO	-	-	-	9.84(1)	0.93(3)	-	-
	H_2O	-	-	-	1.02(9)	0.60(1)	-	-
	D_2O	-	-	-	0.864(5)	0.77(7)	-	-
**YbRu-3**	DMSO	5.23(1)	1.95(3)	54.24(1)	9.74(2)	0.74(1)	28	$2.8 \cdot 10^5$
	H_2O	0.552(3)	$5.52(3) \cdot 10^{-3}$	9.20(3)	0.728(2)	0.59(1)	0.5	$7.7 \cdot 10^3$
	D_2O	4.66(1)	$1.14(9) \cdot 10^{-1}$	20.34(2)	0.952(1)	0.72(6)	6.5	$9.0 \cdot 10^4$
**ErRu-3**	DMSO	0.307(4)	$1.99(4) \cdot 10^{-2}$	9.23(7)	9.96(3)	0.78(3)	21	$2.1 \cdot 10^5$
	H_2O	n.d. [h]	n.d. [h]	n.d. [h]	0.761(7)	0.58(5)	3.5	$6.10 \cdot 10^4$
	D_2O	0.137(2)	$4.30(4) \cdot 10^{-3}$	6.80(5)	0.927(6)	0.68(5)	12	$1.7 \cdot 10^5$
**NdRu-3**	DMSO	8.50(6)	0.149(2)	3.11(1)	8.67(1)	0.67(5)	42	$4.2 \cdot 10^5$
	H_2O	0.579(6)	$5.79(5) \cdot 10^{-3}$	0.74(1)	0.696(8)	0.51(3)	14	$2.8 \cdot 10^5$
	D_2O	1.21(2)	$2.46(1) \cdot 10^{-2}$	2.35(7)	0.919(4)	0.70(3)	9	$1.3 \cdot 10^5$

[a] In air-saturated solvents at room temperature; 2σ values between parentheses; relative errors: τ_{obs}, ±2%; Q, ±10%. [b] λ_{ex} = 455 nm. [c] λ_{ex} = 320 nm. [d] λ_{ex} = 355 nm; λ_{em} = λ_{em} (Ln^{III}). [e] λ_{ex} = 355 nm; λ_{em} = 610 nm. [f] Calculated using the formula: $\eta_{ET} = 100 \times (1 - \frac{\tau_{obs}^{MLCT}\ (LnRu-3)}{\tau_{obs}^{MLCT}\ (YRu-3)})$. [g] Calculated using the formula: $k_{ET} = \frac{1}{\tau_{obs}^{MLCT}\ (LnRu-3)} - \frac{1}{\tau_{obs}^{MLCT}\ (YRu-3)}$. [h] Could not be determined because of insufficient signal intensity.

Note to Table S2: A comparison of the values of τ_{obs}^{MLCT} of NIR-emitting **LnRu-3** with these of **YRu-3** can be used to estimate MLCT-to-Ln energy transfer efficiencies (η_{ET}) and the corresponding rates (k_{ET}). It was shown that the values of η_{ET} and k_{ET} depend on the nature of the solvent and of the NIR-emitting Ln^{III} used. For solutions of **LnRu-3** in DMSO, it was confirmed that MLCT states are best suited for the sensitization of Nd^{III} followed by Yb^{III} and Er^{III}. A similar trend of the intramolecular energy transfer (IET) rates was found using the JOYSpectra web platform (Table S3). On the other hand, considering the large distance between the Ru^{II} chromophore and the Ln^{III} (Table S3, R_L = 11.42 Å for ^{1}MLCT and 9.04 Å for ^{3}MLCT) and the high probability of non-radiative deactivation through vibrations for solutions of **LnRu-3** in H_2O and D_2O, the determination of η_{ET} and k_{ET} using above-mentioned equations is less relable.

Determination of ^{1}MLCT and ^{3}MLCT

The energies of ^{1}MLCT (459.2 nm, 21 780 cm^{-1}) and ^{3}MLCT (611.2 nm, 16 360 cm^{-1}) states were determined as 0-0 transitions from the Gaussian deconvolutions of absorption and emission spectra of **YRu-3**, respectively (Figure S38).

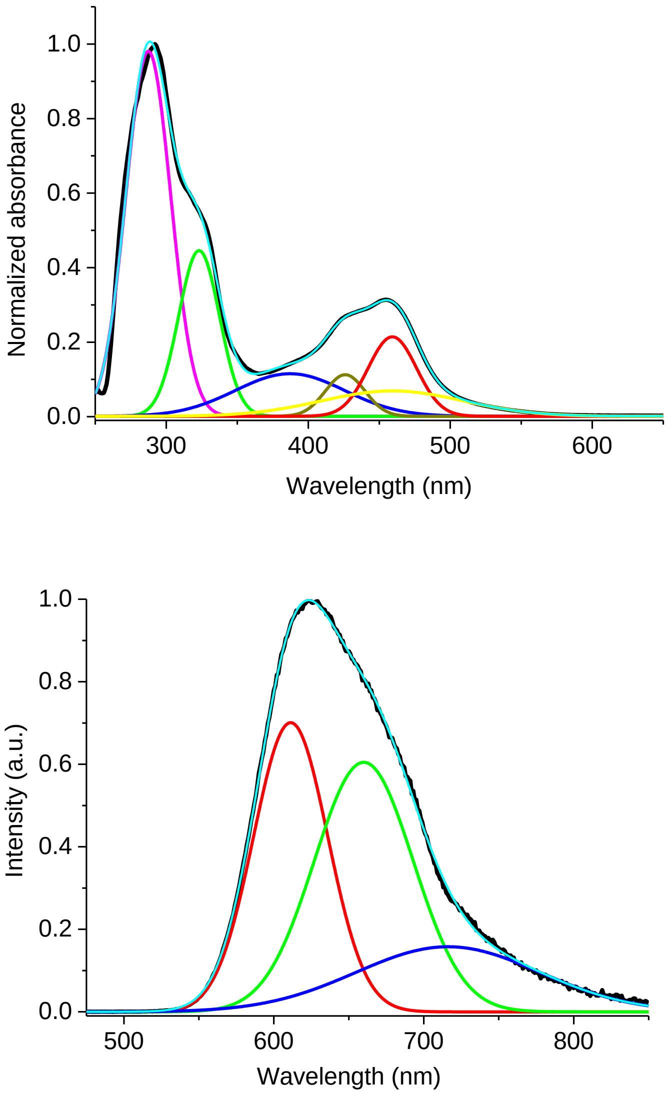

*Figure S38. Normalized (top) absorption and (bottom) emission spectra for solution of **YRu-3** in DMSO (10 µM, black traces) and their Gaussian deconvolution (colored traces). Red traces correspond to 0-0 transitions.*

Imaging in the NIR-II range

Near-infrared (NIR) luminescence imaging experiments were performed using a custom-designed NIR-II Kaer Labs Imaging System (KIS) that includes a ZEPHIR 1.7x camera (Photon etc., Montréal, Québec, Canada). For imaging, solutions of **LnRu-3** (Ln = Yb^{III}, Er^{III}, Nd^{III}) in DMSO (10 µM), H_2O-DMSO (99:1; 14 µM) or D_2O-DMSO (99:1, 14 µM) as well as in cell culture medium Opti-MEM™-DMSO (99:1, 14 µM) were placed into quartz capillaries with 2 mm internal diameters. For each series of imaging experiments, a capillary filled with the corresponding solvent was used as a blank. All measurements were performed at room temperature. The samples were excited using a Nikon C-HGFI Intensilight source combined with a 447 nm bandpass 60 nm filter, while the emission signals of Yb^{III} ($^2F_{5/2}\rightarrow{}^2F_{7/2}$ transition), Nd^{III} ($^4F_{3/2}\rightarrow{}^4I_{11/2}$ or $^4F_{3/2}\rightarrow{}^4I_{13/2}$ transitions) and Er^{III} ($^4I_{13/2}\rightarrow{}^4I_{15/2}$ transition) in the NIR-II range were collected using a 996 nm bandpass 70 nm filter; a 1065 nm bandpass 30 nm filter or a 1365 nm bandpass 130 nm filter, and 1530 nm bandpass 50 nm filter, respectively. Background images (acquired in the absence of the excitation light) were subtracted from the fluorescence images using a Fiji image processing package. The general scheme of the experimental setup is presented on Figure S39A.

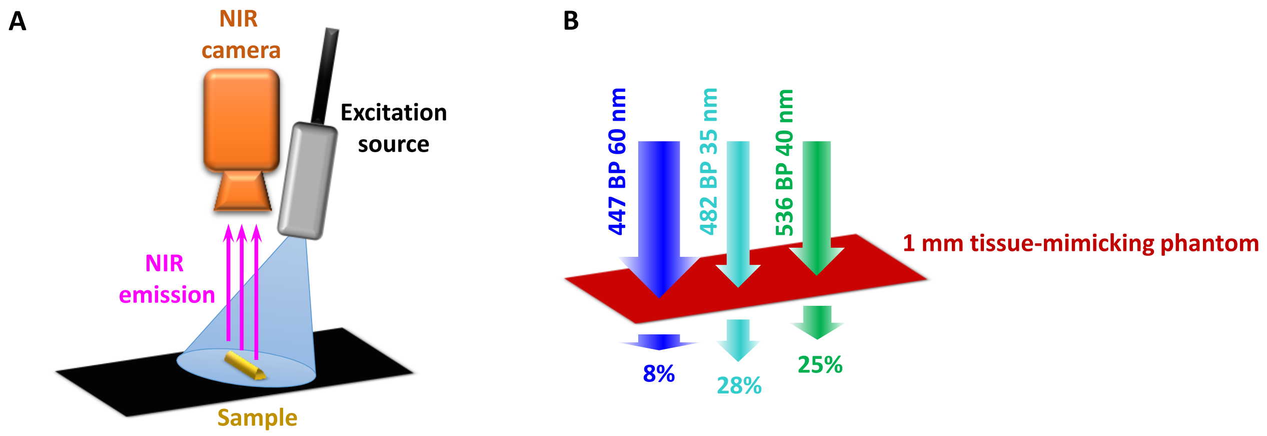

Figure S39. (A) General scheme of the experimental setup used for NIR-II imaging experiments. (B) Attenuation of light selected using different bandpass (BP) filters upon propagation through a 1 mm tissue-mimicking phantom; percentages correspond to the amounts of transmitted light.

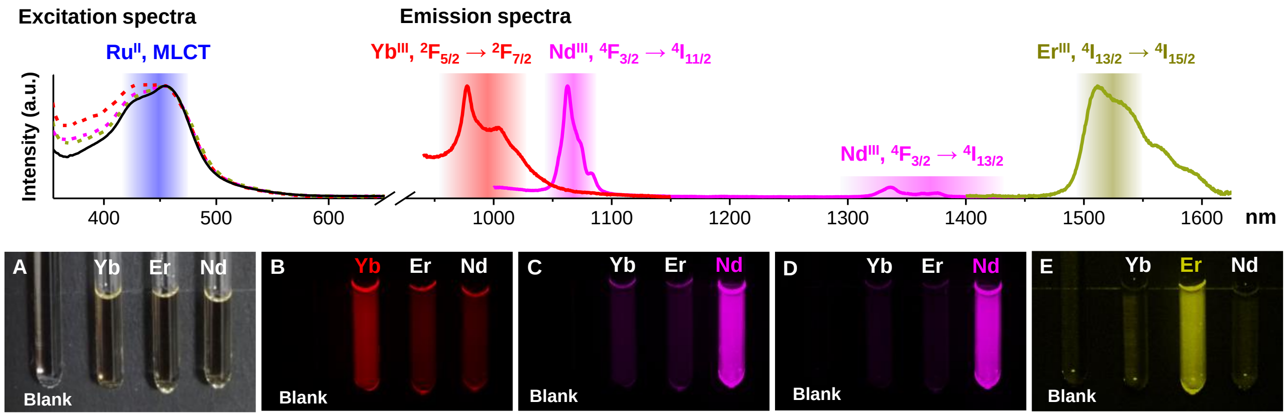

*Figure S40. Photophysical and NIR-II imaging results obtained on solutions of **LnRu-3** (Ln = Yb^{III} (red), Er^{III} (green-yellow), Nd^{III} (magenta)) in DMSO (14 µM). (Top) Excitation (dashed colored traces; λ_{em} = 980 nm (Yb^{III}), 1064 nm (Nd^{III}) or 1525 nm (Er^{III})) and emission (solid colored traces; λ_{ex} = 455 nm) spectra. The absorption spectrum of **YbRu-3** (solid black trace) is overlapped for comparison with the excitation spectra. Shaded rectangular represent the range of wavelengths covered by different bandpass filters used in the NIR-II imaging experiments (vide infra). (Bottom) Color images of quartz capillaries (2 mm internal diameter) filled with solutions of **LnRu-3** (A) and NIR-II luminescence images obtained upon excitation with a light selected using a 447 nm bandpass 60 nm filter and monitoring emission signals of Yb^{III} (B: 996 nm bandpass 70 nm filter, τ_{exp} = 0.5 s), Nd^{III} (C, G: 1065 nm bandpass 30 nm filter, τ_{exp} = 0.5 s; D: 1365 nm bandpass 130 nm filter, τ_{exp} = 2 s) or Er^{III} (E: 1530 nm bandpass 50 nm filter, τ_{exp} = 10 s).*

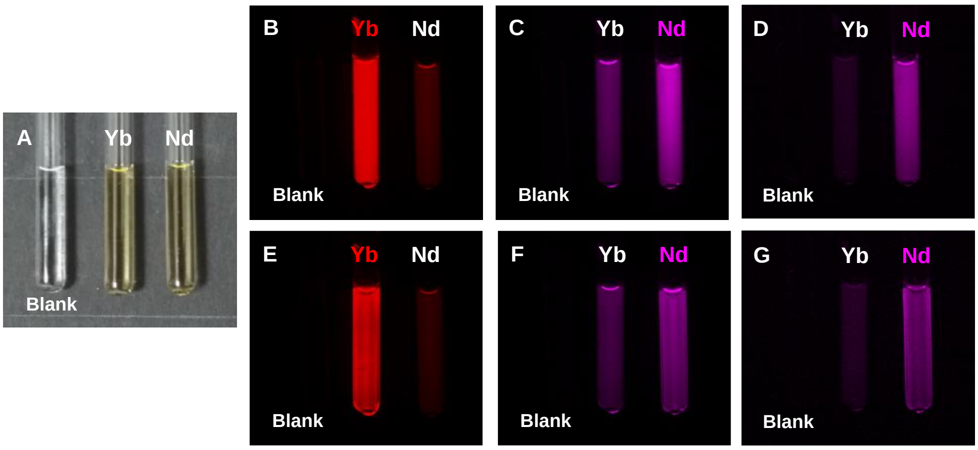

*Figure S41. Color images of quartz capillaries (2 mm internal diameter) filled with solutions of **LnRu-3** (Ln = Yb^{III}, Nd^{III}) in Opti-MEM^{TM}-DMSO (99:1, 14 µM) (A) and NIR-II luminescence images obtained upon excitation with a light selected using a 447 nm bandpass 60 nm filter and monitoring emission signals of Yb^{III} (B, E: 996 nm bandpass 70 nm filter, τ_{exp} = 1 s), Nd^{III} (C, F: 1065 nm bandpass 30 nm filter, τ_{exp} = 2 s; D, G: 1365 nm bandpass 130 nm filter, τ_{exp} = 5 s). E, F and G images were acquired for the same solutions after 48 h of storage. A capillary filled with Opti-MEM^{TM}-DMSO (99:1) medium was used as a blank.*

For solutions of **YbRu-3** and **NdRu-3** in Opti-MEM^{TM} cell culture medium, images in the NIR-II range were also acquired through a 1 mm tissue-mimicking phantom (Figure S42). The phantom was prepared according to a protocol adapted from the references [10,11]. 30 mg of NaN_3 were added to 1.5 mL of 20x concentrated Tris-buffered saline (TBS, 50 mM Tris and 150 mM NaCl). The volume was increased up to 30 mL using Milli-Q water and the pH was adjusted to 7.4 using 0.1 M HCl/NaOH. The solution was heated up to 50°C and gelatine (3 g) was added under constant stirring. After the complete dissolution of the gelatine, the mixture was cooled down to 35°C, bovine hemoglobin (330 mg) and intralipid 20% emulsion (1.5 mL) were added under stirring. The obtained viscous solution was gently poured into a 1 mm electrophoresis glass plate, kept at 4°C for 1 h and used afterwards. The samples were excited using a Nikon C-HGFI Intensilight source combined with a 447 nm bandpass 60 nm filter, a 482 nm bandpass 35 nm filter or a 536 nm bandpass 40 nm filter,

while the emission of Yb^{III} ($^2F_{5/2}\rightarrow{}^2F_{7/2}$ transition) or Nd^{III} ($^4F_{3/2}\rightarrow{}^4I_{11/2}$ or $^4F_{3/2}\rightarrow{}^4I_{13/2}$ transitions) was collected using a 996 nm bandpass 70 nm filter, a 1065 nm bandpass 30 nm filter or a 1365 nm bandpass 130 nm, respectively. The attenuation of the excitation light by a 1 mm tissue-mimicking phantom was estimated by comparing intensities of light selected using a 447 nm bandpass 60 nm filter, a 482 nm bandpass 35 nm filter or a 536 nm bandpass 40 nm filter without and with a tissue-mimicking phantom positioned on the power sensor slide (Figure S39B). Intensities of light were measured using power meter PM200 (Thorlabs) combined with slide power sensor (Thorlabs, model S170C).

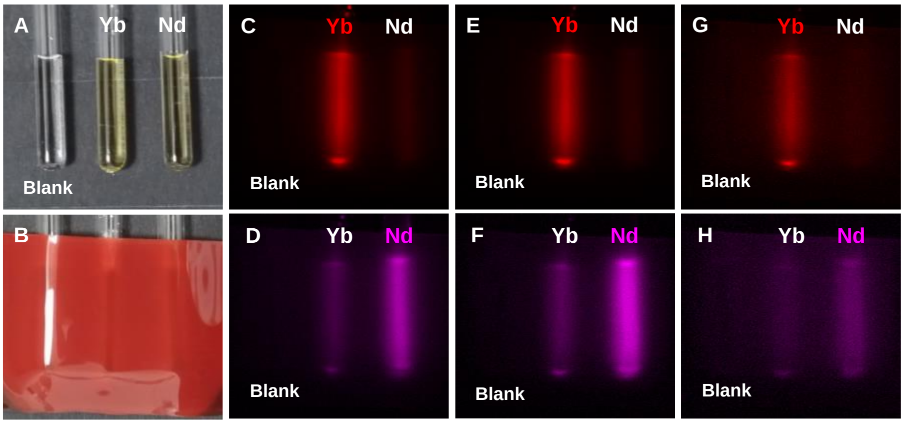

Figure S42. Color images of quartz capillaries (2 mm internal diameter) filled with solutions of ***LnRu-3*** *(Ln = Yb^{III}, Nd^{III}) in Opti-MEM^{TM}-DMSO (99:1, 14 µM) (A) and covered with a 1 mm tissue-mimicking phantom (B). NIR-II luminescence images upon excitation with light selected using a 447 nm bandpass 60 nm filter (C, D), a 482 nm bandpass 35 nm filter (E, F) or a 536 nm bandpass 40 nm filter (G, H). Yb^{III} signal was collected with a 996 nm bandpass 70 nm filter (C, E, G; τ_{exp} = 5 s) while that of Nd^{III} with a 1065 nm bandpass 30 nm filter (D, F, H; τ_{exp} = 10 s). A capillary filled with Opti-MEM^{TM}-DMSO (99:1) medium was used as a blank.*

Computational details

In order to identify the preferred conformation of the **Ru-1** ligand, a noncovalent interaction (NCI)/iMTD algorithm in the conformer-rotamer ensemble sampling tool (CREST 2.12)[12,13] coupled with the extended tight binding GFN2-xTB(ALPB, DMSO) method[14,15] was applied. **Ru-1** ligand was calculated to have a bent geometry (∠C–S–C ≈ 102°) stabilized by attractive C–H···O, C–H···S, and N–H···S interactions. The most energetically stable conformer was selected according to the calculated Gibbs free energies. Four **Ru-1** ligands were then connected with two [$LnGa_4(shi)_4$] MC moieties for further calculations of the most preferable conformation of **LnRu-3**[16] and the corresponding excited state energies[17]. This connection leads to a twisting of one MC moiety relative to the other one by *ca*. 45° that allows ligands to establish short C–H···O contacts (r = 2.32–2.40 Å) with both MC moieties and to interact effectively with MC aromatic rings via π-π stacking. Considering that the MC structure within a series of monomeric[1] or dimeric complexes[18–20] is usually independent of the nature of the Ln^{III}, calculations were performed for the diamagnetic **YRu-3** analogue (the difference between the ionic radius of Y^{III} and those of Yb^{III}, Er^{III} or Nd^{III} lie within 0.02–0.10 Å).[21]

Structural parameters of the **YRu-3** complex in its ground (S_0) and excited (T_1) electronic states were calculated using the density functional theory (DFT) method with the D3 version of Grimme's dispersion correction with Becke-Johnson damping function (Figure S43).[22,23] The PBE[24] exchange-correlation functional combined with the SARC-ZORA-TZVP (on Y^{III} and Ru^{II} atoms)[25] and the ZORA-def2-SVP (on the rest of atoms) all-electron basis sets[26] were employed (later on denoted as ZORA-def2-SVP*). Furthermore, the ZORA scalar relativistic Hamiltonian[27] and the SMD continuum solvation model[28] with dimethyl sulfoxide (DMSO, ε = 46.83) as a solvent were used in all DFT computations.

To estimate the energy positions of the ^{1}MLCT and ^{3}MLCT electronic levels, the energies of the singlet-singlet and singlet-triplet vertical electronic transitions were calculated for the **YRu-3** complex within the spin-orbit coupling time-dependent DFT (SOC-TD-DFT) formalism[29] employing the hybrid PBE0-D3(BJ) functional[30] combined with the ZORA-def2-SVP* basis set on top of the relaxed T_1 structure. The donor–acceptor distances (R_L), which are essential for the energy transfer calculations, were obtained by means of the hole-electron analysis[31] based on the results of TD-DFT calculations.

The RIJCOSX (RI-J) resolution of the identity approximation[32,33] with auxiliary SARC/J basis sets[34] were used in PBE0-D3(BJ) (PBE-D3(BJ)) computations to accelerate the convergence.

All DFT calculations were carried out with the ORCA 5.0.4[35] software package, while GFN2-xTB calculations and the hole-electron analysis were performed using xTB 6.6.1[36] and MultiWFN 3.8[37] programs, respectively. The intramolecular energy transfer (IET) rates (Table S3-Table S15) were computed for **LnRu-3** (Ln = Yb^{III}, Er^{III}, Nd^{III}) complexes using the JOYSpectra web platform[38] with the temperature set to

T = 298.15 K and R_L values corresponding to the shortest donor–acceptor distances (for theoretical background related to the calculations of IET rates see Ref.[39]).

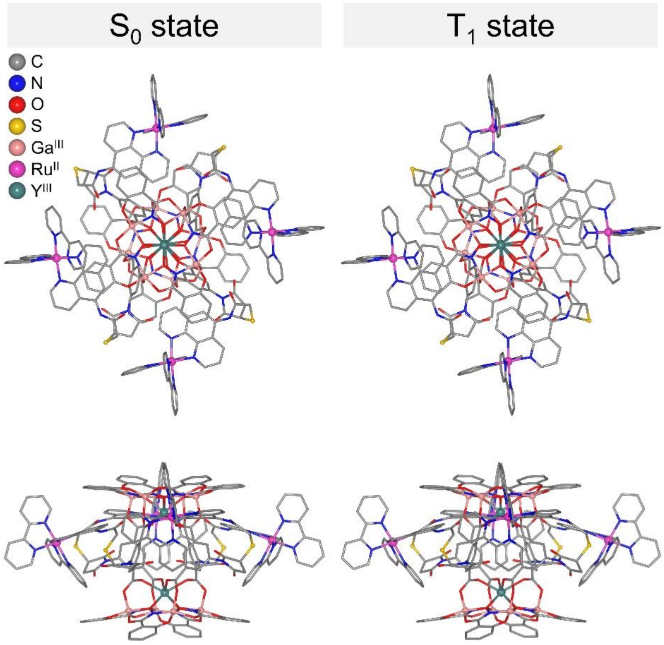

*Figure S43. Structures of the **YRu-3** complex in its ground (S_0) and excited (T_1) electronic states optimized at SMD-ZORA-PBE-D3(BJ)/ZORA-def2-SVP* level of theory. Top and side views are shown. Hydrogens are omitted for clarity.*

*Table S3. Experimental and theoretical singlet (^{1}MLCT, S_1^{MC}, cm^{-1}) and triplet (^{3}MLCT, T_1^{MC}, cm^{-1}) excited states energies for **LnRu-3** MCs as well as forward (W^S and W^T) and backward (W_b^S and W_b^T) intramolecular energy transfer rates.*

State	Parameter	NdRu-3	ErRu-3	YbRu-3
^{1}MLCT	E (cm^{-1})	21780[a] (20300)[b]	21780[a] (20300)[b]	21780[a] (20300)[b]
	R_L (Å)	11.42	11.42	11.42
	W^S (s^{-1})	$4.07{\cdot}10^3$	$1.24{\cdot}10^2$	$3.04{\cdot}10^{-1}$
	W_b^S (s^{-1})	$4.30{\cdot}10^2$	$6.93{\cdot}10^1$	$2.78{\cdot}10^{-25}$
^{3}MLCT	E (cm^{-1})	16360[a] (17331)[b]	16360[a] (17331)[b]	16360[a] (17331)[b]
	R_L (Å)	9.04	9.04	9.04
	W^S (s^{-1})	$1.84{\cdot}10^{-1}$	$2.68{\cdot}10^{-2}$	$2.09{\cdot}10^3$
	W_b^S (s^{-1})	$6.49{\cdot}10^0$	$4.51{\cdot}10^{-2}$	$4.36{\cdot}10^{-10}$

[a] Experimental, this work. [b] Theoretical (TD-DFT, see Computational details), this work. [c] From Ref.[18]

Note to Table S3: It should be noted that the absolute values of theoretical IET rates are several orders of magnitude lower than the experimental ones. This discrepancy can be explained by the long donor-acceptor distance and the contribution of other energy transfer mechanisms (such as phonon-assisted and Dexter) to the sensitization of Ln^{III} NIR emission in **LnRu-3** which are not considered in the JOYSpectra platform.[40,41]

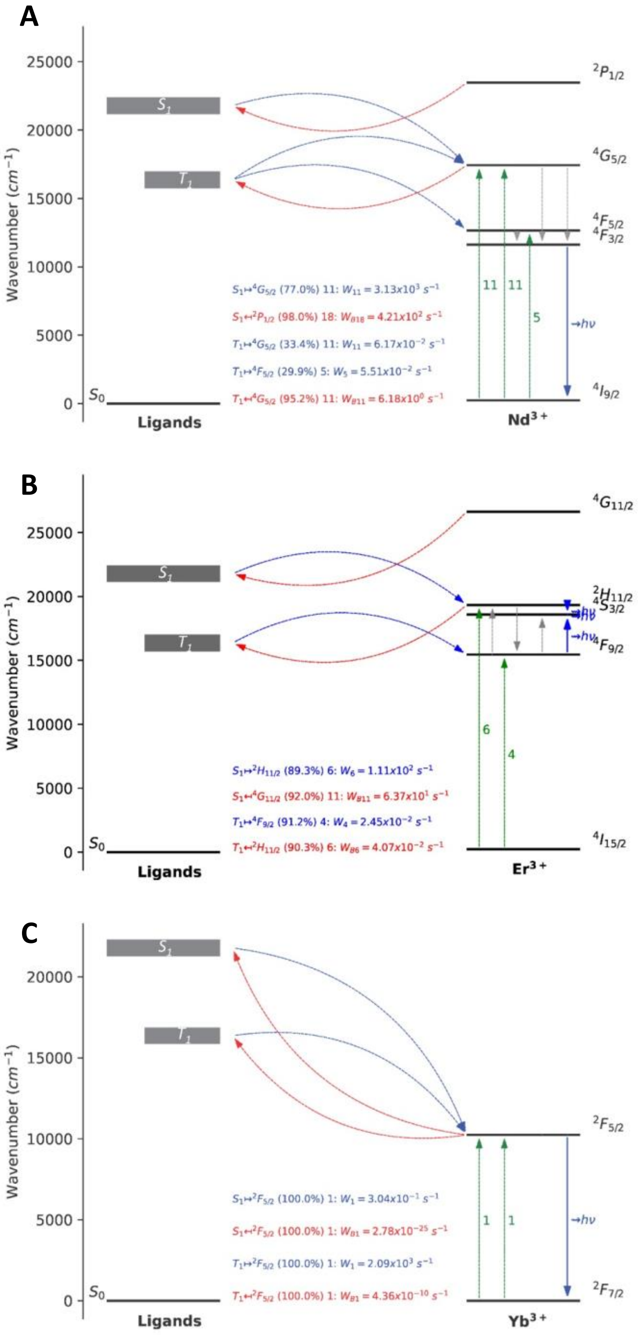

Figure S44. Schematic energy level diagrams for the (A) ***NdRu-3****, (B)* ***ErRu-3****, and (C)* ***YbRu-3*** *complexes. The forward and backward intramolecular energy transfer (IET) pathways with the highest relative contributions are shown by blue and red arrows, respectively (numbers correspond to specific IET pathways, which are listed in Tables S4–S15). S_1 and T_1 labels stand for ^{1}MLCT and ^{3}MLCT states.*

*Table S4. Forward (^{1}MLCT to Nd^{III}) intramolecular energy transfer rates (W, s^{-1}) calculated for the **NdRu-3** complex. Δ (cm^{-1}) is the donor-acceptor energy difference, W(%) is the percentage of the pathway, W_{total} is the sum of the dipole-dipole ($W_{d–d}$), dipole-multipole ($W_{d–m}$), and exchange (W_{ex}) rates of the mechanisms.*

Pathway	Transition			*W*(%)	Δ	W_{total}	$W_{d–d}$	$W_{d–m}$	W_{ex}
1	$^4I_{9/2}$	→	$^4I_{11/2}$	0.00	19901	2.71E-11	3.71E-16	2.71E-11	0.00E+00
2	$^4I_{9/2}$	→	$^4I_{13/2}$	0.00	17917	3.48E-10	4.42E-14	3.47E-10	0.00E+00
3	$^4I_{9/2}$	→	$^4I_{15/2}$	0.00	15867	9.78E-11	8.92E-13	9.70E-11	0.00E+00
4	$^4I_{9/2}$	→	$^4F_{3/2}$	0.00	10394	7.71E-02	2.72E-07	7.71E-02	0.00E+00
5	$^4I_{9/2}$	→	$^4F_{5/2}$	0.01	9355	3.96E-01	3.45E-06	3.96E-01	0.00E+00
6	$^4I_{9/2}$	→	$^2H_{9/2}$	0.01	9247	2.39E-01	9.70E-07	2.39E-01	0.00E+00
7	$^4I_{9/2}$	→	$^4F_{7/2}$	0.01	8396	3.30E-01	1.04E-05	3.30E-01	0.00E+00
8	$^4I_{9/2}$	→	$^4S_{3/2}$	0.00	20646	1.99E-14	6.80E-18	1.99E-14	0.00E+00
9	$^4I_{9/2}$	→	$^4F_{9/2}$	0.01	7116	5.69E-01	5.05E-06	5.69E-01	0.00E+00
10	$^4I_{9/2}$	→	$^2H_{11/2}$	0.01	5910	2.55E-01	4.34E-06	2.55E-01	0.00E+00
11	$^4I_{9/2}$	→	$^4G_{5/2}$	76.99	4587	3.13E+03	1.29E-03	3.13E+03	0.00E+00
12	$^4I_{9/2}$	→	$^4G_{7/2}$	17.37	2722	7.07E+02	6.99E-04	7.07E+02	0.00E+00
13	$^4I_{9/2}$	→	$^4G_{9/2}$	2.54	2306	1.04E+02	2.89E-04	1.04E+02	0.00E+00
14	$^4I_{9/2}$	→	$^2K_{13/2}$	2.03	2230	8.26E+01	1.44E-04	8.26E+01	0.00E+00
15	$^4I_{9/2}$	→	$^2D_{3/2}$	0.67	590	2.73E+01	7.18E-05	2.73E+01	0.00E+00
16	$^4I_{9/2}$	→	$^4G_{11/2}$	0.18	301	7.17E+00	5.95E-05	7.17E+00	0.00E+00
17	$^4I_{9/2}$	→	$^2K_{15/2}$	0.18	235	7.19E+00	9.59E-05	7.19E+00	0.00E+00
18	$^4I_{9/2}$	→	$^2P_{1/2}$	0.00	-1443	4.42E-02	1.16E-07	4.42E-02	0.00E+00
19	$^4I_{9/2}$	→	$^2D_{5/2}$	0.00	-1989	1.39E-05	4.78E-10	1.39E-05	0.00E+00
20	$^4I_{9/2}$	→	$^2P_{3/2}$	0.00	-4409	1.79E-10	8.01E-16	1.79E-10	0.00E+00

*Table S5. Backward (Nd^{III} to ^{1}MLCT) intramolecular energy transfer rates (W, s^{-1}) calculated for the **NdRu-3** complex. Δ (cm^{-1}) is the donor-acceptor energy difference, W(%) is the percentage of the pathway, W_{total} is the sum of the dipole-dipole ($W_{d–d}$), dipole-multipole ($W_{d–m}$), and exchange (W_{ex}) rates of the mechanisms.*

Pathway	Transition			*W*(%)	Δ	W_{total}	$W_{d–d}$	$W_{d–m}$	W_{ex}
1	$^4I_{11/2}$	→	$^4I_{9/2}$	0.00	-19901	4.35E-53	5.94E-58	4.35E-53	0.00E+00
2	$^4I_{13/2}$	→	$^4I_{9/2}$	0.00	-17917	6.78E-48	8.63E-52	6.78E-48	0.00E+00
3	$^4I_{15/2}$	→	$^4I_{9/2}$	0.00	-15867	3.28E-44	2.98E-46	3.25E-44	0.00E+00
4	$^4F_{3/2}$	→	$^4I_{9/2}$	0.00	-10394	3.81E-23	1.34E-28	3.81E-23	0.00E+00
5	$^4F_{5/2}$	→	$^4I_{9/2}$	0.00	-9355	1.76E-20	1.54E-25	1.76E-20	0.00E+00
6	$^2H_{9/2}$	→	$^4I_{9/2}$	0.00	-9247	9.97E-21	4.05E-26	9.97E-21	0.00E+00
7	$^4F_{7/2}$	→	$^4I_{9/2}$	0.00	-8396	1.08E-18	3.39E-23	1.08E-18	0.00E+00
8	$^4S_{3/2}$	→	$^4I_{9/2}$	0.00	-20646	3.21E-57	1.10E-60	3.21E-57	0.00E+00
9	$^4F_{9/2}$	→	$^4I_{9/2}$	0.00	-7116	6.94E-16	6.16E-21	6.94E-16	0.00E+00
10	$^2H_{11/2}$	→	$^4I_{9/2}$	0.00	-5910	8.56E-14	1.46E-18	8.56E-14	0.00E+00
11	$^4G_{5/2}$	→	$^4I_{9/2}$	0.00	-4587	1.37E-06	5.66E-13	1.37E-06	0.00E+00
12	$^4G_{7/2}$	→	$^4I_{9/2}$	0.00	-2722	1.79E-03	1.77E-09	1.79E-03	0.00E+00
13	$^4G_{9/2}$	→	$^4I_{9/2}$	0.00	-2306	1.52E-03	4.24E-09	1.52E-03	0.00E+00
14	$^2K_{13/2}$	→	$^4I_{9/2}$	0.00	-2230	1.21E-03	2.11E-09	1.21E-03	0.00E+00
15	$^2D_{3/2}$	→	$^4I_{9/2}$	1.10	-590	4.75E+00	1.25E-05	4.75E+00	0.00E+00
16	$^4G_{11/2}$	→	$^4I_{9/2}$	0.32	-301	1.37E+00	1.14E-05	1.37E+00	0.00E+00
17	$^2K_{15/2}$	→	$^4I_{9/2}$	0.32	-235	1.39E+00	1.85E-05	1.39E+00	0.00E+00
18	$^2P_{1/2}$	→	$^4I_{9/2}$	97.95	1443	4.21E+02	1.10E-03	4.21E+02	0.00E+00
19	$^2D_{5/2}$	→	$^4I_{9/2}$	0.09	1989	3.69E-01	1.27E-05	3.69E-01	0.00E+00
20	$^2P_{3/2}$	→	$^4I_{9/2}$	0.22	4409	9.31E-01	4.18E-06	9.31E-01	0.00E+00

*Table S6. Forward (^{3}MLCT to Nd^{III}) intramolecular energy transfer rates (W, s^{-1}) calculated for the **NdRu-3** complex. Δ (cm^{-1}) is the donor-acceptor energy difference, W(%) is the percentage of the pathway, W_{total} is the sum of the dipole-dipole ($W_{d–d}$), dipole-multipole ($W_{d–m}$), and exchange (W_{ex}) rates of the mechanisms.*

**Pathway**	**Transition**			***W*(%)**	**Δ**	W_{total}	$W_{d–d}$	$W_{d–m}$	W_{ex}
1	$^4I_{9/2}$	→	$^4I_{11/2}$	0.00	14481	1.50E-08	8.60E-14	1.50E-08	0.00E+00
2	$^4I_{9/2}$	→	$^4I_{13/2}$	0.00	12497	6.39E-08	1.96E-12	6.39E-08	0.00E+00
3	$^4I_{9/2}$	→	$^4I_{15/2}$	0.00	10447	8.05E-09	7.13E-12	8.04E-09	0.00E+00
4	$^4I_{9/2}$	→	$^4F_{3/2}$	14.10	4974	2.60E-02	2.25E-08	2.60E-02	0.00E+00
5	$^4I_{9/2}$	→	$^4F_{5/2}$	29.86	3935	5.51E-02	1.20E-07	5.51E-02	0.00E+00
6	$^4I_{9/2}$	→	$^2H_{9/2}$	7.26	3827	1.34E-02	3.08E-08	1.34E-02	0.00E+00
7	$^4I_{9/2}$	→	$^4F_{7/2}$	9.73	2976	1.79E-02	1.63E-07	1.79E-02	0.00E+00
8	$^4I_{9/2}$	→	$^4S_{3/2}$	0.00	15226	3.70E-11	2.94E-15	3.70E-11	0.00E+00
9	$^4I_{9/2}$	→	$^4F_{9/2}$	4.52	1696	8.33E-03	2.71E-08	8.33E-03	0.00E+00
10	$^4I_{9/2}$	→	$^2H_{11/2}$	1.11	490	2.04E-03	8.50E-09	2.04E-03	0.00E+00
11	$^4I_{9/2}$	→	$^4G_{5/2}$	33.44	-833	6.17E-02	1.51E-08	6.17E-02	0.00E+00
12	$^4I_{9/2}$	→	$^4G_{7/2}$	0.00	-2698	4.43E-07	2.12E-13	4.43E-07	0.00E+00
13	$^4I_{9/2}$	→	$^4G_{9/2}$	0.00	-3114	8.60E-09	8.32E-15	8.60E-09	0.00E+00
14	$^4I_{9/2}$	→	$^2K_{13/2}$	0.00	-3190	2.48E-09	2.69E-15	2.48E-09	0.00E+00
15	$^4I_{9/2}$	→	$^2D_{3/2}$	0.00	-4830	1.93E-13	1.25E-19	1.93E-13	0.00E+00
16	$^4I_{9/2}$	→	$^4G_{11/2}$	0.00	-5119	9.91E-15	2.02E-20	9.91E-15	0.00E+00
17	$^4I_{9/2}$	→	$^2K_{15/2}$	0.00	-5185	6.85E-15	2.24E-20	6.85E-15	0.00E+00
18	$^4I_{9/2}$	→	$^2P_{1/2}$	0.00	-6863	3.33E-18	2.14E-24	3.33E-18	0.00E+00
19	$^4I_{9/2}$	→	$^2D_{5/2}$	0.00	-7409	6.69E-22	5.62E-27	6.69E-22	0.00E+00
20	$^4I_{9/2}$	→	$^2P_{3/2}$	0.00	-9829	1.13E-27	1.25E-33	1.13E-27	0.00E+00

*Table S7. Backward (Nd^{III} to ^{3}MLCT) intramolecular energy transfer rates (W, s^{-1}) calculated for the **NdRu-3** complex. Δ (cm^{-1}) is the donor-acceptor energy difference, W(%) is the percentage of the pathway, W_{total} is the sum of the dipole-dipole ($W_{d–d}$), dipole-multipole ($W_{d–m}$), and exchange (W_{ex}) rates of the mechanisms.*

**Pathway**	**Transition**			***W*(%)**	**Δ**	W_{total}	$W_{d–d}$	$W_{d–m}$	W_{ex}
1	$^4I_{11/2}$	→	$^4I_{9/2}$	0.00	-14481	5.48E-39	3.15E-44	5.48E-39	0.00E+00
2	$^4I_{13/2}$	→	$^4I_{9/2}$	0.00	-12497	2.85E-34	8.73E-39	2.85E-34	0.00E+00
3	$^4I_{15/2}$	→	$^4I_{9/2}$	0.00	-10447	6.16E-31	5.45E-34	6.15E-31	0.00E+00
4	$^4F_{3/2}$	→	$^4I_{9/2}$	0.00	-4974	2.94E-12	2.54E-18	2.94E-12	0.00E+00
5	$^4F_{5/2}$	→	$^4I_{9/2}$	0.00	-3935	5.61E-10	1.23E-15	5.61E-10	0.00E+00
6	$^2H_{9/2}$	→	$^4I_{9/2}$	0.00	-3827	1.28E-10	2.94E-16	1.28E-10	0.00E+00
7	$^4F_{7/2}$	→	$^4I_{9/2}$	0.00	-2976	1.34E-08	1.21E-13	1.34E-08	0.00E+00
8	$^4S_{3/2}$	→	$^4I_{9/2}$	0.00	-15226	1.37E-42	1.08E-46	1.37E-42	0.00E+00
9	$^4F_{9/2}$	→	$^4I_{9/2}$	0.00	-1696	2.32E-06	7.56E-12	2.32E-06	0.00E+00
10	$^2H_{11/2}$	→	$^4I_{9/2}$	0.00	-490	1.57E-04	6.54E-10	1.57E-04	0.00E+00
11	$^4G_{5/2}$	→	$^4I_{9/2}$	95.22	833	6.18E+00	1.51E-06	6.18E+00	0.00E+00
12	$^4G_{7/2}$	→	$^4I_{9/2}$	3.96	2698	2.57E-01	1.23E-07	2.57E-01	0.00E+00
13	$^4G_{9/2}$	→	$^4I_{9/2}$	0.45	3114	2.89E-02	2.79E-08	2.89E-02	0.00E+00
14	$^2K_{13/2}$	→	$^4I_{9/2}$	0.13	3190	8.33E-03	9.04E-09	8.33E-03	0.00E+00
15	$^2D_{3/2}$	→	$^4I_{9/2}$	0.12	4830	7.69E-03	4.98E-09	7.69E-03	0.00E+00
16	$^4G_{11/2}$	→	$^4I_{9/2}$	0.01	5119	4.34E-04	8.84E-10	4.34E-04	0.00E+00
17	$^2K_{15/2}$	→	$^4I_{9/2}$	0.01	5185	3.02E-04	9.89E-10	3.02E-04	0.00E+00
18	$^2P_{1/2}$	→	$^4I_{9/2}$	0.11	6863	7.25E-03	4.66E-09	7.25E-03	0.00E+00
19	$^2D_{5/2}$	→	$^4I_{9/2}$	0.00	7409	4.06E-06	3.41E-11	4.06E-06	0.00E+00
20	$^2P_{3/2}$	→	$^4I_{9/2}$	0.00	9829	1.35E-06	1.49E-12	1.35E-06	0.00E+00

Table S8. Forward (^{1}MLCT to Er^{III}) intramolecular energy transfer rates (W, s^{-1}) calculated for the ***ErRu-3*** *complex. Δ (cm^{-1}) is the donor-acceptor energy difference, W(%) is the percentage of the pathway, W_{total} is the sum of the dipole-dipole (W_{d-d}), dipole-multipole (W_{d-m}), and exchange (W_{ex}) rates of the mechanisms.*

Pathway	Transition			*W*(%)	Δ	W_{total}	W_{d-d}	W_{d-m}	W_{ex}
1	$^4I_{15/2}$	→	$^4I_{13/2}$	0.00	15285	2.24E-07	4.38E-15	2.24E-07	0.00E+00
2	$^4I_{15/2}$	→	$^4I_{11/2}$	0.00	11651	1.72E-04	2.86E-12	1.72E-04	0.00E+00
3	$^4I_{15/2}$	→	$^4I_{9/2}$	0.01	9400	1.56E-02	2.45E-11	1.56E-02	0.00E+00
4	$^4I_{15/2}$	→	$^4F_{9/2}$	1.46	6542	1.81E+00	5.65E-09	1.81E+00	0.00E+00
5	$^4I_{15/2}$	→	$^4S_{3/2}$	0.00	3414	1.04E-03	1.57E-08	1.04E-03	0.00E+00
6	$^4I_{15/2}$	→	$^2H_{11/2}$	89.31	2660	1.11E+02	5.04E-07	1.11E+02	0.00E+00
7	$^4I_{15/2}$	→	$^4F_{7/2}$	9.22	1282	1.15E+01	1.13E-07	1.15E+01	0.00E+00
8	$^4I_{15/2}$	→	$^4F_{5/2}$	0.00	-379	4.04E-04	6.12E-09	4.04E-04	0.00E+00
9	$^4I_{15/2}$	→	$^4F_{3/2}$	0.00	-715	4.38E-05	6.64E-10	4.38E-05	0.00E+00
10	$^4I_{15/2}$	→	$^4H_{9/2}$	0.00	-2759	1.98E-06	3.71E-14	1.98E-06	0.00E+00
11	$^4I_{15/2}$	→	$^4G_{11/2}$	0.00	-4634	9.07E-09	4.13E-17	9.07E-09	0.00E+00
12	$^4I_{15/2}$	→	$^4G_{9/2}$	0.00	-5640	2.71E-12	7.11E-21	2.71E-12	0.00E+00
13	$^4I_{15/2}$	→	$^2K_{15/2}$	0.00	-5925	1.27E-13	9.48E-22	1.27E-13	0.00E+00
14	$^4I_{15/2}$	→	$^4G_{7/2}$	0.00	-6227	7.97E-15	1.03E-22	7.97E-15	0.00E+00
15	$^4I_{15/2}$	→	$^2P_{1/2}$	0.00	-11322	0.00E+00	0.00E+00	0.00E+00	0.00E+00

Table S9. Backward (Er^{III} to ^{1}MLCT) intramolecular energy transfer rates (W, s^{-1}) calculated for the ***ErRu-3*** *complex. Δ (cm^{-1}) is the donor-acceptor energy difference, W(%) is the percentage of the pathway, W_{total} is the sum of the dipole-dipole (W_{d-d}), dipole-multipole (W_{d-m}), and exchange (W_{ex}) rates of the mechanisms.*

Pathway	Transition			*W*(%)	Δ	W_{total}	W_{d-d}	W_{d-m}	W_{ex}
1	$^4I_{13/2}$	→	$^4I_{15/2}$	0.00	-15285	2.39E-39	4.67E-47	2.39E-39	0.00E+00
2	$^4I_{11/2}$	→	$^4I_{15/2}$	0.00	-11651	8.97E-29	1.49E-36	8.97E-29	0.00E+00
3	$^4I_{9/2}$	→	$^4I_{15/2}$	0.00	-9400	5.18E-22	8.14E-31	5.18E-22	0.00E+00
4	$^4F_{9/2}$	→	$^4I_{15/2}$	0.00	-6542	5.87E-14	1.83E-22	5.87E-14	0.00E+00
5	$^4S_{3/2}$	→	$^4I_{15/2}$	0.00	-3414	3.63E-10	5.50E-15	3.63E-10	0.00E+00
6	$^2H_{11/2}$	→	$^4I_{15/2}$	0.00	-2660	4.02E-04	1.83E-12	4.02E-04	0.00E+00
7	$^4F_{7/2}$	→	$^4I_{15/2}$	0.07	-1282	5.05E-02	4.97E-10	5.05E-02	0.00E+00
8	$^4F_{5/2}$	→	$^4I_{15/2}$	0.01	379	7.54E-03	1.14E-07	7.54E-03	0.00E+00
9	$^4F_{3/2}$	→	$^4I_{15/2}$	0.01	715	6.90E-03	1.05E-07	6.90E-03	0.00E+00
10	$^4H_{9/2}$	→	$^4I_{15/2}$	2.89	2759	2.00E+00	3.75E-08	2.00E+00	0.00E+00
11	$^4G_{11/2}$	→	$^4I_{15/2}$	91.96	4634	6.37E+01	2.90E-07	6.37E+01	0.00E+00
12	$^4G_{9/2}$	→	$^4I_{15/2}$	4.30	5640	2.98E+00	7.83E-09	2.98E+00	0.00E+00
13	$^2K_{15/2}$	→	$^4I_{15/2}$	0.48	5925	3.31E-01	2.48E-09	3.31E-01	0.00E+00
14	$^4G_{7/2}$	→	$^4I_{15/2}$	0.28	6227	1.92E-01	2.48E-09	1.92E-01	0.00E+00
15	$^2P_{1/2}$	→	$^4I_{15/2}$	0.00	11322	0.00E+00	0.00E+00	0.00E+00	0.00E+00

*Table S10. Forward (^{3}MLCT to Er^{III}) intramolecular energy transfer rates (W, s^{-1}) calculated for the **ErRu-3** complex. Δ (cm^{-1}) is the donor-acceptor energy difference, W(%) is the percentage of the pathway, W_{total} is the sum of the dipole-dipole (W_{d-d}), dipole-multipole (W_{d-m}), and exchange (W_{ex}) rates of the mechanisms.*

Pathway	Transition			W(%)	Δ	W_{total}	W_{d-d}	W_{d-m}	W_{ex}
1	$^4I_{15/2}$	→	$^4I_{13/2}$	0.01	9865	3.71E-06	2.15E-14	3.71E-06	0.00E+00
2	$^4I_{15/2}$	→	$^4I_{11/2}$	0.25	6231	6.57E-05	6.76E-13	6.57E-05	0.00E+00
3	$^4I_{15/2}$	→	$^4I_{9/2}$	8.54	3980	2.29E-03	8.85E-13	2.29E-03	0.00E+00
4	$^4I_{15/2}$	→	$^4F_{9/2}$	91.20	1122	2.45E-02	1.88E-11	2.45E-02	0.00E+00
5	$^4I_{15/2}$	→	$^4S_{3/2}$	0.00	-2006	1.64E-10	2.40E-16	1.64E-10	0.00E+00
6	$^4I_{15/2}$	→	$^2H_{11/2}$	0.00	-2760	4.91E-08	1.08E-16	4.91E-08	0.00E+00
7	$^4I_{15/2}$	→	$^4F_{7/2}$	0.00	-4138	4.08E-12	9.88E-21	4.08E-12	0.00E+00
8	$^4I_{15/2}$	→	$^4F_{5/2}$	0.00	-5799	1.88E-19	2.75E-25	1.88E-19	0.00E+00
9	$^4I_{15/2}$	→	$^4F_{3/2}$	0.00	-6135	1.55E-20	2.26E-26	1.55E-20	0.00E+00
10	$^4I_{15/2}$	→	$^4H_{9/2}$	0.00	-8179	4.98E-23	2.29E-31	4.98E-23	0.00E+00
11	$^4I_{15/2}$	→	$^4G_{11/2}$	0.00	-10054	2.42E-26	5.32E-35	2.42E-26	0.00E+00
12	$^4I_{15/2}$	→	$^4G_{9/2}$	0.00	-11060	6.13E-30	3.96E-39	6.13E-30	0.00E+00
13	$^4I_{15/2}$	→	$^2K_{15/2}$	0.00	-11345	9.84E-32	4.16E-40	9.84E-32	0.00E+00
14	$^4I_{15/2}$	→	$^4G_{7/2}$	0.00	-11647	1.11E-32	3.52E-41	1.11E-32	0.00E+00
15	$^4I_{15/2}$	→	$^2P_{1/2}$	0.00	-16742	0.00E+00	0.00E+00	0.00E+00	0.00E+00

*Table S11. Backward (Er^{III} to ^{3}MLCT) intramolecular energy transfer rates (W, s^{-1}) calculated for the **ErRu-3** complex. Δ (cm^{-1}) is the donor-acceptor energy difference, W(%) is the percentage of the pathway, W_{total} is the sum of the dipole-dipole (W_{d-d}), dipole-multipole (W_{d-m}), and exchange (W_{ex}) rates of the mechanisms.*

Pathway	Transition			W(%)	Δ	W_{total}	W_{d-d}	W_{d-m}	W_{ex}
1	$^4I_{13/2}$	→	$^4I_{15/2}$	0.00	-9865	9.04E-27	5.25E-35	9.04E-27	0.00E+00
2	$^4I_{11/2}$	→	$^4I_{15/2}$	0.00	-6231	7.82E-18	8.06E-26	7.82E-18	0.00E+00
3	$^4I_{9/2}$	→	$^4I_{15/2}$	0.00	-3980	1.74E-11	6.73E-21	1.74E-11	0.00E+00
4	$^4F_{9/2}$	→	$^4I_{15/2}$	0.40	-1122	1.82E-04	1.39E-13	1.82E-04	0.00E+00
5	$^4S_{3/2}$	→	$^4I_{15/2}$	0.03	2006	1.31E-05	1.92E-11	1.31E-05	0.00E+00
6	$^2H_{11/2}$	→	$^4I_{15/2}$	90.33	2760	4.07E-02	8.94E-11	4.07E-02	0.00E+00
7	$^4F_{7/2}$	→	$^4I_{15/2}$	9.13	4138	4.11E-03	9.95E-12	4.11E-03	0.00E+00
8	$^4F_{5/2}$	→	$^4I_{15/2}$	0.00	5799	8.05E-07	1.18E-12	8.05E-07	0.00E+00
9	$^4F_{3/2}$	→	$^4I_{15/2}$	0.00	6135	5.57E-07	8.13E-13	5.57E-07	0.00E+00
10	$^4H_{9/2}$	→	$^4I_{15/2}$	0.03	8179	1.15E-05	5.29E-14	1.15E-05	0.00E+00
11	$^4G_{11/2}$	→	$^4I_{15/2}$	0.09	10054	3.89E-05	8.55E-14	3.89E-05	0.00E+00
12	$^4G_{9/2}$	→	$^4I_{15/2}$	0.00	11060	1.54E-06	9.97E-16	1.54E-06	0.00E+00
13	$^2K_{15/2}$	→	$^4I_{15/2}$	0.00	11345	5.88E-08	2.49E-16	5.88E-08	0.00E+00
14	$^4G_{7/2}$	→	$^4I_{15/2}$	0.00	11647	6.10E-08	1.94E-16	6.10E-08	0.00E+00
15	$^2P_{1/2}$	→	$^4I_{15/2}$	0.00	16742	0.00E+00	0.00E+00	0.00E+00	0.00E+00

*Table S12. Forward (^{1}MLCT to Yb^{III}) intramolecular energy transfer rates (W, s^{-1}) calculated for the **YbRu-3** complex. Δ (cm^{-1}) is the donor-acceptor energy difference, W(%) is the percentage of the pathway, W_{total} is the sum of the dipole-dipole (W_{d-d}), dipole-multipole (W_{d-m}), and exchange (W_{ex}) rates of the mechanisms.*

Pathway	Transition			*W*(%)	Δ	W_{total}	W_{d-d}	W_{d-m}	W_{ex}
1	$^2F_{7/2}$	→	$^2F_{5/2}$	100	11540	3.04E-01	1.60E-11	3.77E-03	3.00E-01

*Table S13. Backward (Yb^{III} to ^{1}MLCT) intramolecular energy transfer rates (W, s^{-1}) calculated for the **YbRu-3** complex. Δ (cm^{-1}) is the donor-acceptor energy difference, W(%) is the percentage of the pathway, W_{total} is the sum of the dipole-dipole (W_{d-d}), dipole-multipole (W_{d-m}), and exchange (W_{ex}) rates of the mechanisms.*

Pathway	Transition			*W*(%)	Δ	W_{total}	W_{d-d}	W_{d-m}	W_{ex}
1	$^2F_{5/2}$	→	$^2F_{7/2}$	100	-11540	2.78E-25	1.47E-35	3.45E-27	2.74E-25

*Table S14. Forward (^{3}MLCT to Yb^{III}) intramolecular energy transfer rates (W, s^{-1}) calculated for the **YbRu-3** complex. Δ (cm^{-1}) is the donor-acceptor energy difference, W(%) is the percentage of the pathway, W_{total} is the sum of the dipole-dipole (W_{d-d}), dipole-multipole (W_{d-m}), and exchange (W_{ex}) rates of the mechanisms.*

Pathway	Transition			*W*(%)	Δ	W_{total}	W_{d-d}	W_{d-m}	W_{ex}
1	$^2F_{7/2}$	→	$^2F_{5/2}$	100	6120	2.09E+03	3.46E-12	2.45E-03	2.09E+03

*Table S15. Backward (Yb^{III} to ^{3}MLCT) intramolecular energy transfer rates (W, s^{-1}) calculated for the **YbRu-3** complex. Δ (cm^{-1}) is the donor-acceptor energy difference, W(%) is the percentage of the pathway, W_{total} is the sum of the dipole-dipole (W_{d-d}), dipole-multipole (W_{d-m}), and exchange (W_{ex}) rates of the mechanisms.*

Pathway	Transition			*W*(%)	Δ	W_{total}	W_{d-d}	W_{d-m}	W_{ex}
1	$^2F_{5/2}$	→	$^2F_{7/2}$	100	-6120	4.36E-10	7.23E-25	5.12E-16	4.36E-10

References

[1] C. Y. Chow, S. V. Eliseeva, E. R. Trivedi, T. N. Nguyen, J. W. Kampf, S. Petoud, V. L. Pecoraro, *J. Am. Chem. Soc.* **2016**, *138*, 5100–5109.
[2] C. Tang, L. Li, Z. Pang, Z. Yuan, *Chin. J. Chem.* **2009**, *27*, 195–201.
[3] R. B. P. Elmes, K. N. Orange, S. M. Cloonan, D. C. Williams, T. Gunnlaugsson, *J. Am. Chem. Soc.* **2011**, *133*, 15862–15865.
[4] E. R. Trivedi, V. L. Pecoraro, S. V. Eliseeva, S. Petoud, C. Y. Chow, T. N. Nguyen, J. C. Lutter, I. Martinic, *Ln(Iii) and Ga(Iii) Metallacrown Complexes*, **2016**, WO2016166380A1.
[5] H. L. Chan, L. Lyu, J. Aw, W. Zhang, J. Li, H.-H. Yang, H. Hayashi, S. Chiba, B. Xing, *ACS Chem. Biol.* **2018**, *13*, 1890–1896.
[6] U. S. Gunay, M. Cetin, O. Daglar, G. Hizal, U. Tunca, H. Durmaz, *Polym. Chem.* **2018**, *9*, 3037–3054.
[7] C. D. Ellis, L. D. Margerum, R. W. Murray, T. J. Meyer, *Inorg. Chem.* **1983**, *22*, 1283–1291.
[8] J. C. Lutter, B. A. Lopez Bermudez, T. N. Nguyen, J. W. Kampf, V. L. Pecoraro, *J. Inorg. Biochem.* **2019**, *192*, 119–125.
[9] J.-C. G. Bünzli, S. V. Eliseeva, in *Lanthan. Lumin. Photophysical Anal. Biol. Asp.* (Eds.: P. Hänninen, H. Härmä), Springer, Berlin, Heidelberg, **2011**, pp. 1–45.
[10] R. Pleijhuis, A. Timmermans, J. D. Jong, E. D. Boer, V. Ntziachristos, G. V. Dam, *JoVE J. Vis. Exp.* **2014**, e51776.
[11] G. Collet, A. Hrvat, S. V. Eliseeva, C. Besnard, A. Kovalenko, S. Petoud, *Chem. Commun.* **2021**, *57*, 3351–3354.
[12] S. Grimme, *J. Chem. Theory Comput.* **2019**, *15*, 2847–2862.
[13] P. Pracht, F. Bohle, S. Grimme, *Phys. Chem. Chem. Phys.* **2020**, *22*, 7169–7192.
[14] C. Bannwarth, S. Ehlert, S. Grimme, *J. Chem. Theory Comput.* **2019**, *15*, 1652–1671.
[15] S. Ehlert, M. Stahn, S. Spicher, S. Grimme, *J. Chem. Theory Comput.* **2021**, *17*, 4250–4261.
[16] M. Leonzio, A. Melchior, G. Faura, M. Tolazzi, M. Bettinelli, F. Zinna, L. Arrico, L. D. Bari, F. Piccinelli, *New J. Chem.* **2018**, *42*, 7931–7939.
[17] A. N. Carneiro Neto, R. T. Moura, L. D. Carlos, O. L. Malta, M. Sanadar, A. Melchior, E. Kraka, S. Ruggieri, M. Bettinelli, F. Piccinelli, *Inorg. Chem.* **2022**, *61*, 16333–16346.
[18] E. V. Salerno, A. N. Carneiro Neto, S. V. Eliseeva, M. A. Hernández-Rodríguez, J. C. Lutter, T. Lathion, J. W. Kampf, S. Petoud, L. D. Carlos, V. L. Pecoraro, *J. Am. Chem. Soc.* **2022**, *144*, 18259–18271.
[19] J. C. Lutter, S. V. Eliseeva, G. Collet, I. Martinić, J. W. Kampf, B. L. Schneider, A. Carichner, J. Sobilo, S. Lerondel, S. Petoud, V. L. Pecoraro, *Chem. – Eur. J.* **2020**, *26*, 1274–1277.
[20] T. N. Nguyen, C. Y. Chow, S. V. Eliseeva, E. R. Trivedi, J. W. Kampf, I. Martinić, S. Petoud, V. L. Pecoraro, *Chem. – Eur. J.* **2018**, *24*, 1031–1035.

[21] W. M. Haynes, Ed. , *CRC Handbook of Chemistry and Physics*, CRC Press, Boca Raton, **2016**.
[22] S. Grimme, J. Antony, S. Ehrlich, H. Krieg, *J. Chem. Phys.* **2010**, *132*, 154104.
[23] S. Grimme, S. Ehrlich, L. Goerigk, *J. Comput. Chem.* **2011**, *32*, 1456–1465.
[24] J. P. Perdew, K. Burke, M. Ernzerhof, *Phys. Rev. Lett.* **1996**, *77*, 3865–3868.
[25] J. D. Rolfes, F. Neese, D. A. Pantazis, *J. Comput. Chem.* **2020**, *41*, 1842–1849.
[26] F. Weigend, R. Ahlrichs, *Phys. Chem. Chem. Phys.* **2005**, *7*, 3297–3305.
[27] C. van Wüllen, *J. Chem. Phys.* **1998**, *109*, 392–399.
[28] A. V. Marenich, C. J. Cramer, D. G. Truhlar, *J. Phys. Chem. B* **2009**, *113*, 6378–6396.
[29] B. de Souza, G. Farias, F. Neese, R. Izsák, *J. Chem. Theory Comput.* **2019**, *15*, 1896–1904.
[30] C. Adamo, V. Barone, *J. Chem. Phys.* **1999**, *110*, 6158–6170.
[31] Z. Liu, T. Lu, Q. Chen, *Carbon* **2020**, *165*, 461–467.
[32] F. Neese, F. Wennmohs, A. Hansen, U. Becker, *Chem. Phys.* **2009**, *356*, 98–109.
[33] R. Izsák, F. Neese, *J. Chem. Phys.* **2011**, *135*, 144105.
[34] F. Weigend, *Phys. Chem. Chem. Phys.* **2006**, *8*, 1057–1065.
[35] F. Neese, *WIREs Comput. Mol. Sci.* **2022**, *12*, e1606.
[36] C. Bannwarth, E. Caldeweyher, S. Ehlert, A. Hansen, P. Pracht, J. Seibert, S. Spicher, S. Grimme, *WIREs Comput. Mol. Sci.* **2021**, *11*, e1493.
[37] T. Lu, F. Chen, *J. Comput. Chem.* **2012**, *33*, 580–592.
[38] R. T. Moura Jr., A. N. Carneiro Neto, E. C. Aguiar, C. V. Santos-Jr., E. M. de Lima, W. M. Faustino, E. E. S. Teotonio, H. F. Brito, M. C. F. C. Felinto, R. A. S. Ferreira, L. D. Carlos, R. L. Longo, O. L. Malta, *Opt. Mater. X* **2021**, *11*, 100080.
[39] A. N. Carneiro Neto, E. E. S. Teotonio, G. F. de Sá, H. F. Brito, J. Legendziewicz, L. D. Carlos, M. C. F. C. Felinto, P. Gawryszewska, R. T. Moura, R. L. Longo, W. M. Faustino, O. L. Malta, in *Handb. Phys. Chem. Rare Earths* (Eds.: J.-C.G. Bünzli, V.K. Pecharsky), Elsevier, **2019**, pp. 55–162.
[40] T. Lazarides, D. Sykes, S. Faulkner, A. Barbieri, M. D. Ward, *Chem. – Eur. J.* **2008**, *14*, 9389–9399.
[41] J. A. A. Coelho, R. T. Moura, R. L. Longo, O. L. Malta, A. N. Carneiro Neto, *Inorganics* **2023**, *11*, 38.
